# *scShapes:* a statistical framework for identifying distribution shapes in single-cell RNA-sequencing data

**DOI:** 10.1093/gigascience/giac126

**Published:** 2023-01-24

**Authors:** Malindrie Dharmaratne, Ameya S Kulkarni, Atefeh Taherian Fard, Jessica C Mar

**Affiliations:** Australian Institute for Bioengineering and Nanotechnology, The University of Queensland, Brisbane, QLD, 4072, Australia; Institute for Aging Research, Albert Einstein College of Medicine, Bronx, New York, NY 10461, USA; Department of Medicine, Division of Endocrinology, Albert Einstein College of Medicine, Bronx, New York, NY 10461, USA; Australian Institute for Bioengineering and Nanotechnology, The University of Queensland, Brisbane, QLD, 4072, Australia; Australian Institute for Bioengineering and Nanotechnology, The University of Queensland, Brisbane, QLD, 4072, Australia

**Keywords:** single-cell RNA-sequencing, distribution shapes, zero inflation

## Abstract

**Background:**

Single-cell RNA sequencing (scRNA-seq) methods have been advantageous for quantifying cell-to-cell variation by profiling the transcriptomes of individual cells. For scRNA-seq data, variability in gene expression reflects the degree of variation in gene expression from one cell to another. Analyses that focus on cell–cell variability therefore are useful for going beyond changes based on average expression and, instead, identifying genes with homogeneous expression versus those that vary widely from cell to cell.

**Results:**

We present a novel statistical framework, *scShapes*, for identifying differential distributions in single-cell RNA-sequencing data using generalized linear models. Most approaches for differential gene expression detect shifts in the mean value. However, as single-cell data are driven by overdispersion and dropouts, moving beyond means and using distributions that can handle excess zeros is critical. *scShapes* quantifies gene-specific cell-to-cell variability by testing for differences in the expression distribution while flexibly adjusting for covariates if required. We demonstrate that *scShapes* identifies subtle variations that are independent of altered mean expression and detects biologically relevant genes that were not discovered through standard approaches.

**Conclusions:**

This analysis also draws attention to genes that switch distribution shapes from a unimodal distribution to a zero-inflated distribution and raises open questions about the plausible biological mechanisms that may give rise to this, such as transcriptional bursting. Overall, the results from *scShapes* help to expand our understanding of the role that gene expression plays in the transcriptional regulation of a specific perturbation or cellular phenotype. Our framework *scShapes* is incorporated into a Bioconductor R package (https://www.bioconductor.org/packages/release/bioc/html/scShapes.html).

## Background

Variation in gene expression from one cell to another plays an instrumental role in how tissues develop and function [1], and therefore consideration of the distribution shape of a gene's expression profile is important for understanding transcriptional regulation [2]. The analysis of single-cell RNA sequencing (scRNA-seq) datasets has provided a means to quantitatively study cell-to-cell variation in response to phenotypic changes, and as a result, new rare and complex cell populations have been identified [3], regulatory relationships among genes have been discovered [4], and trajectories of cell lineages in development and disease have been elucidated [5]. However, a major limitation of existing scRNA-seq methods is that these analyses tend to focus on detecting changes in average expression rather than changes in variability or other properties of the distribution. While transcriptional regulation requires both, a significant strength of scRNA-seq data is the opportunity to model gene expression variability and its role in regulating cellular phenotypes.

Existing scRNA-seq methods assume that a single parametric distribution is adequate for modeling the shape of a gene's expression profile. While this assumption may have practical advantages, it does limit the ability to discover new relationships or gene expression shape patterns. A property of scRNA-seq data is its increased sparsity compared to bulk gene expression data, where an abundance of cells may have unobserved expression levels due to either biological or technical sources of cell-to-cell variability [6, 7]. As a result, it may be an oversimplification to assume that the expression profiles of genes across the transcriptome may be adequately explained by a single parametric distribution. A limitation of this assumption may be that some genes are overlooked or modeled suboptimally as we fail to first evaluate the prevalence of different distributions in a scRNA-seq dataset [2].

To model the heterogeneity of gene expression data under a statistical framework, it is vital that the distribution with the most appropriate fit for each gene's expression profile be used [8]. While some statistical methods have appealed to the use of mixture models [6, 9] as an alternative to the widely used negative binomial distribution [10, 11], they fail to investigate the range of different gene expression distributions that may be present in the scRNA-seq data as a first step. In *scShapes*, we address this issue directly by proposing a gene-specific framework for identifying differential distributions from a selection of relevant candidate distributions.

Currently, differentially distributed genes can be assessed using the *R* package *scDD* [12]. However, this approach is unable to adjust for covariates directly in the model. Because accounting for covariates, especially technical ones like gender or hospital site, may be necessary to address for the underlying question being asked of the scRNA-seq analysis, this limitation of *scDD* has practical implications as the size and complexity of scRNA-seq datasets grow. The *scDD* method also only permits pairwise comparisons between 2 biological conditions. In our *scShapes* framework, we propose an approach for modeling read counts from droplet-based scRNA-seq experiments using generalized linear models (GLMs) with error distributions belonging to the family of zero-inflated negative binomial distributions.

**Figure 1:**
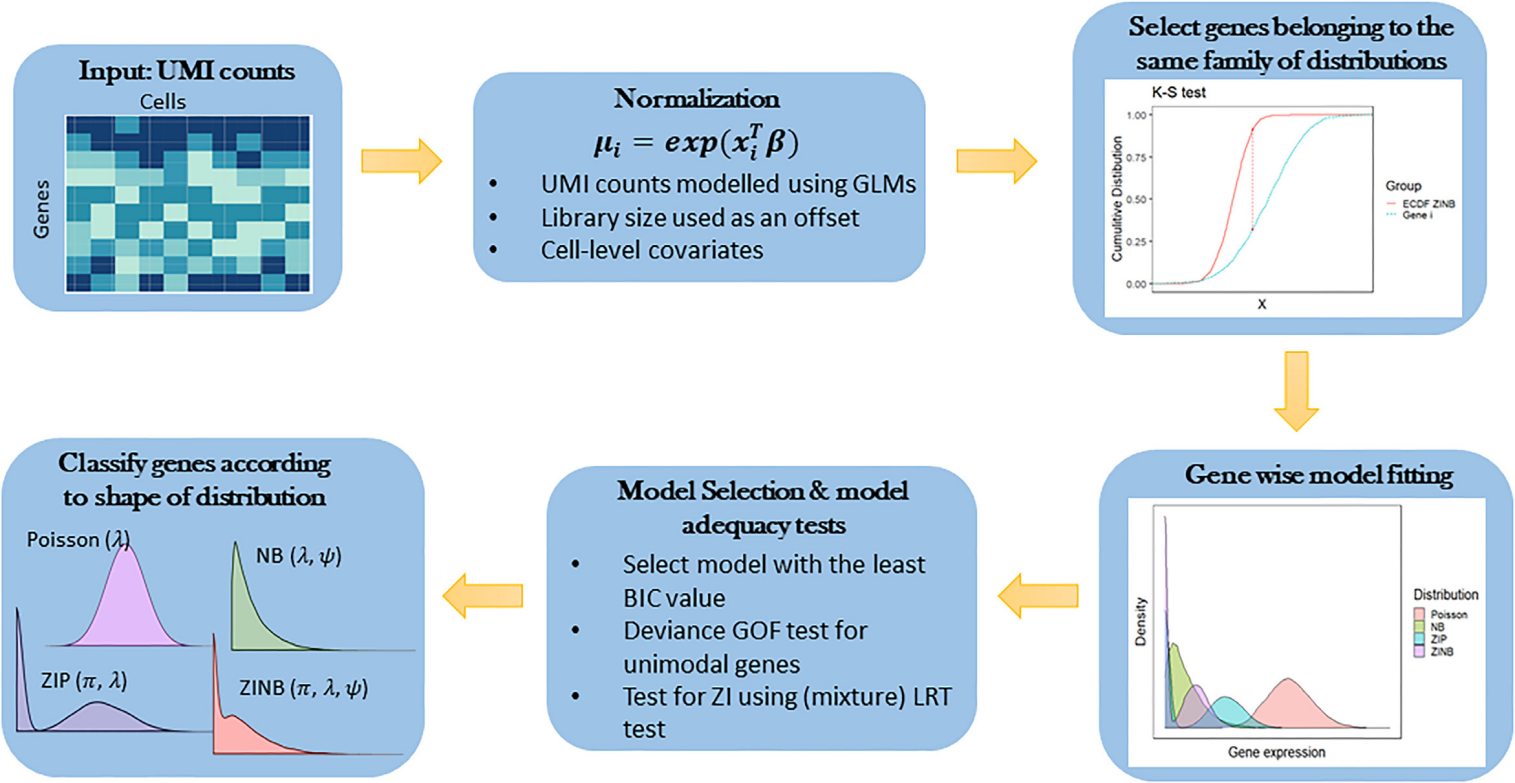
*scShapes* pipeline for identifying gene expression according to distribution shape. The input to *scShapes* is a matrix of UMI counts with initial quality control performed to remove lowly expressed genes. A Kolmogorov–Smirnov test is first performed to select genes belonging to the family of ZINB distributions. Genes that do not belong to this category are removed from further analysis. For genes that are assumed to follow a distribution from the ZINB family, the best distribution that explains most of the heterogeneity in its gene expression values is selected from Poisson, NB, ZIP, and ZINB distributions based on 2 rounds of model adequacy testing.

Zero counts are a prevalent feature of scRNA-seq data that present challenges for statistical modeling, mainly because these zero values result from different zero generating processes within the same biological system [13]. Because these zeros can arise from biological and technical sources, the treatment of the zero values (e.g., through imputation or filtering) is not straightforward. The zero values observed in scRNA-seq can be the result of limitations in sequencing depth and capture efficiency, such that only a small percentage of the transcripts present ends up being counted and low-abundance transcripts can go undetected [14, 15]. Observing a zero count can also be due to biological factors like a gene that is simply not expressed in a specific cell type or state or the fact that transcription is a stochastic process that gives rise to these additional zeros [16]. By taking all these factors into account, we assume that the overabundance of zeros in scRNA-seq data is gene specific and that each gene therefore requires a statistical model with its own set of distributional assumptions. To implement this, our framework models gene expression data for a mix of unimodal and zero-inflated distributions to identify the distribution that provides the most appropriate fit for each gene (Fig. 1).

Here we develop a framework, *scShapes*, which relies upon modeling read counts using GLMs and matches a distribution shape to a gene's expression distribution for an individual gene. The gene expression distribution is modeled using both unimodal and zero-inflated distributions, where for accurate evaluation of zero inflation, we conduct a modified likelihood ratio test (LRT) [17]. Our framework has been designed to identify subtle variations from one cell to another that is not weighted toward a change in mean. It also has the flexibility to adjust for covariates and perform multiple comparisons between sample or treatment groups while explicitly modeling the variability of gene expression occurring between cells. Using simulation studies, we show that *scShapes* can reliably detect zero-inflated genes, and when applied to a range of scRNA-seq published datasets, *scShapes* can identify genes and pathways linked to the phenotype of interest that were not discovered through standard analyses of transcriptomic data. The flexibility of *scShapes* is also demonstrated through use cases that have been applied to scRNA-seq datasets that feature distinct experimental designs and experimental models.

## Data Description

A collection of 3 publicly available scRNA-seq datasets was used in this study. These datasets were downloaded from NCBI GEO (accession numbers and data descriptions given under “Datasets used”). For all the datasets, cell-type identification has been performed by their respective original publications, which we have used in the *scShapes* framework as prior biological knowledge. For each dataset, genes with nonzero expression in at least 10% of all cells within a treatment condition are retained and were used for all subsequent analyses.

## Analyses

### An overview of the *scShapes* framework

The entry point to the *scShapes* pipeline is a set of aligned read counts from a scRNA-seq experiment. For each treatment condition, we model a gene independently using the error distributions from all 4 possible distributions, Poisson, negative binomial (NB), zero-inflated Poisson (ZIP), and zero-inflated negative binomial (ZINB), with a log link function. A model-based normalization is applied to the read counts to account for differences in sequencing depth between libraries. This is done by including the log_10_ of the total unique molecular identifier (UMI) counts assigned per cell as an offset in the GLM model so that differences in sequencing depth are directly adjusted for. Furthermore, additional covariates can be incorporated in the GLM framework to account for any biases introduced by biological replicates or technical attributes of the experimental design like batch effects. The most appropriate model is first selected based on the Bayesian information criterion (BIC) and LRT statistic, to ensure the best distribution is selected for each gene (see Fig.   1 and Methods for more details).

### The overabundance of zeros in scRNA-seq data is gene specific

**Figure 2:**
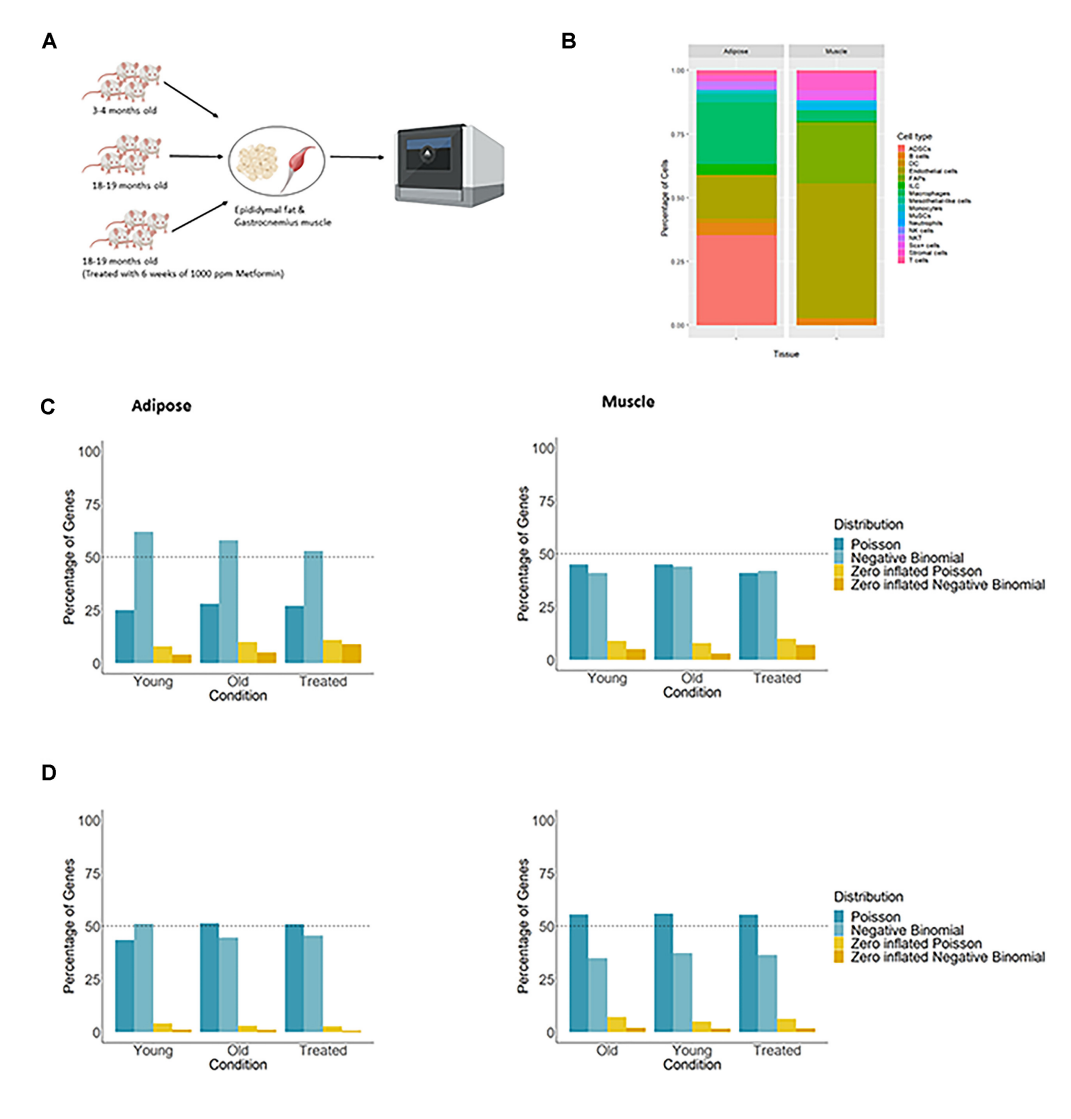
(A) Overview of the study design for the single-cell RNA-sequencing dataset using adipose and skeletal muscle in aging and metformin treatment in mice. Single cells have been isolated from epididymal fat and gastrocnemius muscle of 4 male C57BL/6 J mice in each of the 3 groups: old (18–19 months), young (3–4 months), and metformin treated for 6 weeks (18–19 months). Single-cell library preparation was done using the 10X Chromium single cell 3′ v2 kit, with quality control and gene expression quantification carried out in Cellranger. (B) Cell type abundances of the single-cell RNA-seq data in adipose and skeletal muscle. Seurat R package has been used for clustering and differential expression. Cells have been annotated manually by inspecting the gene expression profiles of marker genes identified using *FindAllMarkers* in Seurat for each cluster and validated using self-built Garnett cell-type classifiers. The cell-type membership was used as an explanatory covariate in the GLM framework of *scShapes*, to determine whether using prior biological knowledge would change the composition of genes following 1 of the 4 distributions. Bar plots for the percentage of genes following each distribution; (C) only accounting for technical variability (i.e., after adjusting for differences in sequencing depth and mouse ID); (D) accounting for both technical variability and known sources of biological variability (i.e., also adding cell type membership as a covariate in the GLM).

To demonstrate the *scShapes* differential distribution framework, we apply this to a scRNA-seq dataset collected for an aging study on 2 tissues, adipose and muscle, from mice (Fig. 2A). In the study design, there are 3 groups of mice that we designate as Young, Old, and Treated, where each group consists of 4 male mice as biological replicates (see Methods). GLM models with error distributions from the Poisson, NB, ZIP, and ZINB distributions are fitted without assuming any prior biological knowledge and correcting only for the technical variability between cells. This was done by including an offset in the GLM model to account for differences in sequencing depth and the mouse ID included as an explanatory covariate in the GLM to account for any biases introduced due to 1 mouse being potentially different from any of its other biological replicates.

We found that most genes (between 52% and 62% in adipose and between 40% and 45% in muscle) follow an NB distribution in both tissue types (Fig. 2C). In the muscle tissue, it is worth highlighting that at least 50% of the genes do not follow the NB distribution under the 3 treatment conditions. Collectively, over 55% of genes follow either the NB, ZIP, or ZINB distribution, and this observation aligns with the nature of scRNA-seq data, which is driven by overdispersion and zero inflation. These results provide evidence that in fact, not all genes have expression profiles that follow an NB distribution and, more importantly, demonstrate that not all the genes in the transcriptome follow a single distribution either.

### The inclusion of prior biological knowledge, like cell-type membership, changes the shape of a gene's expression distribution

We investigated whether accounting for known biological knowledge such as the membership of a specific cell type affects the classification of genes into their distribution shape. The aging mouse dataset consists of 13 known cell types in adipose and 12 known cell types in muscle (Fig. 2B). To account for the biological variation in the data, we introduced the cell-type information as an explanatory covariate in the GLM model. Accounting for cell-type membership resulted in a marked reduction in the number of NB-distributed genes, a resulting increase in Poisson-distributed genes, and a decrease in the number of zero-inflated genes (Fig. 2D and Supplementary Table S1). This observation is in line with Choi et al. [18], who also observed that adjusting for cell type resulted in a reduction of the number of zero-inflated genes.

**Figure 3:**
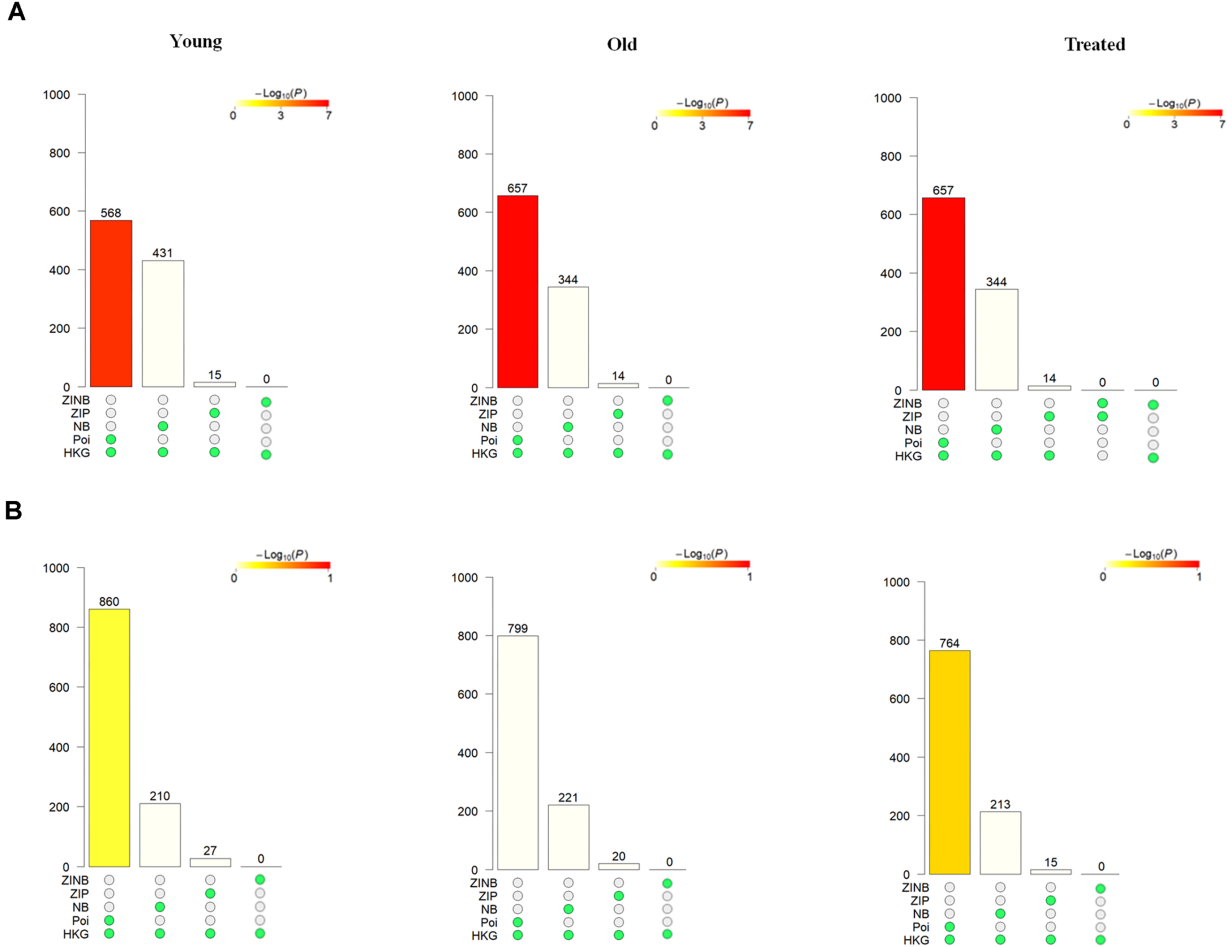
Investigating the prevalence of housekeeping genes (HKG) among genes that remain Poisson (P), NB, ZIP, and ZINB both with models correcting for technical variability and when accounting for known biological variability in (A) adipose and (B) muscle. *scShapes* modeling framework is first applied to identify the distribution shape in old, young, and metformin-treated group only accounting for the technical variability in the GLM model (i.e., only including the offset term and mouse ID as a covariate in the GLM to account for differences in sequencing depth and biological replicates, respectively). The same process is repeated again, including information on the cell types as a covariate in the GLM (in addition to the offset and mouse ID) to account for known sources of biological variability. We next checked for the overlap of genes that followed the same distribution in both instances above and checked for the statistical significance of the overlaps using the R package *SuperExactTest*. The height of each bar corresponds to the number of overlapping genes, and the color corresponds to the log_10_(*P*-adjusted) value that assesses the significance of the overlap.

Housekeeping genes are required for the maintenance of basic cellular functions and hence are usually uniformly expressed in all cells with low variance. Interestingly, we found that around 50% of the genes under each condition that remained Poisson distributed even after accounting for cell type membership were previously described as having a role as a housekeeping gene [19] (Fig. 3). The overlap between the Poisson-distributed genes with and without accounting for known cell types was significantly different only in adipose (adjusted *P-value* < 0.01, Fig. 3A). A gene that remains Poisson distributed regardless of whether cell-type specific expression rates were taken into account are indicative of housekeeping genes as the rate of expression in these genes does not vary across cell types. Our finding indicates that a Poisson error model is sufficient to explain the variability in the expression of genes with a housekeeping function.

### A simulation study to evaluate the sensitivity of the *scShapes* gene expression distribution classification

**Figure 4:**
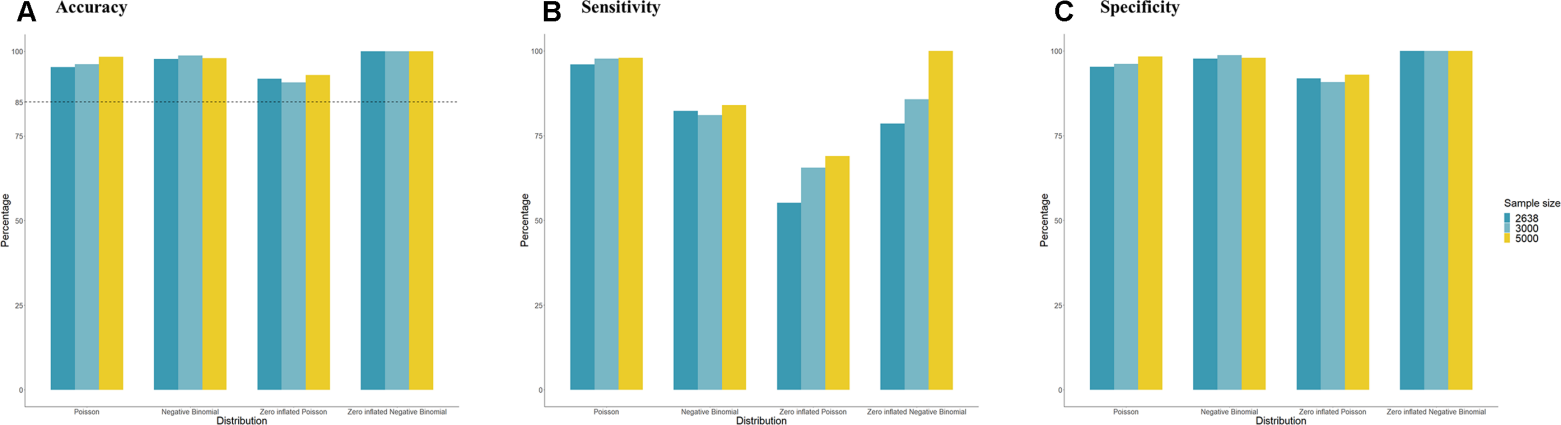
Summary statistics for the simulation study carried out to assess the performance of *scShapes* at identifying distributions of single-cell gene expression data. Summary statistics: (A) accuracy, (B) sensitivity, and (C) specificity of the simulation study conducted to evaluate *scShapes*’s ability to classify genes into a P, NB, ZIP, and ZINB distribution. Model parameters of the 4 distributions were estimated using the 3k PBMC dataset and were used for simulating gene expression values from P, NB, ZIP, and ZINB distributions using 3 sample sizes *n* (i.e., number of single cells). Next, *scShapes* was run on the simulated gene expression counts to identify the distribution of the simulated data. Results of the simulation study were evaluated using the 3 summary statistics of accuracy, sensitivity, and specificity.

We designed a simulation study to evaluate our framework's ability to accurately classify genes into Poisson, NB, ZIP, and ZINB distributions. To derive a realistic set of model parameters, we first performed model classification using the *scShapes* framework for the well-known 3k-cell peripheral blood mononuclear cell (PBMC) dataset. The PBMCs were downloaded from the 10X Genomics website [20]. Using the model classification and parameter estimation, we simulated count data for 3 sample sizes, 2,638, 3,000, and 5,000 cells (see Methods section). As expected, we see that as the sample size increases, the ability of our framework to correctly identify the best-fit distribution increases (Fig. 4). However, the more striking result is that our framework can accurately identify the correct model distribution for each gene with an accuracy of 85% or above for all 4 distributions across the range of sample sizes tested (Fig. 4A).

### The zero-inflation parameter can be interpreted as an estimate of biological zeros

Being able to distinguish the zeros that reflect technical dropouts versus genuine biological zeros is helpful for understanding how genes control cellular phenotypes. The zero-inflation parameter ${\hat{\pi }_0}$ in the *scShapes* model gives an estimate of the proportion of structural zeros for each gene that may be indicative of the subset of cells where the transcript is truly absent. These models make the assumption that the zero observations have 2 different origins—namely, structural zeros assumed to be observed due to specific structure in the data that are distinct from random zeros which are assumed to be observed due to sampling variability. In order to evaluate the relationship between the estimated zero-inflation parameter ${\hat{\pi }_0}$ and the percentage of zeros across all cells in genes detected to be zero-inflated (i.e., genes following either a ZIP or ZINB distribution), we plotted the estimated zero-inflation parameter against the percentage of zeros across cells for each zero-inflated gene (Supplementary Fig. S1). While most features have at least 50% or more zeros across cells, the zero-inflation parameter can vary between any value between 0 and 1. This indicates that there is either no relationship or a very weak relationship between the zero-inflation parameter and the percentage of zeros. It demonstrates that genes with a high percentage of zeros overall are not necessarily captured by models where the zero-inflation parameter is high. Choi et al. [18] have shown that the primary cause of zero inflation is due to biological variation and not only due to technical variation. So, one can assume that the excessive zeros we observe in single-cell data are the result of the 2 zero-generating processes, where either a transcript is absent from a biological system due to sampling variability or where a transcript is truly absent from the biological system. Hence, modeling genes with excess zeros using zero-inflated distributions will be ideal in such a scenario.

We also leveraged the zero-inflation parameter to identify genes that had an excessively high number of structural zeros. For example, around 98% of the zeros in the gene *Arhgap20* were structural zeros in adipose under the Old condition. This gene encodes a protein that is an activator of RHO-type GTPases. It has been shown to be associated with Alzheimer's disease and predicted to be a dysregulated gene [21]. Similarly, the gene *Sfxn1*, which has around 85% of structural zeros based on the estimate of the zero-inflation parameter in muscle under the Old condition, is found to be downregulated in senescence (SeneQuest), a process that is a causal factor for aging tissues [22]. *Sfxn1* is a protein-coding gene that mediates the transportation of serine to the mitochondria. Although the mitochondrial biology of the sideroflexin (SFXN) family is not entirely explored, dysfunction in mitochondrial carriers results in perturbations in oxidative phosphorylation, which underlies various pathologies, including aging-related diseases [23]. Hence, it could be hypothesized that the higher number of structural zeros in *Sfxn1* could be as a result of aging-associated mitochondrial dysfunction with aging in the muscle. These results highlight the possibility that the estimates of the zero-inflation parameter might be indicative of features that are truly absent from a biological system and therefore play a role in the regulation of the cellular phenotype under study.

### Around 30% of the genes undergo change in shape of distribution with aging or treatment, and collectively these differentially distributed genes demonstrate a significant enrichment of aging-related pathways

**Figure 5:**
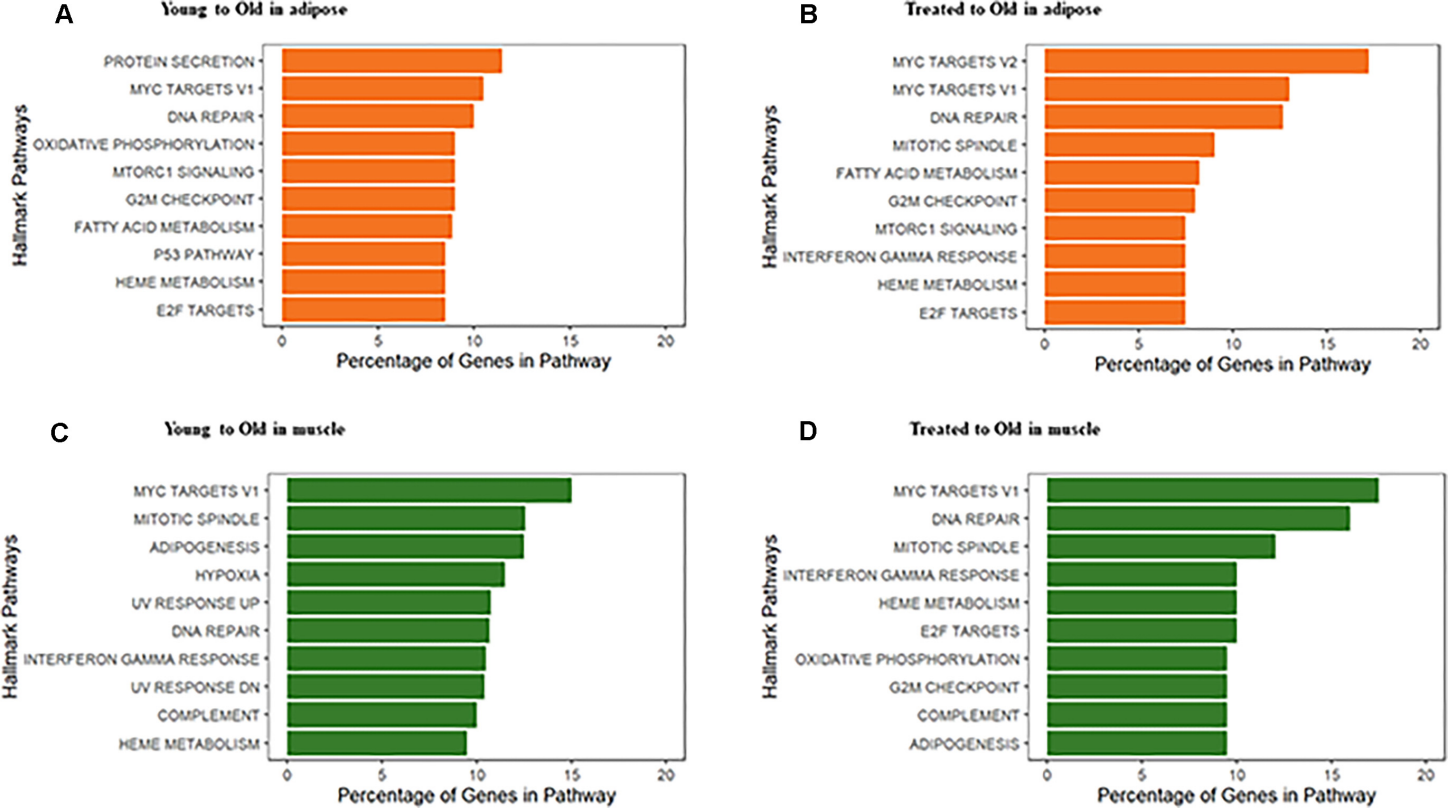
Pathway overrepresentation analysis of differentially distributed genes detected by *scShapes*. The top 10 significant pathways (BH-adjusted *P* < $1\ \times {10^{ - 4}}$) in the pairwise comparisons: (A) Young to Old in adipose, (B) Treated to Old in adipose, (C) Young to Old in muscle, and (D) Treated to Old in muscle using Hallmark pathways. The length of the bar corresponds to the percentage of differentially distributed genes that were represented in the Hallmark gene set.

Since we observed a considerable change in the composition of unimodal genes to zero-inflated genes after accounting for known cell types, this suggests that accounting for cell-type specific effects is important for addressing biological information about distributional shapes. To investigate the biological inference from enriched pathways of differentially distributed genes, we performed pathway overrepresentation analysis on the model distribution classifications obtained after taking into account cell-type specific gene expression for the mouse dataset by adjusting for cell type as a covariate. We identified the genes that switched their distributions either between Old and Young or Old and Treated. Nearly 30% of the genes (after filtering, i.e., of the genes with at least 10% expression across all cells within a treatment condition) were differentially distributed in at least one of the comparisons (Old vs. Young or Old vs. Treated) in both adipose and muscle. The top 10 Hallmark pathways and KEGG pathways most overrepresented among the genes switching distributions are represented in Fig. 5 and Supplementary Fig. S2, respectively.

The detection of statistically significant pathways provides evidence that the differentially distributed genes detected by our *scShapes* framework are enriched for biological processes related to aging. Consideration of what these processes are helps to illustrate how these genes may be participating in processes that are altered through aging. For example, in the Hallmark pathways, one of the most commonly enriched, most significant pathways includes *DNA damage*, where excessive DNA damage or poor DNA repair contributes to the aging process [24]. Among the most enriched KEGG pathways in the Treated group in adipose is the *insulin signaling pathway*, which is one of the primary nutrient-sensing pathways targeted by metformin [25]. Furthermore, the *MAPK signaling pathway* is involved in the regulation of differentiation, cell growth, proliferation, and apoptosis [26] and is enriched in both adipose and muscle in the treated cells. Metformin is found to be relevant in activating the MAPK signaling pathway and increases the expression of DNA damage and growth inhibition gene *GADD153* [27]. Among the differentially distributed genes, we find the transcription factors (TFs) *FOXO3* involved in regulating aging-associated stress response and proposed to be mediated by metformin [28, 29], *RXRA*, overexpression of which reduces DNA damage accumulation, leading to delays in replicative senescence [30] and previously shown to be targeted by metformin in human adipose [31], to be common in both Old vs. Young and Old vs. Treated comparisons in adipose. We then further investigated some of the differentially distributed genes identified by *scShapes*. For example, we observed that the gene *Foxo3*, followed a NB distribution in the Old group and a Poisson distribution in the Young group. Similarly, the same gene switched between a NB and Poisson distribution for the Old vs Treated comparison, respectively. This result indicates that during aging, we observe higher overdispersion in the expression of Foxo3 than that could be modeled with a Poisson distribution. Hence, it could be hypothesized that there are increased levels of gene expression heterogeneity observed during aging compared to the young phenotype, where treatment with metformin reverts the expression of Foxo3 with lesser overdispersion to levels comparable with the young phenotype. This observation could have been missed if we only focused on differential expression (DE) genes, as Foxo3 was not observed to be differentially expressed in either comparison. Similarly, some of the TFs commonly differentially distributed in muscle in both Old vs. Young and Old vs. Treated include *SRF*, reduction of which leads to premature aging in skeletal muscle, [32] and *IRF3*, a novel inhibitor of cellular senescence and inducer of cell growth inhibition [33].

### Genes switching to zero-inflated distributions in the Old condition may point to those genes involved in the regulation of transcriptional bursting

According to Clivio et al. [34], zero-inflated genes may reflect other biological phenomena such as transcriptional bursting. We investigated this hypothesis in the context of aging using the metformin-treated dataset. With age, a loss of transcriptional regulation is accompanied by an increase in transcriptional noise [35]. As transcriptional bursting leads to increased levels of gene expression noise, we used *scShapes* to identify the set of genes that switched from a unimodal distribution in the Young group to a more heterogeneous pattern captured by the zero-inflated distribution in the Old group while also switching from a unimodal distribution to a zero-inflated one in the Treated to Old groups, respectively (see Supplementary Fig. S3). The rationale behind using the *scShapes* framework to detect this specific profile is that these genes become more heterogeneous in the presence of aging (Young to Old) as well as in response to metformin treatment (Treated to Old). Interestingly, the top Hallmark-enriched term for genes switching to zero-inflated distributions in Old from both Young and Treated in muscle is *WNT beta catenin signaling* (Supplementary Fig. S3), a signaling pathway that may play a role in modulating gene expression noise [36]. Some of these genes include *HDAC5*, histone deacetylase, which plays an important role in transcriptional regulation and is involved in the chromatin dynamics of generating gene-specific noise [37]. These results draw our attention to the possibility that zero-inflated genes might be indicative of basic biological phenomena like transcriptional bursting. The use of the *scShapes* framework represents a methodological approach to detect this kind of unimodal to zero-inflated differential switching that may be useful for identifying further genes involved in the transcriptional bursting process. It is worth emphasizing that the flexibility of the *scShapes* framework and its range of distribution assumptions are what led to the identification of these genes, which would not have been necessarily detected by methods where an NB distribution is applied or assumes the prevalence of a single distribution shape for all genes.

### Comparison of *scShapes* with existing tools for differential gene expression analysis

To compare the performance of our *scShapes* framework for discovering age-related gene expression to that of standard DE, we carried out standard DE using *edgeR* [38], DESeq2 [39], and DEsingle [40], which utilizes a ZINB model for DE testing, and also compared our framework with *scDD* [12], which models the change in mean expression levels by comparing gene expression distributions. *edgeR* was used to test for differential expression, and the pairwise comparisons tested were between the Young vs. Old and Old vs. Treated groups. We applied *edgeR*’s quasi-likelihood approach (QLF) with cellular detection rate as a covariate, since this method was shown to perform well for DE analysis of scRNAseq data after filtering out lowly expressed genes [41]. Similarly, *DESeq2* was used with published recommendations for single-cell datasets, where an LRT was performed between the pairwise comparisons of interest to identify the differentially expressed genes. Genes were corrected using the Benjamini and Hochberg (BH) correction, and only genes that had a corrected *P*value < 0.01 were retained. Although there were similar number of DE genes between *scShapes* versus *edgeR*,*DESeq2*,*DEsingle*, and *scDD* (Table 1A), there is little overlap even across existing methods (Table 1B). The genes found by our *scShapes* method that are exclusively switching between a unimodal distribution and a zero-inflated distribution represent over 25% of the genes in adipose and over 45% of the genes in muscle. Some of the unique genes detected by our pipeline include the TFs *FOXO3* and*PKNOX1*, involved in apoptosis, regulation of oxidative stress, and DNA damage [42] in adipose. Similarly, the TFs *IRF3* and*TFE3*, involved in autophagy, are some of the uniquely detected genes by our pipeline in muscle.

**Table 1:** Comparison of genes identified by *scShapes* with 4 other methods, *edgeR*, *DESeq2*, *DEsingle*, and *scDD*. The total number of genes for *DEsingle* includes genes detected using the categories DEs, DEa, and DEg. Similarly, the total number of genes for *scDD* includes genes detected using the categories DE, DP, DB, and DZ.

**(A) No. of genes detected to be either DE or DD by each method**						
Tissue	Comparison	*scShapes*	*scDD*	*edgeR*	*DESeq2*	*DEsingle*
Adipose	Old vs. Young	902	2,291	2,045	1,849	2,104
	Old vs. Treated	825	1,214	916	438	891
Muscle	Old vs. Young	988	338	519	424	457
	Old vs. Treated	986	319	393	379	450

**(B) Comparison of detected genes between methods**				
Tissue	Comparison	Overlap between all methods	Overlap between published methods	Overlap between DE methods
Adipose	Old vs. Young	28	359	435
	Old vs. Treated	1	25	30
Muscle	Old vs. Young	4	21	29
	Old vs. Treated	0	13	20

Published methods include *edgeR*, *DESeq2*, *DEsingle*, and scDD. DE methods include *edgeR*, *DESeq2*, and *DEsingle*.

Of the genes that were detected as differentially distributed by our *scShapes* method, between 25% and 27% of the genes switched distributions from a unimodal to a zero-inflated distribution in adipose (Supplementary Table S2A). However, in muscle, around 50% of the differentially distributed genes switched distribution from a unimodal to a zero-inflated distribution. The genes switching distribution from a unimodal to a zero-inflated distribution are indicative of the genes that have excessive zeros in one condition compared to another condition. We further investigated the patterns of differential distributions to identify what percentages of genes switched distribution from NB to either , ZIP, or ZINB (or vice versa), and this information is summarized in Supplementary Table S2B. It can be seen that around 50% to 75% of the genes switched distribution between an NB to Poisson distribution (or vice versa) in both pairwise comparisons for both tissues.

We next looked at what proportion of these genes were also detected as differentially expressed by *edgeR*, *DESeq2*, or *DEsingle*. Although there was not a high overlap between the differentially distributed genes detected by our pipeline and the differentially expressed genes detected by the other methods, we observed that most of the genes commonly detected by *scShapes* and *edgeR* or *DESeq2* switched distribution from NB to Poisson, ZIP, or ZINB (or vice versa; Supplementary Table S3A,B). Further investigation of the overlap of genes detected by *edgeR* or *DESeq2* and our *scShapes* framework revealed that most of these genes followed a unimodal distribution in the 2 pairwise comparisons in both adipose and muscle (48% to 93% of the genes; see Supplementary Table S4A,B). This is to be expected since both *edgeR* and *DESeq2* detect differential expression by assuming that the gene expression levels follow a negative binomial distribution for all genes. Similarly, one would expect that genes detected to be both differentially distributed by *scShapes* and differentially expressed by *DEsingle* would follow a ZINB distribution. However, only in muscle tissue between old and treated did we observe around 56% of the differentially distributed genes to switch distribution from ZINB to Poisson, NB, or ZIP (or vice versa; Supplementary Table S3C). In all other comparisons, only less than 50% of the genes that were both differentially distributed and differentially expressed switched distribution from ZINB to Poisson, NB, or ZIP (or vice versa; Supplementary Tables S3C and S4C).

Similarly, we also compared the distribution patterns of commonly differentially distributed genes that were detected by our DD pipeline and that of *scDD*. Compared to *edgeR*, there is little overlap between the genes detected by our pipeline and that of *scDD* in muscle, although this number is quite similar for adipose (33% and 15% in Old vs. Young and Old vs. Treated, respectively; see Table 2). Although both approaches are designed to detect differential distributions, they each make very different modeling assumptions about the distributions. In *scDD*, log-normalized gene expression measurements are modeled using a Bayesian nonparametric modeling framework utilizing Dirichlet process mixture models. The different assumptions that are made in *scDD* and *scShapes* mean that these methods are not equally affected by the degree of heterogeneity occurring from cell to cell in the adipose and muscle tissues, and hence different sets of genes were obtained. Nevertheless, we further investigated the genes identified by both *scShapes* and *scDD* to identify functional terms enriched in the overlapping gene sets. We observed that Hallmark pathways such as *DNA repair* and *MTORC1 signaling* were enriched in adipose between both pairwise comparisons. Similarly, in muscle, we observed that the Hallmark pathways such as *adipogenesis* and *hypoxia* are overrepresented in genes identified to be differentially distributed by both *scShapes* and *scDD* between old and young (Supplementary Fig. S4).

**Table 2:** Investigating whether genes detected by *scShapes* share any similarities with distribution categories detected by *scDD*

Tissue	Comparison	Overlap between DD genes from *scShapes* and *scDD*	Genes detected as DB by *scDD*	Genes detected as DE by *scDD*	Genes detected as DP by *scDD*	Genes detected as DZ by *scDD*
Adipose	Old vs. Young	301	3	177	0	121
	Old vs. Treated	112	2	47	0	63
Muscle	Old vs. Young	51	0	12	0	39
	Old vs. Treated	20	0	9	0	11

Of the genes that were commonly detected by *scShapes* and *scDD*, most of them were categorized either as DE or DZ (differential proportion of zeros) by *scDD*. For the genes that are not differentially distributed in the nonzero values, *scDD* checks whether the proportion of zeros is significantly different between the 2 conditions (DZ). We further investigated the pattern of distribution of the genes detected to be DD by our pipeline as well as classified as DZ by *scDD* (Supplementary Table S5). We checked whether these genes switched from a unimodal distribution to a zero-inflated distribution between conditions. However, less than 50% of the genes would switch distribution between a unimodal distribution and a zero-inflated distribution in the genes classified as DZ by *scDD*. This suggests that *scShapes* is detecting a different type of profile change in gene expression than what can be explained by *scDD*’s DZ criteria alone.

### Additional case studies to demonstrate *scShapes*’ utility for a wide range of experimental designs

The utility of the *scShapes* framework can be best demonstrated by applying it in the context of multiple datasets that represent a diverse range of biological perturbations. The primary dataset used in this study describes one of the most common experimental designs used for scRNA-seq approaches where single cells are profiled from multiple treatment conditions (e.g., young, old, and treated mice). This design is illuminating for understanding aging and metformin-induced effects on the transcriptome. It is important to recognize that other kinds of designs are represented in scRNA-seq datasets and that *scShapes* is able to infer insightful results for these too. We selected 2 other experimental designs to showcase the flexibility and utility of *scShapes* to identify useful biological results in the presence of different degrees of complexity present in scRNA-seq data.

### Using *scShapes* to gain insights into the transcriptional response of the immune system in COVID-19 patients

A scRNA-seq dataset was generated for PBMCs from 7 patients hospitalized for COVID-19 and 6 healthy controls [43] (Supplementary Fig. S5A). We selected this dataset to showcase how *scShapes* can detect differential distributions in the presence of a much higher number of cells (∼44,721 cells vs. ∼12,987 in the previous aging dataset) and make use of multiple covariates. In the original study, Wilk et al. [43] classified each cell in the COVID-19 patient data and healthy controls into 1 of 20 known cell types. Cell-type membership, donor ID, and gender were included in *scShapes* as explanatory covariates in the GLM. *scShapes* was used to determine the prevalence of genes classified into each of the 4 distributions of Poisson, NB, ZIP, and ZINB (Supplementary Table S6), as well as determine genes that switched distributions between the COVID-19 group and the healthy control group.

Interestingly, we found that over 80% of genes followed an NB distribution, with the number of genes Poisson distributed being negligible. Pathway overrepresentation analysis of the genes switching distribution between the COVID-19 group and healthy controls revealed pathways like *oxidative phosphorylation*, *interferon response*, and *immune response* (Supplementary Fig. S5B–D), which are all pathways previously implicated with COVID-19 [44, 45].

We also used this dataset to investigate the nature of differential distributions occurring within a specific cell type. We focused our attention on the 7 main cell types: B cells, natural killer (NK) cells, dendritic cells, CD4^+^ T cells, CD8^+^ T cells, CD14^+^ monocytes, and CD16^+^ monocytes. We found that there was significant overlap between the differentially distributed genes in NK cells and the T cells (Supplementary Fig. S6A). We also found that differentially distributed genes were enriched for interferon responses, inflammatory responses, and NF-κB signaling pathway (Supplementary Fig. S6B). We also saw a statistically significant higher enrichment of *apoptosis*, metabolic pathway *oxidative phosphorylation*, and *WNT/β-catenin signaling* between the differentially distributed genes between the COVID-19 group and healthy controls in NK cells, CD4^+^ T cells, and CD8^+^ T cells.

### Pathway overrepresentation analysis of simulated T cells highlights pathways implicated in T-cell activation

In contrast to the previous designs, we included a design that featured a single cell type that was profiled in different tissues. This kind of experimental design is relevant to scRNA-seq datasets because we have already shown that overdispersion and zero inflation observed in gene expression rates are influenced by the presence of different cell types. A study generated scRNA-sequencing data of human T cells isolated from bone marrow (BM), lungs (LG), and lymph node (LN) from 2 adult deceased organ donors and blood (BL) from 2 healthy donors for comparison [46] (Supplementary Fig. S7A). Tissues acquired from the donors were stimulated, resulting in over 50,000 resting and activated T cells. We used *scShapes* to model the gene expression distributions separately under the 2 treatment conditions, resting (nonstimulated) and activated (stimulated), where the donor ID was used as an explanatory covariate that was adjusted for in the GLM model. As expected, the Poisson distribution was the most prevalent distribution across the 4 tissue types in both treatment conditions. Interestingly, we see that there were more genes with distributions that were classified as NB, ZIP, or ZINB distributed across stimulated T cells in all tissue types and blood (see Supplementary Table S7) compared to resting T cells. The genes that switched distributions between resting and activated (Supplementary Table S8) in the 4 tissue types were enriched for *activation of immune response* and other Gene Ontology (GO) terms (see Supplementary Fig. S7B–E). This result likely reflects the fact that in BM, LG, and LN, the activated T cells secrete cytokines that regulate immune response. The differentially distributed genes in this pathway that are shared between BM, LG, and LN include *PSMC1* and *PSMC3*, where both of these genes are part of the 26S proteasome involved in cell-cycle progression, DNA damage repair, and apoptosis, and *XRCC6* is involved in regulation of innate immune response [47].

### Benchmark of *scShapes* computational needs

As *scShapes* estimates the parameters of each gene by maximum likelihood estimation (MLE) using the iterative weighted least squares (IWLS) algorithm, the computational cost of the framework is high. In order to get an evaluation of the time taken to run *scShapes* as a function of the number of cells, we utilized the Human PBMC dataset from [48]. This dataset comprised 161,764 cells collected from 3 time points collected at days 0, 3, and 7 from 8 volunteers. We ran *scShapes* on each time point, with volunteer ID and cell-type membership included as covariates in the GLM. The modeling and testing framework of *scShapes* can be summarized under 3 main steps: (i) the Kolmogorov–Smirnov (KS) test to identify the genes belonging to the family of ZINB distributions; (ii) model fitting using the 4 distributions of Poisson, NB, ZIP, and ZINB; and (iii) goodness-of-fit tests to identify the best model for each gene. Hence, the computational needs of *scShapes* were also benchmarked under the above 3 main categories. It is evident that the computational demand, especially for running the KS, test is nonnegligible. This is mainly due to fitting ZINB distributions by estimating the unknown parameters from sample estimates with *P*values calculated from bootstrapped Monte Carlo estimates, both of which are computationally expensive.

**Figure 6:**
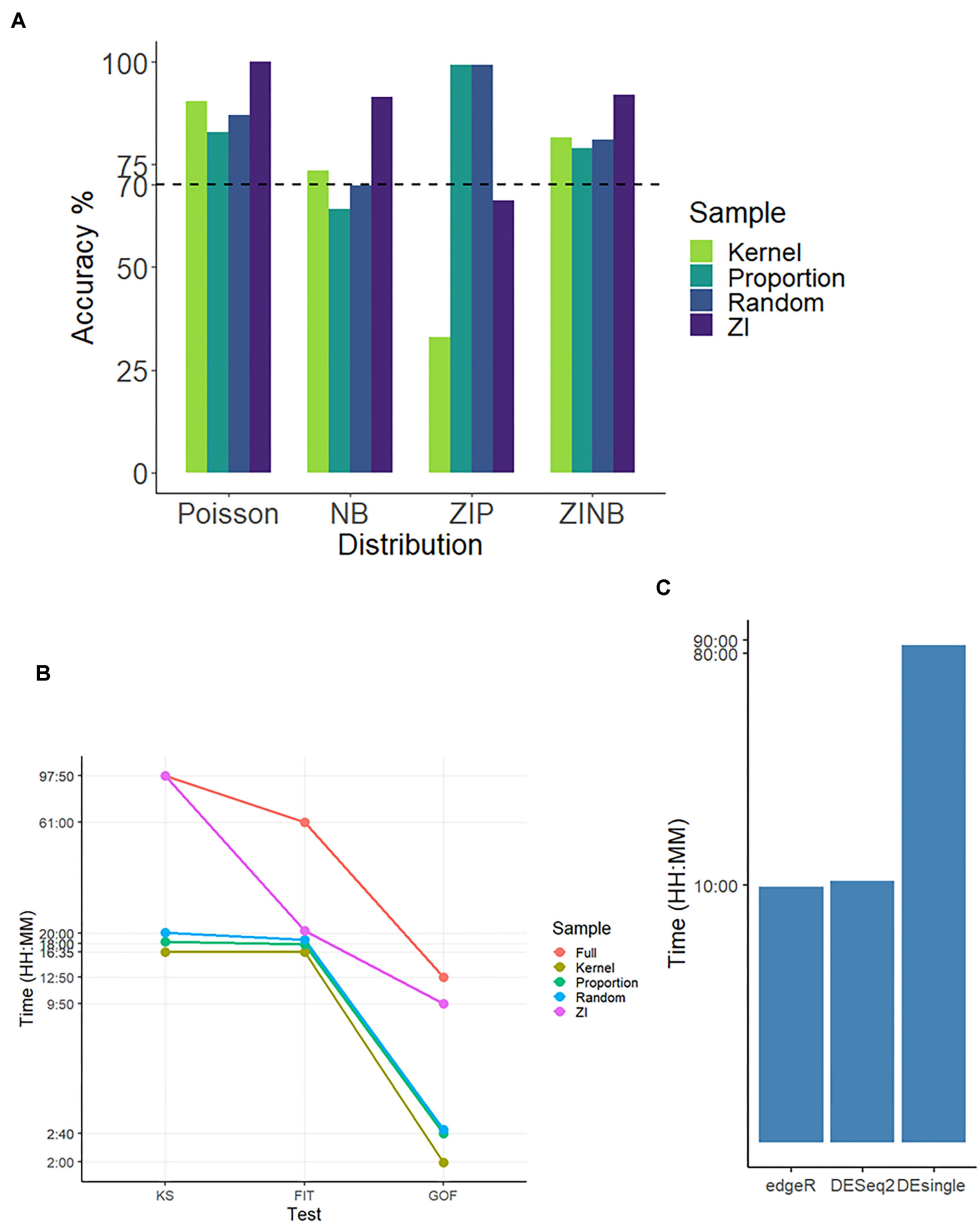
*scShapes* computational needs. The computational needs and accuracy of *scShapes* were benchmarked using PBMCs collected from 8 volunteers at days 0, 2, and 7. The dataset comprised ∼162,000 cells. *scShapes* was run on each time point separately, and the results of each time point were averaged together for the benchmarking purpose. This resulted in an average of ∼54,000 cells in the full dataset and ∼8,000 cells in the downsampled dataset. The following 4 strategies were followed: (i) randomly sampling 1,000 cells from each cell type (Random), (ii) density-based sampling of 1,000 cells from each cell type (Kernel), (iii) randomly sampling cells totaling 8,000 cells while maintaining the original cell proportions (Proportion), and (iv) introducing an additional test for zero inflation (ZI). *scShapes* was run each condition separately (dau 0, 2, 7). (A) Bar plots summarizing the average accuracy of the 4 methods in identifying the correct distribution. (B) Average computational times required to run *scShapes* on the full dataset and by each of the 4 methods (Random, Kernel, Proportion, and ZI). The modeling and testing framework of *scShapes* was summarized under 3 main steps: (i) KS test to identify the genes belonging to the family of ZINB distributions; (ii) model fitting using the 4 distributions of Poisson, NB, ZIP, and ZINB (FIT); and (iii) goodness-of-fit tests to identify the best model for each gene (GOF). To benchmark the computational times of *scShapes* vs. existing methods, we ran *edgeR*, *DESeq2*, *DEsingle*, and *scDD* on the pairwise comparisons (on the full dataset) on day 0 vs. day 2 and day 0 vs. day 7 and computed the average time taken by each of the 4 methods. (C) Bar plot summarizing the average computational times taken by exiting DE/DD methods (computation of scDD aborted due to limitations in memory).

Hence, we tested whether prefiltering for zero inflation or by downsampling the cell numbers the computational cost could be reduced. For this purpose, we utilized a test to check for zero inflation in count models to test whether a Poisson model is under fitting zeros in the observed variable (see Methods). Then, the zero-inflated distributions (ZIP and ZINB) will only be fitted to the genes identified to be underfitted by a Poisson distribution. By introducing the test for zero inflation (ZI), the average computational time taken for fitting the 4 distributions reduced by around 67% (Fig. 6B). While the distributions of Poisson, NB, and ZINB were identified with an accuracy of over 91% after introducing the zero-inflation test (Fig. 6A), there was a drop in accuracy when identifying the genes following a ZIP distribution (accuracy = 67%).

We next tested the impact of cell numbers and cell proportions in accurately identifying the distributions as well as on the computational demands. For this purpose, we followed 3 main strategies where we first randomly downsampled the total cells to 8,000 cells (Random), with each cell type having an equal number of cells each (1,000 cells each), and another iteration where we maintained the original cell proportions (Proportion). Finally, instead of randomly sampling cells, we subsampled cells in a density-dependent manner (Kernel) as previously done by [49] with 1,000 cells in each cell type. It is evident that by downsampling, the computational time has drastically reduced by around 77% compared to running *scShapes* on the entire dataset (Fig. 6B). Overall, we can see that each of the subsampling techniques achieved over 70% accuracy except the Proportion method when identifying the NB distribution (65% accuracy) and the Kernel method in identifying the ZIP distribution (Fig. 6A). However, it is noteworthy that of the total genes, less than 1% of the genes in the PBMC dataset were identified to be following a ZIP distribution.

Finally, we benchmarked the computational time required by *edgeR*, *DESeq2*, *DEsingle*, and *scDD* for identifying DE or DD genes in the Human PBMC dataset. The calculations of *scDD* aborted because it ran out of memory when performing the Fisher's exact test. Both *edgeR* and *DESeq2* were run by including the volunteer ID and cell-type membership as covariates in the GLM. However, *DEsingle* does not facilitate the inclusion of covariates in its modeling framework. It can be seen that only *DEsingle*, which models the count data using ZINB distributions, required around 90 hours on average to identify the DE genes (Fig. 6C). All computations were performed on a high-performance computing cluster with 250 GM of RAM and with parallel computing using 6 nodes (except in *edgeR*, which did not facilitate parallel computation).

## Discussion

We have presented a flexible statistical framework for quantifying gene expression heterogeneity of scRNA-sequencing data by modeling gene expression levels under a differential distribution pipeline. What makes our method unique is that it is fundamentally different from a method that detects differential expression by testing for the significance of change in the mean of the expression rates. Our framework identifies and classifies genes according to their shape of gene expression distribution and allows for the possibility that one gene's expression profile across a population of single cells may be different from another gene. As scRNA-seq data are characterized by properties of sparsity and high dimensionality, our framework uses a special class of statistical distributions that can handle an excess degree of zeros. Recently, there has been discussion in the literature regarding the use of zero-inflated distributions for modeling single-cell transcriptomic data. It is argued in Cao et al. [50] that zero inflation is suppressed in gene expression counts measured in terms of UMI counts and in Svensson [51] that an NB distribution alone is sufficient to model the zero counts in droplet-based scRNA-sequencing data. However, this study [51] models the gene expression heterogeneity of negative control (e.g., External RNA Controls Consortium spike-ins) droplet-based scRNA-seq data. As negative control data lack biological variation, an NB distribution may sufficiently model such gene expression measurements and hence there remains motivation to model the excess zeros of at least a subset of the genes. Although the focus of this study has been based on scRNA-sequencing data generated through droplet-based methods, we show that zero inflation is required for a significant number of genes to ensure the best distribution fit depending on the nature of the experiment, because around 5% to 20% of the genes under different experimental conditions were detected to follow a zero-inflated distribution. Our study also validates the fact that zero inflation may be interpreted from a biological perspective (i.e., based on cell-type membership), which is in line with the findings of Choi et al. [18] and Clivio et al. [34], and not completely a result that is just an artifact of technical variability.

When compared to other popular tools for identifying differential distributions like *scDD* [12], it is valuable to acknowledge that the *scShapes* approach has the flexibility to account or adjust for multiple confounders in the form of covariates in the GLM. Our approach does not require that prior normalization be done for nuisance factors but rather enables model-based normalization using the GLM. Furthermore, our method also has the flexibility to perform multiple comparisons across biological conditions and is not restricted to only pairwise comparisons. This feature provides the option to investigate both global (e.g., effects treatment groups) and pairwise changes (e.g., young vs. old), similar to an analysis of variance test followed by a post hoc pairwise comparison but for assessing differential distributions.

One of the tools that exists for identifying the most appropriate statistical model is *M3S* [52], which selects the best-fit model from a pool of 11 models using a KS test. The 11 distributions considered by *M3S* include the 4 distributions included in our *scShapes* method and additional statistical distributions such as Gaussian mixture models. However, we believe that the use of Gaussian distributions to model count data is not advisable as we run the risk of losing information by fitting continuous distributions to count data. Additionally, the use of 11 different distributions makes it more challenging to biologically interpret the best-fit distribution for each gene. Hence, for simplicity and efficiency, the 4 distributions of Poisson, NB, ZIP, and ZINB represent a sufficient selection for modeling scRNA-seq data.

*scRATE* is another recently published tool for modeling droplet-based scRNA-seq data that uses a mix of unimodal and zero-inflated distributions [19]. This method relies on Bayesian model selection to identify the best model for each gene and is similar to our method as it also models gene expression distributions using Poisson, NB, ZIP, and ZINB distributions using a GLM with an offset term to account for differences in sequencing depth. Importantly, where this method differs from our approach is in its handling of model selection, which is based on the expected log predictive density score. Also, the main focus of this study was to identify the origins of zero inflation rather than to detect differentially distributed genes. Since the key objective of our approach is to detect differential distributions for the purposes of understanding biological perturbations, we ensure that the best-fit distribution is identified as appropriate to each gene from the pool of 4 distributions considered by first performing a KS test to identify genes belonging to the family of ZINB distributions. Only the genes that pass through the KS test would then be considered for the 4 distributions of Poisson, NB, ZIP, and ZINB to identify the distribution of best fit. Once model selection is done using the BIC value, we also perform additional model adequacy tests such as testing for the presence of zero inflation in the genes detected to be following a zero-inflated distribution to ensure that the model distribution is identified correctly and appropriately. The use of the KS test together with the model adequacy tests is crucial since our primary focus is identifying genes that switch distribution shape. While *scRATE* also employs a GLM-based modeling framework with Bayesian model selection, we developed our own modeling framework, *scShapes*, which utilizes MLE as Bayesian estimation is best suited for data with prior knowledge about the distribution it originated from. It is also noteworthy that both the tools *M3S* and *scRATE* fail to first subset genes that belong to the family of statistical distributions under study but rather assume that all the genes would follow one of the distributions from the pool of distributions considered. Our study, as well as others, presents compelling evidence that this assumption is limited or incorrect, especially for scRNA-seq data.

Compared to standard methods of DE, which aim to detect a shift in the mean of gene expression data such as *edgeR*, *DESeq2*, or *DEsingle*, our method does not make the assumption that all the genes in the transcriptome follow a single distribution. While these methods perform well at detecting statistically significant differences in mean between conditions, they fail to detect subtle changes in gene expression levels that do not involve a change in mean. In addition, even though the negative binomial distribution is a flexible distribution that is capable of handling overdispersion in the data, it lacks the flexibility to explicitly model both structural zeros and random zeros in the data. As shown by Choi et al. [18], the zeros we observe in scRNA-seq data are biological in nature and not purely produced by technical artifacts. However, a negative binomial distribution assumes that the zeros are a result of random sampling and does not allow for the recognition of true biological zeros in the data. This assumption that is made by the NB distribution goes against one of the core truths in biology and one of the key strengths of single-cell profiling techniques. Hence, it is vital that we model gene expression distributions at the gene level to allow for both random zeros and structural zeros in the data to be identified.

Our framework *scShapes* is built on the assumption that, once all sources of variation other than true biological variation are accounted for, the change in distribution we see is due to the phenotypic changes under consideration in the experimental study. Furthermore, our framework does not aim to cluster cells into subtypes as genes are modeled and evaluated independently. We acknowledge that while this framework is computationally expensive and can be time-consuming, especially when applied to datasets with large number of cells, it is possible that improvements through parallelization or more advanced usage of high-performance computer clusters could address this limitations. By downsampling from a larger PBMC dataset, we showed that *scShapes* was able to correctly detect the distribution with an overall accuracy of around 65% or more regardless of the sampling scheme or cell-type proportions, even with only around 15% (8,000/53,921 cells) of the total cells. This provides a promising opportunity to address the computational demands of *scShapes* in the future. Additionally, graphical processing unit (GPU) acceleration of the GLM algorithms provides a promising opportunity to parallelize the multiple manipulations of large single-cell data matrices and speed up calculations. Hence, this is a potential future extension for *scShapes* to improve the computational time by implementing GPU computing and infer differential distributions of thousands of genes simultaneously.

Some of the genes and pathways discovered through our framework overlap with those identified through methods of transcriptomic data where standard assumptions have been made, indicating the framework's ability to identify known markers. Importantly, this method is able to identify additional genes and pathways both at the tissue level and cell-specific level, which may point to new knowledge such as in the example of aging. Most important, the *scShapes* framework has the flexibility to adjust for any number of covariates (e.g., batch effects, cell types) and perform multiple comparisons across any set of biological conditions of interest. Hence, modeling a gene's expression distribution shape at the gene level provides an opportunity to extract precise genes and pathways not necessarily put forth by traditional methods of DE.

## Methods

### Code availability

We have implemented our model selection pipeline as an open-source R package, *scShapes*, available from Bioconductor for Linux, Windows, and MacOS. The source code is available on [53] under a GPL-3 license. Documentation on the functions and examples for *scShapes* is also available on our GitHub repository. Using *scShapes* requires an input of a matrix of UMI counts with genes as rows and cells as UMIs, along with a data frame of covariates to be included in the GLM and a numeric vector of library sizes. The library sizes are calculated as the total UMI counts per cell, which will be used as an offset in the GLM to account for differences in the sequencing depth. We recommend only a few genes be fitted on a local machine due to the computational demand. Hence, we have containerized the software using both docker and singularity to facilitate its use on high-performance computing clusters or with cloud computing. *scShapes* is listed on SciCrunch at RRID:SCR_022838 and on the bio.tools registry at biotools:scshapes. All analysis scripts used in the article can be accessed at [54].

### Methodology

In order to ensure that only expressed genes are included in the analysis, we retained genes expressed (defined as having a read count >0) in at least 10% of all cells under each treatment condition. We then restricted our attention to the subset of genes common between all 3 conditions for downstream analysis. The UMI counts for a given gene are modeled using GLMs under each condition separately. The sum of all counts in each cell is used as a proxy to normalize the cells for sequencing depth differences. This cell attribute is used as an offset in a regression model along with any other covariates to account for known sources of cell-to-cell variability. The 4 models being compared are the Poisson (P), NB, ZIP, and ZINB, which are special cases of one another, and all belong to the family of ZINB distributions. Hence, only the subset of genes belonging to the ZINB family of distributions is selected via a KS goodness-of-fit test, with *P*values computed through Monte Carlo simulation. For the significant genes from the KS test under each condition, the expression level of a given gene is then modeled using the error distributions of Poisson, NB, ZIP, and ZINB with a log link function.

### Parameter estimation

For model fitting and parameter estimation, the R package **pscl** [55], which allows for the fitting of zero-inflated models, was used. The 2 unimodal distributions, Poisson and NB, are implemented in R by the *glm* function [56] in the **stats** package and the *glm.nb* function [57] in the **MASS** package, respectively. The package **pscl** provides an extension over the existing R packages for fitting GLM, where the regression coefficients are estimated by MLE using the IWLS algorithm.

The parameter estimates are obtained by maximizing the likelihood or log-likelihood of the parameters for the observed data. The log-likelihood for the observation $y$ given the parameter $\theta $, expressed as a function of the mean value parameter, $\mu \ = \ E( Y )$, is given by

\begin{equation*} l\left( {\mu ;y} \right) = \log f\left( {y;\theta } \right) \end{equation*}

where $f( {y;\theta } )$ is the density function of $y$. The log-likelihood based on a set of independent observations ${y_1},\ \ldots ,\ {y_n}$ is the sum of the individual contributions, so that

\begin{equation*} l\left( {\mu ;y} \right) = {\mathrm{\ }}\mathop \sum \limits_i \log {f_i}\left( {{y_i};{\theta _i}} \right) \end{equation*}

where $\mu \ = \ {\mu _1},\ \ldots ,\ {\mu _n}$.

### Model selection

A critical step in the model-building process is the selection of the model that best fits the data among a set of competing models. Among numerous criteria available for model selection, we decided to select the best model based on the least BIC value as the BIC generally introduces a higher penalty for models with a more complicated parametrization, and BIC [58] values are calculated as follows:

\begin{equation*} {\mathrm{BIC\ }} = {\mathrm{\ }} - 2.\ln \left( {\hat{L}} \right) + k.\ln \left( n \right) \end{equation*}

where $k$ is the number of free parameters to be estimated, $\hat{L}$ is the maximum value of the likelihood of the model, and $n$ is the number of observations.

However, most applications in the literature use different diagnostics in conjunction with the information criterion to arrive at the model that best fits the data. Hence, further model adequacy tests were done to validate the models selected based on the least BIC value. The deviance goodness-of-fit test assesses the adequacy of the model by comparing the fitted model with the fitted saturated model. The deviance statistic $D$, also known as the likelihood ratio statistic, is defined as

\begin{equation*} D{\mathrm{\ }} = {\mathrm{\ }}2\left[ {{l_s}\left( {\hat{\psi }} \right){\mathrm{\ }} - l\left( {\hat{\beta }} \right)} \right]
\end{equation*}

where $\hat{\psi }$ and $\hat{\beta }$ are the MLEs of the saturated and fitted models, respectively. The deviance statistic is asymptotically chi-square distributed with $K - p$ degrees of freedom, where $K$ and $p$ are the number of parameters in the saturated and fitted models, respectively [59]. That is,

$D\ \sim\ \chi _{K - p}^2$

However, the deviance goodness-of-fit test cannot be used to test the adequacy of the zero-inflated models as these models are not strictly nested (i.e., the smaller model sits on the boundary of the parameter space of the larger model) [60]. Conversely, Molenberghs and Verbeke [17] showed that the resulting likelihood ratio (LR) test statistic of the form

\begin{equation*} \varrho {\mathrm{\ }} = {\mathrm{\ }} - 2{\mathrm{\ }}\left[ {l\left( {{{\hat{\beta }}^{\left( r \right)}},{\mathrm{\ }}{{\hat{\phi }}^{\left( r \right)}},0} \right) - l\left( {\left( {\hat{\beta },{\mathrm{\ }}\hat{\phi },\hat{\omega }} \right)} \right)} \right]
\end{equation*}

where $\omega $ is the zero-inflation parameter and $l( {{{\hat{\beta }}^{( r )}},\ {{\hat{\phi }}^{( r )}},0} )$ is the LR of the model with all parameters of the model estimated with the restriction $\omega \ = \ 0$ follows an equal mixture of a chi-square distribution with 0 degrees of freedom and a chi-square distribution with 1 degree of freedom [60]. That is,

\begin{equation*} \varrho {\mathrm{\ }}\sim{\mathrm{\ }}\left( {0.5} \right)\chi _0^2 + \left( {0.5} \right)\chi _1^2
\end{equation*}

Hence, we test for the presence of zero inflation in the zero-inflated models selected based on the least BIC value using the above test.

### Identifying differentially distributed genes

Each gene is modeled using a GLM where the read count ${y_{ij}}$ for gene $i$ in cell $j$ is assumed to follow one of the following distributions, namely, Poisson, NB, ZIP, or ZINB under each biological condition. After performing model adequacy tests detailed above, the distribution that best fits for each gene is identified. Once the statistical distribution for each gene is identified, comparisons in distribution shapes between biological conditions are carried out to identify genes switching distribution shape between treatment conditions.

#### Test for zero inflation

In order to speed up the modeling process of *scShapes*, we utilized an additional test for zero inflation. First, the GLM with Poisson error distribution is fitted for each gene (after filtering), and next it is tested whether this predicted model is underfitting the zeros compared to the observed zeros. For this purpose, we utilized the *check_zeroinflation* function from the performance (version 0.9.0) R package. Briefly, this test compares the ratio of observed and predicted zeros. A ratio between 1 ± tolerance ( = 0.05) is considered a well-fit model. Hence, only genes with a ratio above or below this threshold were subsequently fitted with the zero-inflated distributions.

### Datasets used

#### Metformin mouse dataset

The scRNA-seq metformin dataset was collected from mice on the 2 tissues, adipose and muscle. The study design had 3 groups of mice, with each group consisting of 4 male C57BL/6 J mice, with the Young group composed of 3- to 4-month-old mice, the Old group composed of 18- to 19-month-old mice, and the Treated group composed of 18- to 19-month-old mice treated with 1,000 ppm (0.1% w/w) metformin for 6 weeks [61]. Using scRNA-seq, the transcriptome of ∼13,000 cells from adipose stromal-vascular fraction and ∼5,000 cells from skeletal muscle were sequenced. The dataset was sequenced on a 10X Chromium scRNA-seq platform. We used the preprocessed UMI counts, obtained using Cellranger version 1.3 (10X Genomics). Downstream analysis using Seurat version 3.0.0 [62] identified 12 known cell types in adipose and 13 known cell types in muscle.

#### Simulated T-cell dataset

Human T cells of >50,000 resting and activated cells have been isolated from mucosal and lymphoid sites of 2 adult deceased organ donors and blood from 2 healthy donors [46]. The scRNA-seq data were downloaded from the Gene Expression omnibus (GEO) under accession number GSE126030. Similar to the metformin dataset, we applied filters to retain only genes expressed in at least across 10% of the cells to ensure we only included genuinely expressed genes.

#### PBMCs from patients hospitalized for COVID-19

This dataset consists of scRNA-sequencing data of PBMCs from 7 patients hospitalized for COVID-19 (male patients) and 6 healthy controls (4 male and 2 female) [43]. The scRNA-seq data of PBMCs from the COVID-19 patients were downloaded from the GEO under accession number GSE150728. We used information on the 20 cell types annotated along with the patient ID and gender as covariates in our modeling framework.

#### PBMCs from human volunteers

The scRNA-sequencing data of PBMCs were collected from 8 volunteers enrolled in an HIV vaccine trial from the 3 time points at days 0, 3, and 7 following vaccination [48]. The data were downloaded from the GEO under accession number GSE164378. We used information on the 8 cell types annotated along with the volunteer ID as covariates in our modeling framework.

#### Simulation study for testing the differential distribution pipeline

To get a realistic set of model parameters, we first performed model classification using our pipeline on the 3k-cell PBMC dataset to learn parameters of the 4 distributions for each gene. The 3k PBMCs were blood samples from 1 healthy donor generated using the v1. chemistry and preprocessed with CellRanger 1.1.0. [63]. We then generated UMI counts using parameters estimated for each gene by sampling a total cell UMI count from the input data. Using the model classification and parameter estimation, we simulated count data for 3 sample sizes: 2,638, 3k, and 5k cells. The scRNA-seq counts were generated using the R functions *rpois* and *rnegbin* which were from the R packages **stats** and **MASS**, respectively. The R functions *rzipois* and *rzinegbin* were available from the R package **VGAM**. We then applied our framework on the simulated data and checked for summary statistics such as accuracy, sensitivity, and specificity.

Accuracy of the simulation study reflects the ability of *scShapes* to differentiate between the 4 different distributions considered. This is calculated as

\begin{equation*} {\mathrm{Accuracy}} = \ \frac{{{\mathrm{TP}} + {\mathrm{TN}}}}{{{\mathrm{TP}} + {\mathrm{TN}} + {\mathrm{FP}} + {\mathrm{FN}}}}
\end{equation*}

Sensitivity of the simulation study reflects the ability of *scShapes* to determine the correct distribution or the true positives. This is calculated as

\begin{equation*} {\mathrm{Sensitivity}} = \ \frac{{{\mathrm{TP}}}}{{{\mathrm{TP}} + {\mathrm{FN}}}}
\end{equation*}

Specificity of the simulation study reflects the ability of *scShapes* to determine the true negatives. This is calculated as

\begin{equation*} {\mathrm{Specificity}} = \ \frac{{{\mathrm{TN}}}}{{{\mathrm{TN}} + {\mathrm{FP}}}}
\end{equation*}

where, for a given gene that is known to be following a Poisson distribution,

True positive (TP) = the number of cases correctly identified as Poisson;
False positive (FP) = the number of cases incorrectly identified as Poisson;
True negative (TN) = the number of cases correctly identified as NB, ZIP, or ZINB; and
False negative (FN) = the number of cases incorrectly identified as NB, ZIP, or ZINB.

#### Evaluating the significance of overlapping gene sets

To test the significance of overlapping sets of genes, when applying *scShapes* with and without accounting for known sources of biological variability, we used the R package *SuperExactTest* [64], both for calculating the significance of overlap between the gene sets and for visualizations.

#### Pathway overrepresentation analysis

Pathway overrepresentation analysis was done using the MSigDB—GSEA online tool by the Broad Institute [65]. This tool calculates the overlap between user-provided gene sets and that of the MSigDB collection of datasets chosen by the user. An estimate of the statistical significance of the overlapping gene set is provided based on the hypergeometric *P*value, which is corrected for multiple hypothesis testing using the BH method. All overlaps significant at *P* < 0.05 were selected with visualization of top significant pathways. Visualization of the significant pathways was done using the package *ggplot2* in R version 4.0.2.

#### Methods comparison between scShapes, edgeR, and scDD

*edgeR* (RRID:SCR_012802) [38] uses a negative binomial generalized linear model for differential expression testing. The quasi-likelihood pipeline using the function *glmQLFTest* implemented in *edgeR* was used for identifying the differentially expressed genes in the pairwise comparisons of interest, as this method allows for stricter error control than other pipelines of *edgeR*. Lowly expressed genes were filtered (expressed in less than 10% of the cells) and the mouse ID and cellular detection rate (average number of genes expressed per cell) used as covariates to account for the differences in the mice and sequencing depth, respectively. Genes were corrected using the BH correction, and only genes that had a corrected *P*value of <0.01 were considered significantly differentially expressed.

*DESeq2* (RRID:SCR_015687) [39] facilitates the study of RNA-seq data where the estimates of dispersion and fold change are computed using shrinkage estimation. *DESeq2* also implements model-based normalization where it normalizes for the differences in sequencing depth between samples using the median-of-ratios method. Read counts are then modeled as following an NB distribution using GLM with a logarithmic link. In order to minimize the effect of noisy estimates on differential expression testing resulting from highly variable dispersion parameter estimates, information is shared across all the genes when estimating the dispersion parameter.

*DEsingle* [40] utilizes a ZINB model to estimate the proportion of real and dropout zeros. Parameter estimation of the ZINB model is done using constrained MLE of the 2 ZINB populations. *DESingle* utilizes the likelihood ratio test for differential expression analysis and classifies the Diffentially Expressed Gene (DEG) into 3 types: (i) DEs, different expression status; (ii) DEa, differential expression abundance; and (iii) DEg, general differential expression. DEs refers to the genes showing a significant change in the percentage of zeros only between the 2 groups. DEa refers to the DEG between the groups but does not have a significant percentage of zeros between the 2 groups. Finally, DEg refers to the genes that are both differentially expressed (DE) between the groups and also have significant percentage of zeros between the groups.

*scDD* [12] utilizes a Bayesian modeling scheme to determine genes with differential distributions, classifying them into 1 of 4 categories: differential unimodal (DU), differential modality (DM), differential proportion (DP), and DB (both DM and DU). Similarly lowly expressed genes were filtered and scran Normalization implemented using the *preprocess* function from *scDD*. Based on the output from the function, *scDD* genes falling into 1 of the 4 categories were considered differentially distributed.

#### Assessing the prevalence of housekeeping genes

To overlap genes with housekeeping genes, we used a previously published list of ubiquitous genes from He et al. (2020) [19].

#### Sampling cells in a density-dependent manner

During the benchmarking of *scShapes*’ computational needs, one sampling strategy we followed was to sample cells in a density-dependent manner as previously followed by [49]. Briefly, 1,000 cells from each cell type were subsampled such that the probability of selecting a cell $c$ was $\frac{1}{{d( {{\mathrm{lo}}{{\mathrm{g}}_{10}}{N_c}} )}}$, where $d$ is the density estimate of ${\mathrm{lo}}{{\mathrm{g}}_{10}}( {{\mathrm{total\ cell\ UMIs}}} )$ and ${N_c}$ the total UMI counts in cell $c$.

## Availability of Source Code and Requirements

- Project name: *scShapes*
- Project homepage: https://github.com/Malindrie/scShapes and https://github.com/Malindrie/paper-scShapes
- Operating system(s): Platform independent
- Programming language: R
- Other requirements: R 4.1 or higher
- License: GPL-3
- Any restrictions to use by nonacademics: None
- RRID:  RRID:SCR_022838

## Data Availability

The metformin aging data have been deposited in GEO with accession number GSE194386.

Details on additional results are provided in the supplement. All supporting data and materials are available in the *GigaScience* GigaDB database [66].

## Abbreviations

BH: Benjamini and Hochberg; BIC: Bayesian information criterion; BL: blood; BM: bone marrow; DB: both differential modality and different component means; DD: differential distribution; DE: differential expression; DM: differential modality; DP: differential proportion; DZ: differential zeros; GLM: generalized linear model; GO: Gene Ontology; KEGG: Kyoto Encyclopedia of Genes and Genomes; KS: Kolmogorov–Smirnov; LG: lungs; LN: lymph node; LRT: likelihood ratio test; NB: negative binomial; NK: natural killer; PBMC: peripheral blood mononuclear cell; QLF: quasi-likelihood approach; scRNA-seq: single-cell RNA sequencing; ZINB: zero-inflated negative binomial; ZIP: zero-inflated Poisson.

## Supplementary Materials

**Supplementary Fig. S1**. Investigating how the zero-inflation parameter is associated with the prevalence of zeros. Scatterplot depicts the distribution of the zero-inflation parameter against the percentage of zeros in zero-inflated features across all cells in (A) adipose and (B) muscle. The zero-inflation parameter π represents the probability of observing excess zeros in genes that were detected to be following either a ZIP or ZINB distribution and is compared against the percentage of zeros in the corresponding gene.

**Supplementary Fig. S2**. Pathway overrepresentation analysis of differentially distributed genes. The top 10 significant pathways at a BH-adjusted *P* < $1\ \times {10^{ - 4}}$ in the pairwise comparisons of (A) Old to Young in adipose, (B) Old to Treated in adipose, (C) Old to Young in muscle, and (D) Old to Treated in muscle using KEGG pathways. The length of the bar corresponds to the percentage of differentially distributed genes that overlapped with the curated pathway gene list.

**Supplementary Fig. S3**. Pathway overrepresentation analysis of genes switching between unimodal distributions to zero-inflated distributions. Genes reverting expression distribution from a Poisson or NB in young/metformin treated to ZIP or ZINB in old in the pairwise comparisons of (A) Young to Old in adipose, (B) Treated to Old in adipose, (C) Young to Old in muscle, and (D) Treated to Old in muscle using Hallmark pathways. Significant pathways (BH-adjusted *P* < $5\ \times {10^{ - 2}}$) are represented where the length of the bar corresponds to the percentage of switching genes that overlapped with the Hallmark gene set.

**Supplementary Fig. S4**. Pathway overrepresentation analysis of genes identified to be differentially distributed by both *scShapes* and *scDD*. The Venn diagrams for the number of genes detected to be DD by *scShapes* and *scDD*. For the overlapping gene sets, we performed pathway overrepresentation analysis using Hallmark pathways. Significant pathways (BH-adjusted *P* < $5\ \times {10^{ - 2}}$) are represented where the length of the bar corresponds to the percentage of switching genes that overlapped with the Hallmark gene set.

**Supplementary Fig. S5**. (A) Overview of the study design for scRNA-seq of COVID-19 patients and controls. Single cells of PBMCs have been profiled from 7 male patients hospitalized for COVID-19 and 4 male and 2 female healthy controls. Quality control, normalization, dimensionality reduction, clustering, and differential gene expression analysis have been done using the R package Seurat. Comparing the marker genes per cluster with that of cell-type specific gene from the literature has resulted in 20 cell types, which have been confirmed using the R package *SingleR*. Pathway overrepresentation analysis of differentially distributed genes between COVID-19 data and healthy controls using (A) GO pathways, (B) KEGG pathways, and (C) Hallmark pathways. The length of the bar corresponds to the percentage of differentially distributed genes that overlapped with the curated pathway gene list, and the color of the bar corresponds to the BH-adjusted *P* value for the overlap.

**Supplementary Fig. S6**. (a) Bar plot for overlap of differentially distributed genes at cell-type level between COVID-19 patients and healthy controls. We checked for the COVID-19–driven changes in gene expression distribution at cell-type level and focused our attention on 7 main cell types: NK cells, B cells, dendritic cells (DCs), T cells (CD4 and CD8), and monocytes (CD14 and CD16). The bar plot summarizes the overlap between the genes that switch gene expression distribution between COVID-19 and healthy control groups for different combinations of cell types under study. The height of each bar corresponds to the number of overlapping genes switching distribution, and the color corresponds to the *P*-adjusted value for the overlap. (B) Pathway overrepresentation analysis of commonly differentially distributed genes between NK and T cells. The length of the bar corresponds to the BH-adjusted *P* value of the overlap of DD genes in NK and T cells. The length of the bar corresponds to the percentage of differentially distributed genes that overlapped with the curated pathway gene list, and the color of the bar corresponds to the BH-adjusted *P* value for the overlap.

**Supplementary Fig. S7**. (A) Overview of the study design for scRNA-seq of stimulated T cells. Single cells with and without stimulation have been isolated from lungs, lymph nodes, bone marrow of deceased donors, and blood (PBMCs) from healthy donors for comparison. Single-cell sequencing libraries have been sequenced using 10X Chromium Single Cell 3′ Solution. Pathway overrepresentation analysis of differentially distributed genes in (B) BM, (C) BL, (D) LG, and (E) LN between resting and activated T cells using GO pathways. Here, the top 20 significant pathways are visualized. The length of the bar corresponds to the percentage of differentially distributed genes that overlapped with the curated pathway gene list, and the color of the bar corresponds to the BH-adjusted *P* value for the overlap.

**Supplementary Fig. S8**. Quality metrics for metformin mouse dataset—adipose. (A) Number of cell counts per sample. We see over 4,000 cells per sample, which is a bit more than the 3,000 cells expected. (B) Number of UMIs/transcripts per cell. We can see that cells in all samples have 1000 UMIs or greater. (C) Number of genes detected per cell. (D) Mitochondrial counts ratio. (E) UMIs vs. genes detected. Correlation between genes detected and number of UMIs to determine whether strong presence of cells with low numbers of genes/UMIs.

**Supplementary Fig. S9**.: Quality metrics for metformin mouse dataset—muscle. (A) Number of cell counts per sample. We see that except for in the Treated sample, the other 2 samples have cells fewer than 3,000, which is a bit less than the 3,000 cells expected. (B) Number of UMIs/transcripts per cell. We can see that majority of cells in all samples have 1,000 UMIs or greater. (C) Number of genes detected per cell. (D) Mitochondrial counts ratio. (E) UMIs vs. genes detected. Correlation between genes detected and number of UMIs to determine whether strong presence of cells with low numbers of genes/UMIs.

**Supplementary Table S1**. Classification of genes in the metformin data. Genes were classified as 1 of 4 count models (Poisson, NB, ZIP, or ZINB). Table shows the number of genes in each category: (A) using a GLM with the offset term to account for cell sequencing depth and mouse ID as an explanatory covariate and (B) using a GLM that also includes cell type as an explanatory covariate.

**Supplementary Table S2**. Pattern of differential distribution in genes detected as differentially distributed by *scShapes*.

**Supplementary Table S3**. Investigating whether switching genes were also detected as differentially expressed by (A) *edgeR*, (B) *DESeq2*, and (C) *DEsingle*.

**Supplementary Table S4**. Breakdown of distribution shapes of genes that were detected to be both differentially distributed by *scShapes* and differentially expressed by (A) *edgeR*, (b) *DESeq2*, and (C) *DEsingle*.

**Supplementary Table S5**. Distribution pattern of DZ genes detected by *scDD* that overlap with the DD genes from *scShapes*.

**Supplementary Table S6**. Classification of genes in the COVID-19 dataset.

**Supplementary Table S7**. Classification of genes in the human T-cell data.

**Supplementary Table S8**. Differentially distributed genes between resting and stimulated T cells.

## Funding

This work is supported by an Australian Research Council Future Fellowship(FT170100047), a Georgina Sweet Award to J.C.M., and an Australasian Genomic Technologies Association (AGTA) PhD Top-Up Scholarship to M.D.

## Authors’ Contributions

J.C.M., A.T.F., and M.D. formulated the problem. M.D. developed the *scShapes* method and software with input from J.C.M. and A.T.F. M.D, designed and implemented the simulations and applied *scShapes* to the case studies on real scRNA-seq data. J.C.M., A.T.F., and M.D. interpreted the results with input from A.S.K. J.C.M., and A.T.F. M.D. wrote the manuscript with input from A.S.K. All authors read and approved the final version of the manuscript.

## Competing Interests

The authors declare that they have no competing interests.

## Supplementary Material

giac126_GIGA-D-22-00034_Original_Submission

giac126_GIGA-D-22-00034_Revision_1

giac126_GIGA-D-22-00034_Revision_2

giac126_Response_to_Reviewer_Comments_Original_Submission

giac126_Response_to_Reviewer_Comments_Revision_1

giac126_Reviewer_1_Report_Original_Submission Shiping Liu, Ph.D -- 4/10/2022 Reviewed

giac126_Reviewer_1_Report_Revision_1 Shiping Liu, Ph.D -- 9/26/2022 Reviewed

giac126_Reviewer_2_Report_Original_Submission Yuchen Yang -- 5/2/2022 Reviewed

giac126_Reviewer_2_Report_Revision_1 Yuchen Yang -- 9/12/2022 Reviewed

giac126_Supplemental_Files
