# Supplementary material for: scShapes: a statistical framework for identifying distribution shapes in single-cell RNA-sequencing data
Source: Gigascience. 2023 Jan 24;12:giac126. doi: 10.1093/gigascience/giac126 (PMC9871437; doi:10.1093/gigascience/giac126)
Supplement: giac126_GIGA-D-22-00034_Revision_1 [file giac126_giga-d-22-00034_revision_1.pdf]

GigaScience

scShapes: A statistical framework for identifying distribution shapes in single-cell RNA-sequencing data.

--Manuscript Draft--

Manuscript Number:	GIGA-D-22-00034R1
Full Title:	scShapes: A statistical framework for identifying distribution shapes in single-cell RNA-sequencing data.
Article Type:	Research
Funding Information:	Australian Research Council (FT170100047) — Dr. Jessica C. Mar
Abstract:	Background<br><br>Single cell RNA sequencing (scRNA-seq) methods have been advantageous for quantifying cell-to-cell variation by profiling the transcriptomes of individual cells. For scRNA-seq data, variability in gene expression reflects the degree of variation in gene expression from one cell to another. Analyses that focus on cell-cell variability therefore are useful for going beyond changes based on average expression and instead, identifying genes with homogenous expression versus those that vary widely from cell to cell.<br><br>Results<br><br>We present a novel statistical framework scShapes for identifying differential distributions in single-cell RNA-sequencing data using generalized linear models. Most approaches for differential gene expression detect shifts in the mean value. However, as single cell data are driven by over-dispersion and dropouts, moving beyond means and using distributions that can handle excess zeros is critical. scShapes quantifies gene-specific cell-to-cell variability by testing for differences in the expression distribution while flexibly adjusting for covariates if required. We demonstrate that scShapes identifies subtle variations that are independent of altered mean expression and detects biologically-relevant genes that were not discovered through standard approaches.<br><br>Conclusions<br><br>This analysis also draws attention to genes that switch distribution shapes from a unimodal distribution to a zero-inflated distribution and raises open questions about the plausible biological mechanisms that may give rise to this, such as transcriptional bursting. Overall, the results from scShapes helps to expand our understanding of the role that gene expression plays in the transcriptional regulation of a specific perturbation or cellular phenotype. Our framework scShapes is incorporated into Bioconductor R package (https://github.com/Malindrie/scShapes).
Corresponding Author:	Jessica Mar<br><br>AUSTRALIA
Corresponding Author Secondary Information:	
Corresponding Author's Institution:	
Corresponding Author's Secondary Institution:	
First Author:	Malindrie Dharmaratne
First Author Secondary Information:	
Order of Authors:	Malindrie Dharmaratne<br>Ameya S Kulkarni

Powered by Editorial Manager® and ProduXion Manager® from Aries Systems Corporation

	Atefeh Taherian Fard
	Jessica C. Mar
**Order of Authors Secondary Information:**	
**Response to Reviewers:**	Please see uploaded file of Responses to Reviewers Comments
**Additional Information:**	
**Question**	**Response**
Are you submitting this manuscript to a special series or article collection?	No
**Experimental design and statistics**<br><br>Full details of the experimental design and statistical methods used should be given in the Methods section, as detailed in our Minimum Standards Reporting Checklist. Information essential to interpreting the data presented should be made available in the figure legends.<br><br>Have you included all the information requested in your manuscript?	Yes
**Resources**<br><br>A description of all resources used, including antibodies, cell lines, animals and software tools, with enough information to allow them to be uniquely identified, should be included in the Methods section. Authors are strongly encouraged to cite Research Resource Identifiers (RRIDs) for antibodies, model organisms and tools, where possible.<br><br>Have you included the information requested as detailed in our Minimum Standards Reporting Checklist?	Yes
**Availability of data and materials**<br><br>All datasets and code on which the conclusions of the paper rely must be either included in your submission or deposited in publicly available repositories (where available and ethically	Yes

appropriate), referencing such data using a unique identifier in the references and in the “Availability of Data and Materials” section of your manuscript.<br><br>Have you have met the above requirement as detailed in our Minimum Standards Reporting Checklist?	

Revised Manuscript

Click here to access/download;Manuscript;scShapes_MD_GS_120822

scShapes: A statistical framework for identifying distribution shapes in single-cell RNA-sequencing data.

Formatted: Numbering: Continuous

Malindrie Dharmaratne[1], Ameya S Kulkarni[2, 3], Atefeh Taherian Fard[1*], Jessica C Mar[1*]

[1]Australian Institute for Bioengineering and Nanotechnology, The University of Queensland, Brisbane, QLD, 4072, Australia

[2]Institute for Aging Research, Albert Einstein College of Medicine, Bronx, New York, USA

[3]Department of Medicine, Division of Endocrinology, Albert Einstein College of Medicine, Bronx, New York, USA

[*]Correspondence to JCM (jessica.mar@uq.edu.au); ATF (a.taherianfard@uq.edu.au).

Abstract

Background

Single cell RNA sequencing (scRNA-seq) methods have been advantageous for quantifying cell-to-cell variation by profiling the transcriptomes of individual cells. For scRNA-seq data, variability in gene expression reflects the degree of variation in gene expression from one cell to another. Analyses that focus on cell-cell variability therefore are useful for going beyond changes based on average expression and instead, identifying genes with homogenous expression versus those that vary widely from cell to cell.

Results

We present a novel statistical framework *scShapes* for identifying differential distributions in single-cell RNA-sequencing data using generalized linear models. Most approaches for differential gene expression detect shifts in the mean value. However, as single cell data are

driven by over-dispersion and dropouts, moving beyond means and using distributions that can handle excess zeros is critical. *scShapes* quantifies gene-specific cell-to-cell variability by testing for differences in the expression distribution while flexibly adjusting for covariates if required. We demonstrate that *scShapes* identifies subtle variations that are independent of altered mean expression and detects biologically-relevant genes that were not discovered through standard approaches.

Conclusions

This analysis also draws attention to genes that switch distribution shapes from a unimodal distribution to a zero-inflated distribution and raises open questions about the plausible biological mechanisms that may give rise to this, such as transcriptional bursting. Overall, the results from *scShapes* helps to expand our understanding of the role that gene expression plays in the transcriptional regulation of a specific perturbation or cellular phenotype. Our framework *scShapes* is incorporated into Bioconductor R package (https://github.com/Malindrie/scShapes).

Keywords

Single-cell RNA-sequencing, Distribution shapes, Zero-inflation.

Background

Variation in gene expression from one cell to another plays an instrumental role in how tissues develop and function [1], and therefore consideration of the distribution shape of a gene's expression profile is important for understanding transcriptional regulation [2]. The analysis of scRNA-seq data sets has provided a means to quantitatively study cell-to-cell variation in response to phenotypic changes and as a result, new rare and complex cell populations have

been identified [3], regulatory relationships among genes have been discovered [4], and trajectories of cell lineages in development and disease have been elucidated [5]. However, a major limitation of existing scRNA-seq methods is that these analyses tend to focus on detecting changes in average expression rather than changes in variability or other properties of the distribution. While transcriptional regulation requires both, a significant strength of scRNA-seq data is the opportunity to model gene expression variability and its role in regulating cellular phenotypes.

Existing scRNA-seq methods assume that a single parametric distribution is adequate for modelling the shape of a gene's expression profile. While this assumption may have practical advantages, it does limit the ability to discover new relationships or gene expression shape patterns. A property of scRNA-seq data is its increased sparsity compared to bulk gene expression data, where an abundance of cells may have unobserved expression levels due to either biological or technical sources of cell-to-cell variability [6, 7]. As a result, it may be an over-simplification to assume that the expression profiles of genes across the transcriptome may be adequately explained by a single parametric distribution. A limitation of this assumption may be that some genes are overlooked or modelled sub-optimally as we fail to first evaluate the prevalence of different distributions in a scRNA-seq dataset [2].

To model the heterogeneity of gene expression data under a statistical framework, it is vital that the distribution with the most appropriate fit for each gene's expression profile be used [8]. While some statistical methods have appealed to the use of mixture models [6, 9] as an alternative to the widely-used negative binomial distribution [10, 11], they fail to investigate the range of different gene expression distributions that may be present in the scRNA-seq data as a first step. In *scShapes*, we address this issue directly by proposing a gene-specific framework for identifying differential distributions from a selection of relevant candidate distributions.

Currently differentially distributed genes can be assessed using the *R* package *scDD* [12]. However, this approach is unable to adjust for covariates directly in the model. Because accounting for covariates, especially technical ones like gender or hospital site, may be necessary to address for the underlying question being asked of the scRNA-seq analysis, this limitation of *scDD* has practical implications as the size and complexity of scRNA-seq datasets grow. The *scDD* method also only permits pairwise comparisons between two biological conditions. In our *scShapes* framework, we propose an approach for modelling read counts from droplet-based scRNA-seq experiments using generalized linear models (GLM) with error distributions belonging to the family of zero-inflated negative binomial distributions.

Zero counts are a prevalent feature of scRNA-seq data that present challenges for statistical modelling, mainly because these zero values result from different zero generating processes within the same biological system [13]. Because these zeroes can arise from biological and technical sources, the treatment of the zero values, e.g. through imputation or filtering, is not straightforward. The zero values observed in scRNA-seq can be the result of limitations in sequencing depth and capture efficiency, such that only a small percentage of the transcripts present end up being counted and low abundance transcripts can go undetected [14, 15]. Observing a zero count can also be due to biological factors like a gene that is simply not expressed in a specific cell type or state or the fact that transcription is a stochastic process which gives rise to these additional zeros [16]. By taking all these factors into account, we assume that the overabundance of zeros in scRNA-seq data is gene-specific and that each gene therefore requires a statistical model with its own set of distributional assumptions. To implement this, our framework models gene expression data for a mix of unimodal and zero-inflated distributions to identify the distribution that provides the most appropriate fit for each gene (Figure 1).

Here we develop a framework, *scShapes*, which relies upon modelling read counts using GLMs and matches a distribution shape to a gene's expression distribution for an individual gene. The gene expression distribution is modelled using both unimodal and zero-inflated distributions, where for accurate evaluation of zero-inflation we conduct a modified likelihood ratio test (LRT) [17]. Our framework has been designed to identify subtle variations from one cell to another that is not weighted towards a change in mean. It also has the flexibility to adjust for covariates and perform multiple comparisons between sample or treatment groups while explicitly modelling the variability of gene expression occurring between cells. Using simulation studies, we show that *scShapes* can reliably detect zero-inflated genes, and when applied to a range of scRNA-seq published datasets, *scShapes* can identify genes and pathways linked to the phenotype of interest that were not discovered through standard analyses of transcriptomic data. The flexibility of *scShapes* is also demonstrated through use cases that have been applied to scRNA-seq datasets that feature distinct experimental designs and experimental models.

Data Description

A collection of 3 publicly available scRNA-seq datasets were used in this study. These datasets were downloaded from NCBI GEO (accession numbers and data descriptions given under 'Datasets used'). For all the datasets cell-type identification has been performed by their respective original publications, which we have used in the *scShapes* framework as prior biological knowledge. For each dataset, genes with non-zero expression in at least 10% of all cells within a treatment condition are retained and were used for all subsequent analyses.

Analyses

An overview of the *scShapes* framework.

The entry point to the *scShapes* pipeline is a set of aligned read counts from a scRNA-seq experiment. For each treatment condition, we model a gene independently using the error distributions from all four possible distributions, Poisson, Negative Binomial (NB), Zero-inflated Poisson (ZIP) and Zero-inflated Negative Binomial (ZINB) with a log link function. A model-based normalization is applied to the read counts to account for differences in sequencing depth between libraries. This is done by including the log10 of the total UMI counts assigned per cell as an offset in the GLM model so that differences in sequencing depth are directly adjusted for. Furthermore, additional covariates can be incorporated in the GLM framework to account for any biases introduced by biological replicates or technical attributes of the experimental design like batch effects. The most appropriate model is first selected based on the Bayesian Information Criterion (BIC) and LRT statistic, to ensure the best distribution is selected for each gene (see Figure 1 and Methods for more details).

The overabundance of zeros in scRNA-seq data is gene-specific.

To demonstrate the *scShapes* differential distribution framework, we apply this to a scRNA-seq dataset collected for an ageing study on two tissues, adipose and muscle, from mice (Figure 2a). In the study design, there are three groups of mice which we designate as Young, Old and Treated, where each group consists of four male mice as biological replicates (see Methods). GLM models with error distributions from the Poisson, NB, ZIP and ZINB distributions are fitted without assuming any prior biological knowledge and correcting only for the technical variability between cells. This was done by including an offset in the GLM model to account for differences in sequencing depth and the mouse ID included as an explanatory covariate in

the GLM to account for any biases introduced due to one mouse being potentially different from any of its other biological replicates.

We found that the majority of the genes (between 52%-62% in adipose and between 40%-45% in muscle) follow a NB distribution in both tissue types (Figure 2c). In the muscle tissue, it is worth highlighting that at least 50% of the genes do not follow the NB distribution under the three treatment conditions. Collectively, over 55% of genes follow either a NB, ZIP or ZINB distribution and this observation aligns with the nature of scRNA-seq data which is driven by over-dispersion and zero-inflation. These results provide evidence that in fact not all genes have expression profiles that follow a NB distribution and more importantly, demonstrate that not all the genes in the transcriptome follow a single distribution either.

The inclusion of prior biological knowledge, like cell type membership, changes the shape of a gene's expression distribution.

We investigated whether accounting for known biological knowledge such as the membership of a specific cell type affects the classification of genes into their distribution shape. The ageing mouse dataset consists of 13 known cell types in adipose and 12 known cell types in muscle (Figure 2b). To account for the biological variation in the data, we introduced the cell type information as an explanatory covariate in the GLM model. Accounting for cell type membership resulted in a marked reduction in the number of NB-distributed genes, a resulting increase in Poisson-distributed genes, and the number of zero-inflated genes also decreased (Figure 2d & Table S1). This observation is in line with Choi *et al*. [18], which also observed that adjusting for cell type resulted in a reduction of the number of zero-inflated genes.

Housekeeping genes are required for the maintenance of basic cellular functions, and hence are usually uniformly expressed in all cells with low variance. Interestingly we found that around 50% of the genes under each condition that remained Poisson-distributed even after accounting

for cell type membership were previously described as having a role as a housekeeping gene [19] (Figure 3). The overlap between the Poisson-distributed genes with and without accounting for known cell types was significantly different only in adipose (adjusted P-value < 0.01, Figure 3a). A gene which remains Poisson-distributed regardless of whether cell-type specific expression rates were taken into account are indicative of housekeeping genes as the rate of expression in these genes do not vary across cell types. Our finding indicates that a Poisson error model is sufficient to explain the variability in the expression of genes with a housekeeping function.

A simulation study to evaluate the sensitivity of the *scShapes* gene expression distribution classification.

We designed a simulation study to evaluate our framework's ability to accurately classify genes into Poisson, NB, ZIP and ZINB distributions. To derive a realistic set of model parameters, we first performed model classification using the *scShapes* framework for the well-known 3k-cell PBMC dataset. The peripheral blood mononuclear cells (PBMCs) were downloaded from the 10X Genomics website (https://www.10xgenomics.com/resources/datasets). Using the model classification and parameter estimation we simulated count data for three sample sizes, 2638, 3000 and 5000 cells (see Methods section). As expected, we see that as the sample size increases, the ability of our framework to correctly identify the best fit distribution increases (Figure 4). However, the more striking result is that our framework can accurately identify the correct model distribution for each gene with an accuracy of 85% or above for all four distributions across the range of sample sizes tested (Figure 4a).

The zero-inflation parameter can be interpreted as an estimate of biological zeros.

Being able to distinguish the zeros that reflect technical drop-outs versus genuine biological zeros is helpful for understanding how genes control cellular phenotypes. The zero-inflation

parameter $\hat{\pi}_0$ in the *scShapes* model gives an estimate of the proportion of structural zeros for each gene which may be indicative of the subset of cells where the transcript is truly absent. These models make the assumption that the zero observations have two different origins namely, **structural zeros assumed to be observed due to specific structure in the data** which are distinct from **random zeros which are assumed to be observed due to sampling variability**. In order to evaluate the relationship between the estimated zero-inflation parameter $\hat{\pi}_0$ and the percentage of zeros across all cells in genes detected to be zero-inflated (*i.e*. genes following either a ZIP or ZINB distribution), we plotted the estimated zero-inflation parameter against the percentage of zeros across cells for each zero-inflated gene (Figure S1). While most features have at least 50% or more zeros across cells, the zero-inflation parameter can vary between any value between 0 and 1. This indicates that there is either no relationship or a very weak relationship between the zero-inflation parameter and the percentage of zeroes. It demonstrates that genes with a high percentage of zeroes overall are not necessarily captured by models where the zero-inflation parameter is high. Choi *et al*. [18] has shown that the primary cause of zero inflation is due to biological variation and not only due to technical variation. So, one can assume that the excessive zeros we observe in single-cell data are the result of the two zero generating processes; where either a transcript is absent from a biological system due to sampling variability or where a transcript is truly absent from the biological system. Hence, modelling genes with excess zeros using zero-inflated distributions will be ideal in such a scenario.

We also leveraged the zero-inflation parameter to identify genes that had an excessively high number of structural zeros. For example around 98% of the zeros in the gene *Arhgap20* were structural zeros in adipose under the Old condition. This gene encodes a protein which is an activator of RHO-type GTPases. It has been shown to be associated with Alzheimer's disease and predicted to be a dysregulated gene [20]. Similarly, the gene *Sfxn1* which has around 85%

of structural zeros based on the estimate of the zero-inflation parameter in muscle under the Old condition, is found to be downregulated in senescence (SeneQuest), a process that is a causal factor for aging tissues [21]. *Sfxn1* is a protein-coding gene which mediates the transportation of serine to the mitochondria. Although the mitochondrial biology of sideroflexin (SFXN) family are not entirely explored, dysfunction in mitochondrial carriers results in perturbations in oxidative phosphorylation which underlies various pathologies including aging related diseases [22]. Hence it could be hypothesized that the higher number of structural zeros in *Sfxn1* could be as a result of aging associated mitochondrial dysfunction with aging in the muscle. These results highlight the possibility that the estimates of the zero-inflation parameter might be indicative of features which are truly absent from a biological system and therefore play a role in the regulation of the cellular phenotype under study.

Around 30% of the genes undergo change in shape of distribution with aging or treatment, and collectively these differentially-distributed genes demonstrate a significant enrichment of aging-related pathways.

Since we observed a considerable change in the composition of unimodal genes to zero-inflated genes after accounting for known cell types, this suggests that accounting for cell type-specific effects is important for addressing biological information about distributional shapes. To investigate the biological inference from enriched pathways of differentially distributed genes, we performed pathway over-representation analysis on the model distribution classifications obtained after taking into account cell type-specific gene expression for the mouse dataset by adjusting for cell type as a covariate. We identified the genes that switched their distributions either between Old vs Young or Old vs Treated. Nearly, 30% of the genes (after filtering, i.e. of the genes with at least 10% expression across all cells within a treatment condition) were differentially distributed in at least one of the comparisons (Old vs Young or Old vs Treated) in both tissues adipose and muscle. The top 10 Hallmark pathways and KEGG pathways most

over-represented among the genes switching distributions are represented in Figures 5 & S2 respectively.

The detection of statistically significant pathways provides evidence that the differentially distributed genes detected by our *scShapes* framework are enriched for biological processes related to aging. Consideration of what these processes are help to illustrate how these genes may be participating in processes that are altered through aging. For example, in the Hallmark pathways, one of the most commonly enriched most significant pathways include *DNA damage*, where excessive DNA damage or poor DNA repair contributes to the aging process [23]. Among the most enriched KEGG pathways in the Treated group in adipose is the *insulin signalling pathway* which is one of the primary nutrient-sensing pathways targeted by metformin [24]. Furthermore, the *MAPK signalling pathway*, is involved in the regulation of differentiation, cell-growth, proliferation and apoptosis [25] and is enriched in both adipose and muscle in the treated cells. Metformin is found to be relevant in activating the MAPK signalling pathway and increases the expression of DNA damage and growth inhibition gene *GADD153* [26]. Among the differentially distributed genes we find the transcription factors (TFs) *FOXO3* involved in regulating aging-associated stress response and proposed to be mediated by metformin [27, 28], *RXRA*, overexpression of which reduces DNA damage accumulation leading to delays in replicative senescence [29] and previously shown to be targeted by metformin in human adipose [30], to be common in both Old vs Young and Old vs Treated comparisons in adipose. We then further investigated some of the differentially distributed genes identified by *scShapes*. For example, we observed that the gene Foxo3, identified to be differentially distributed in both pairwise comparisons following a NB distribution and then switch distribution to a Poisson in both young/treated. This result indicates that during ageing we observe higher over-dispersion in the expression of Foxo3, than that could be modelled with a Poisson distribution. Hence it could be hypothesized that there

Formatted: Font: Italic

are increased levels gene expression heterogeneity observed during aging compared to young phenotype, where treatment with metformin reverts the expression of Foxo3 with lesser over-dispersion, to levels comparable with the young phenotype. This observation could have been missed if we only focused on DE genes, as Foxo3 was not observed to be differentially expressed in either comparison. -Similarly some of the TFs commonly differentially distributed in muscle in both Old vs Young and Old vs Treated include *SRF* reduction of which leads to premature aging in skeletal muscle [31] and *IRF3* a novel inhibitor of cellular senescence and inducer of cell growth inhibition [32].

Genes switching to zero-inflated distributions in the old condition may point to those genes involved in the regulation of transcriptional bursting.

According to Clivio *et al*. [33] zero-inflated genes may reflect other biological phenomenon such as transcriptional bursting. We investigated this hypothesis in the context of aging using the metformin-treated data set. With age, a loss of transcriptional regulation is accompanied by an increase in transcriptional noise [34]. As transcriptional bursting leads to increased levels of gene expression noise, we used *scShapes* to identify the set of genes that switched from a unimodal distribution in the Young group to a more heterogeneous pattern captured by the zero-inflated distribution in the Old group while also switching from a unimodal distribution to a zero-inflated one in the Treated to Old groups respectively (see Supplementary Figure S3). The rationale behind using the *scShapes* framework to detect this specific profile is that these genes become more heterogeneous in the presence of ageing (Young to Old) as well as in response to metformin-treatment (Treated to Old). Interestingly the top Hallmark enriched term for genes switching to zero-inflated distributions in old from both young and treated in muscle is *WNT beta catenin signalling* (Figure S3), a signaling pathway that may play a role in modulating gene expression noise [35]. Some of these genes include *HDAC5*, Histone Deacetylase which plays an important role in transcriptional regulation and is involved in the

chromatin dynamics of generating gene-specific noise [36]. These results draw our attention to the possibility that zero-inflated genes might be indicative of basic biological phenomena like transcriptional bursting. The use of the *scShapes* framework represents a methodological approach to detect this kind of unimodal to zero-inflated differential switching that may be useful for identifying further genes involved in the transcriptional bursting process. It is worth emphasizing that the flexibility of the *scShapes* framework and its range of distribution assumptions are what led to the identification of these genes, which would not have been necessarily detected by methods where a NB distribution is applied or assume the prevalence of a single distribution shape for all genes.

Comparison of *scShapes* with existing tools for differential gene expression analysis.

To compare the performance of our *scShapes* framework for discovering age-related gene expression to that of standard differential expression (DE), we carried out standard DE using *edgeR* [37], DESeq2 [38]; DEsingle [39] which utilizes a ZINB model for DE testing and also compared our framework against *scDD* [12] which models the change in mean expression levels by comparing gene expression distributions. *edgeR* was used to test for differential expression and the pairwise comparisons tested were between the Young vs Old and Old vs Treated groups. We applied *edgeR*'s quasi-likelihood approach (QLF) with cellular detection rate as a covariate, since this method was shown to perform well for DE analysis of scRNAseq data after filtering out lowly-expressed genes [40]. Similarly, *DESeq2* was used with published recommendations for single cell datasets, where a likelihood ratio test (LRT) was performed between the pairwise comparisons of interest to identify the differentially expressed genes. Genes were corrected using the Benjamini & Hochberg (BH) correction and only genes that had a corrected *P-value* of < 0.01 were retained. Although there were similar number of DE genes between *scShapes* versus *edgeR*, *DESeq2*, *DEsingle* and *scDD* (Table 1a), there is little overlap even ~~between *edgeR* and *scDD*~~ across existing methods (Table 1b). The genes found

Formatted: Font: Italic

by our *scShapes* method that are exclusively switching between a unimodal distribution and a zero-inflated distribution represent over 25% of the genes in adipose and over 45% of the genes in muscle. Some of the unique genes detected by our pipeline include the TFs *FOXO3*, *PKNOX1* involved in apoptosis, regulation of oxidative stress and DNA damage [41] in adipose. Similarly, the TFs *IRF3*, ~~*SRF*,~~ *TFE3* involved in autophagy are some of the uniquely detected genes by our pipeline in muscle.

Table 1: **Comparison of genes identified by *scShapes* with ~~two~~ four other methods, *edgeR*, *DESeq2*, *DEsingle* and *scDD*.** The total number of genes for *DEsingle* includes genes detected using the categories DEs, DEa and DEg. Similarly, ~~T~~the total number of genes for *scDD* includes genes detected using the categories DE, DP, DB, and DZ.

~~Tissue~~	~~Comparison~~	~~Number of Genes Detected by *scShapes*~~	~~Number of Genes Detected by *edgeR*~~	~~Number of Genes Detected by *scDD*~~	~~Overlap Between All 3 Gene Lists~~	~~Overlap between *edgeR* and *scDD*~~	~~Number of Genes Unique *scShape*~~[illegible]
~~Adipose~~	~~Old vs Young~~	~~902~~	~~2045~~	~~2291~~	~~102~~	~~796~~	~~402~~
	~~Old vs Treated~~	~~825~~	~~916~~	~~1214~~	~~17~~	~~183~~	~~605~~
~~Muscle~~	~~Old vs Young~~	~~988~~	~~519~~	~~338~~	~~9~~	~~35~~	~~840~~
	~~Old vs Treated~~	~~986~~	~~393~~	~~319~~	~~0~~	~~24~~	~~877~~

(a) No of genes detected to be either DE or DD by each method

Tissue	Comparison	*scShapes*	*scDD*	*edgeR*	*DESeq2*	*DEsingle*
Adipose	Old vs Young	902	2291	2045	1849	2104
	Old vs Treated	825	1214	916	438	891
Muscle	Old vs Young	988	338	519	424	457
	Old vs Treated	986	319	393	379	450

(b) Comparison of detected genes between methods

Tissue	Comparison	Overlap between all methods	Overlap between published methods	Overlap between DE methods

Formatted: Font: Italic
Formatted: Font: Italic
Formatted: Font: Italic
Formatted: Font: Italic
Formatted: Font: Italic
Formatted: Font: Not Bold
Formatted: Font: Not Bold, Italic
Formatted: Font: Not Bold
Formatted Table
Formatted: Space After: 0 pt
Formatted: Font: (Default) Times New Roman, 12 pt
Formatted: Line spacing: 1.5 lines
Formatted Table
Formatted: Font: (Default) Times New Roman, 12 pt
Formatted: Font: (Default) Times New Roman, 12 pt, Italic
Formatted: Font: (Default) Times New Roman, 12 pt
Formatted: Line spacing: 1.5 lines
Formatted: Font: (Default) Times New Roman, 12 pt
Formatted: Line spacing: 1.5 lines
Formatted: Font: (Default) Times New Roman, 12 pt
Formatted: Font: (Default) Times New Roman, 12 pt
Formatted: Line spacing: 1.5 lines
Formatted: Font: (Default) Times New Roman, 12 pt
Formatted: Line spacing: 1.5 lines
Formatted: Space After: 0 pt, Tab stops: 1.15", Left
Formatted: Line spacing: 1.5 lines
Formatted: Font: (Default) Times New Roman, 12 pt
Formatted: Centered, Line spacing: 1.5 lines
Formatted: Font: (Default) Times New Roman, 12 pt
Formatted: Font: (Default) Times New Roman, 12 pt

Adipose	Old vs Young	28	359	435
	Old vs Treated	1	25	30
Muscle	Old vs Young	4	21	29
	Old vs Treated	0	13	20

** published methods include edgeR, DESeq2, DEsingle and scDD*
** DE methods include edgeR, DESeq2 and DEsingle*

Of the genes that were detected as differentially distributed by our *scShapes* method, between 25% to 27% of the genes switch distributions from a unimodal to a zero-inflated distribution in adipose (Table S2a). However, in muscle around 50% of the differentially distributed genes switch distribution from a unimodal to a zero-inflated distribution. The genes switching distribution from a unimodal to a zero-inflated distribution are indicative of the genes that have excessive zeros in one condition compared to another condition. We further investigated the patterns of differential distributions to identify what percentages of genes switched distribution from NB to either Poisson, ZIP or ZINB (or vices versa) and this information is summarized in Table S2b. It can be seen that around 50% to 75% of the genes switched distribution between a NB to Poisson distribution (or vice versa) in both pairwise comparisons for both tissues.

We next looked at what proportion of these genes were also detected as differentially expressed by either *edgeR*, *DESeq2* or *DEsingle*. Although there was not a high overlap between the differentially distributed genes detected by our pipeline and the differentially expressed genes detected by ~~*edgeR*~~ the other methods, we observed that most of the genes commonly detected by ~~these two methods~~ *scShapes* and *edgeR* or *DESeq2* switch distribution from NB to Poisson, ZIP or ZINB (or vice versa, Table S3a-b). Further investigation of the overlap of genes detected by *edgeR* or *DESeq2* and our *scShapes* framework revealed that most of these genes follow a unimodal distribution in the two pair-wise comparisons in both adipose and muscle (48% to ~~77~~93% of the genes, see Table ~~2~~S4a-b). This is to be expected since both *edgeR* and *DESeq2*

Formatted: Font: (Default) Times New Roman, 12 pt
Formatted: Font: (Default) Times New Roman, 12 pt
Formatted: Font: (Default) Times New Roman, 12 pt
Formatted: Line spacing: 1.5 lines
Formatted: Font: (Default) Times New Roman, 12 pt
Formatted Table
Formatted: Font: (Default) Times New Roman, 12 pt
Formatted: Line spacing: 1.5 lines
Formatted: Font: (Default) Times New Roman, 12 pt
Formatted: Line spacing: 1.5 lines
Formatted: Font: (Default) Times New Roman, 12 pt
Formatted: Font: (Default) Times New Roman, 12 pt
Formatted: Font: (Default) Times New Roman, 12 pt
Formatted: Line spacing: 1.5 lines
Formatted: Font: Italic
Formatted: Space After: 0 pt, Line spacing: single, Tab stops: 1.15", Left
Formatted: Line spacing: Multiple 1.15 li, Tab stops: 1.15", Left

detect~~s~~ differential expression by assuming that the gene expression levels follow a negative binomial distribution for all genes. Similarly, one would expect that genes detected to be both differentially distributed by *scShapes* and differentially expressed by *DEsingle* would follow a ZINB distribution. However, only in muscle tissue between old and treated did we observe around 56% of the differentially distributed genes to switch distribution from ZINB to Poisson, NB or ZIP (or vice versa, Table S3c). In all other comparisons only less than 50% of the genes that were both differentially distributed and differentially expressed switched distribution from ZINB to Poisson, NB or ZIP (or vice versa, Table S3c & Table S4c).

Formatted: Font: Italic

Formatted: Font: Italic

~~**Table 2: Break down of distribution shapes of genes that were detected to be both differentially distributed by scShapes and differentially expressed by edgeR**~~

Formatted: Line spacing: Double

Similarly, we also compared the distribution patterns of commonly differentially distributed genes that were detected by our DD pipeline and that of *scDD*. Compared to *edgeR*, there is little overlap between the genes detected by our pipeline and that of *scDD* in muscle, although this number is quite similar for adipose (33% and 15% in Old vs Young and Old vs Treated respectively, see Table ~~3~~2). Although both approaches are designed to detect differential distributions, they each make very different modeling assumptions about the distributions. In *scDD*, log-normalized gene expression measurements are modelled using a Bayesian non-parametric modelling framework utilizing Dirichlet process mixture models. The different assumptions that are made in *scDD* and *scShapes* mean that these methods are not equally affected by the degree of heterogeneity occurring from cell to cell in the adipose and muscle tissues, and hence different sets of genes were obtained. Nevertheless, we further investigated the genes identified by both *scShapes* and *scDD* to identify functional terms enriched in the

Formatted: Font: Italic

Formatted: Font: Italic

overlapping gene sets. We observed that Hallmark pathways such as *DNA repair* and *MTORC1 signalling* were enriched in adipose between both pairwise comparisons. Similarly in muscle we observed that the Hallmark pathways such as *adipogenesis* and *hypoxia* are over-represented in genes identified to be differentially distributed by both *scShapes* and *scDD* between old and young (Figure S4).

Formatted: Font: Italic

Formatted: Font: Italic

Formatted: Font: Italic

Formatted: Font: Italic

Formatted: Font: Italic

Formatted: Font: Italic

Of the genes that were commonly detected by *scShapes* and *scDD*, most of them were categorized either as DE or DZ (differential proportion of zeros) by *scDD*. For the genes which are not differentially distributed in the non-zero values, *scDD* checks whether the proportion of zeros are significantly different between the two conditions (DZ). We further investigated the pattern of distribution of the genes detected to be DD by our pipeline as well as classified as DZ by *scDD* (Table ~~S4~~S5). We checked whether these genes switched from a unimodal distribution to a zero-inflated distribution between conditions. However, less than 50% of the genes would switch distribution between a unimodal distribution and a zero-inflated distribution in the genes classified as DZ by *scDD*. This suggests that *scShapes* is detecting a different type of profile change in gene expression than what can be explained by *scDD*'s DZ criteria alone.

Table ~~3~~ 2: Investigating whether genes detected by *scShapes* share any similarities with distribution categories detected by *scDD*.

Formatted: Font: Italic

Formatted: Font: Italic

Tissue	Comparison	Overlap between DD genes from *scShapes* and *scDD*	Genes detected as DB by *scDD*	Genes detected as DE by *scDD*	Genes detected as DP by *scDD*	Genes detected as DZ by *scDD*
Adipose	Old vs Young	301	3	177	0	121
	Old vs Treated	112	2	47	0	63
Muscle	Old vs Young	51	0	12	0	39
	Old vs Treated	20	0	9	0	11

Additional case studies to demonstrate *scShapes*' utility for a wide range of experimental designs.

The utility of the *scShapes* framework can be best demonstrated by applying it in the context of multiple datasets that represent a diverse range of biological perturbations. The primary dataset used in this study describes one of the most common experimental designs used for scRNA-seq approaches where single cells are profiled from multiple treatment conditions, *e.g*. young, old, and treated mice. This design is illuminating for understanding ageing and metformin-induced effects on the transcriptome. It is important to recognize that other kinds of designs are represented in scRNA-seq datasets and that *scShapes* is able to infer insightful results for these too. We selected two other experimental designs to showcase the flexibility and utility of *scShapes* to identify useful biological results in the presence of different degrees of complexity present in scRNA-seq data.

Using *scShapes* to gain insights into the transcriptional response of the immune system in COVID-19 patients.

A scRNA-seq dataset was generated for peripheral blood mononuclear cells (PBMCs) from seven patients hospitalized for COVID-19 and six healthy controls [42] (Figure ~~S4a~~S5a). We selected this dataset to showcase how *scShapes* can detect differential distributions in the presence of a much higher number of cells (~44,721 cells versus ~12,987 in the previous ageing dataset) and make use of multiple covariates. In the original study, Wilk *et al*. [42] classified each cell in the COVID-19 patient data and healthy controls into one of 20 known cell types. Cell type membership, donor ID and gender were included in *scShapes* as explanatory covariates in the GLM. *scShapes* was used to determine the prevalence of genes classified into each of the four distributions Poisson, NB, ZIP and ZINB (Table ~~S5~~S6) and determine genes that switched distributions between the COVID-19 group and the healthy control group.

Interestingly, we found that over 80% of genes followed a NB distribution, with the number of genes Poisson distributed being negligible. Pathway over-representation analysis of the genes switching distribution between COVID-19 group and healthy controls revealed pathways like *oxidative phosphorylation*, *interferon response*, and *immune response* related pathways (Figure ~~S4~~ S5 b-d) which are all pathways previously implicated with COVID-19 [43, 44].

We also used this dataset to investigate the nature of differential distributions occurring within a specific cell type. We focused our attention on the seven main cell-types, B cells, natural killer (NK) cells, dendritic cells (DCs), CD4$^+$ T cells, CD8$^+$ T cells, CD14$^+$ monocytes and CD16$^+$ monocytes. We found that there was significant overlap between the differentially-distributed genes in NK cells and the T cells (Figure ~~S5a~~S6a). We also found that differentially-distributed genes were enriched for interferon (IFN) responses, inflammatory responses and NF-κB signaling pathway (Figure ~~S5b~~S6b). We also saw a statistically significant higher enrichment of *apoptosis*, metabolic pathway *oxidative phosphorylation* and *WNT/β-catenin signalling* between the differentially distributed genes between COVID-19 group and healthy controls in NK cells, CD4$^+$ T cells and CD8$^+$ T cells.

Pathway over-representation analysis of simulated T-cells highlights pathways implicated in T-cell activation

In contrast to the previous designs, we included a design that featured a single cell type that was profiled in different tissues. This kind of experimental design is relevant to scRNA-seq datasets because we have already shown that over-dispersion and zero-inflation observed in gene expression rates are influenced by the presence of different cell-types. A study generated scRNA-sequencing data of human T-cells isolated from bone marrow (BM), lungs (LG) and lymph node (LN) from two adult deceased organ doanors and blood (BL) from two healthy donors for comparison [45] (Figure ~~S6a~~S7a). Tissues acquired from the donors were stimulated

resulting in over 50,000 resting and activated T-cells. We used *scShapes* to model the gene expression distributions separately under the two treatment conditions, resting (non-stimulated) and activated (stimulated) where the donor ID was used as an explanatory covariate that was adjusted for in the GLM model. As expected, the Poisson distribution was the most prevalent distribution across the four tissue types in both treatment conditions. Interestingly we see that there were more genes with distributions that were classified as either NB, ZIP or ZINB distributed across stimulated T cells in all tissue types and blood (see Table ~~S6~~S7) compared to resting T cells. The genes that switched distributions between resting and activated (Table ~~S7~~S8) in the four tissue types were enriched for *activation of immune response* and other GO terms (see Figure ~~S6~~ S7 b-e). This result likely reflects the fact that in BM, LG and LN, the activated T-cells secrete cytokines that regulate immune response. The differentially distributed genes in this pathway that are shared between BM, LG and LN include *PSMC1*, *PSMC3* where both of these genes are part of the 26S proteasome involved in cell-cycle progression, DNA damage repair, apoptosis, and *XRCC6* involved in regulation of innate immune response [46].

Benchmark of *scShapes* computational needs

As *scShapes* estimates the parameters of each gene by maximum likelihood estimation (MLE) using the iterative weighted least squares (IWLS) algorithm, the computational cost of the framework is high. In order to get an evaluation of the time taken to run *scShapes* as a function of the number of cells, we utilized the Human PBMC daataset from [47]. This dataset comprised of 161,764 cells collected from three time points collected at day 0, 3 and 7 from eight volunteers. We ran *scShapes* on each time point with volunteer ID and cell type membership included as covariates in the GLM. The modelling and testing framework of *scShapes* can be summarized under three main steps; (a) KS test to identify the genes belonging to the family of ZINB distributions; (b) model fitting using the four distributions Poisson, NB,

Formatted: Font: (Default) Times New Roman, 12 pt, Bold

Formatted: Font: (Default) Times New Roman, 12 pt, Bold

Formatted: Normal, Justified, Line spacing: 1.5 lines

Formatted: Font: (Default) Times New Roman, 12 pt, Bold, Italic

Formatted: Font: 12 pt

Formatted: Normal, Justified

Formatted: Font: (Default) Times New Roman, Italic

Formatted: Font: (Default) Times New Roman, 12 pt

Formatted: Font: (Default) Times New Roman, Italic

Formatted: Font: (Default) Times New Roman, Italic

Formatted: Font: (Default) Times New Roman, Italic

ZIP and ZINB; (c) goodness of fit tests to identify the best model for each gene. Hence, the computational needs of *scShapes* were also benchmarked under the above three main categories. It is evident that the computational demand for especially for running the KS test is non-negligible. This is mainly due to fitting ZINB distributions by estimating the unknown parameters from sample estimates with P-values calculated from bootstrapped Monte Carlo estimates, both of which are computationally expensive.

Formatted: Font: (Default) Times New Roman, Italic

Hence, we tested whether pre-filtering for zero-inflation or by down sampling the cell numbers, the computational cost could be reduced. For this purpose we utilized a test to check for zero-inflation in count models to test whether a Poisson model is under fitting zeros in the observed variable (see Methods). Then the zero-inflated distributions (ZIP and ZINB) will only be fitted to the genes identified to be under fitted by a Poisson distribution. By introducing the test for zero-inflation (ZI), the average computational time taken for fitting the four distributions, reduced by around 67% (Figure 6b). Whilst the distributions Poisson, NB and ZINB were accurately identified with an accuracy of over 91% after introducing the zero-inflation test (Figure 6a), there was a drop in accuracy when identifying the genes following a ZIP distribution (accuracy = 67%).

We next tested, the impact of cell numbers and cell proportions in accurately identifying the distributions as well as on the computational demands. For this purpose, we followed three main strategies where we first randomly down sampled the total cells to 8000 cells (Random) with each cell type having equal number of cells each (1000 cells each) and another iteration where we maintained the original cell proportions (Proportion). Finally, instead of randomly sampling cells we subsampled cells in a density dependent manner (Kernel) as previously done by [48] with 1000 cells in each cell type. It is evident that by down sampling the computational time has drastically reduced by around 77% compared to running *scShapes* on the entire dataset (Figure 6b). Overall, we can see that each of the subsampling techniques achieved over 70%

Formatted: Font: (Default) Times New Roman, Italic

accuracy except the Proportion method when identifying the NB distribution (65% accuracy) and the Kernel method in identifying the ZIP distribution (Figure 6a). However, it is noteworthy that of the total genes, less than 1% of the genes in the PBMC dataset were identified to be following to be following a ZIP distribution.

Finally we benchmarked the computational time required by *edgeR*, *DESeq2*, *DEsingle* and *scDD* for identifying DE or DD genes in the Human PBMC dataset. The calculations of *scDD* aborted because it ran out of memory when performing the fisher's exact test. Both *edgeR* and *DESeq2* were run by including the volunteer ID and cell type membership as covariates in the GLM. However, *DEsingle* does not facilitate the inclusion of covariates in its modelling framework. It can be seen that only *DEsingle* which models the count data using ZINB distributions required around 90 hours on average to identify the DE genes (Figure 6c). All computations were performed on a high performance computing cluster with 250 GM of RAM and with parallel computing using 6 nodes (except in *edgeR* which did not facilitate parallel computation).

Discussion

We have presented a flexible statistical framework for quantifying gene expression heterogeneity of scRNA-sequencing data by modelling gene expression levels under a differential distribution pipeline. What makes our method unique is that it is fundamentally different from a method that detects differential expression by testing for the significance of change in mean of the expression rates. Our framework identifies and classifies genes according to their shape of gene expression distribution and allows for the possibility that one gene's expression profile across a population of single cells may be different from another gene. As scRNA-seq data are characterized by properties of sparsity and high dimensionality,

Formatted: Font: (Default) Times New Roman, Italic
Formatted: Font: (Default) Times New Roman, Italic
Formatted: Font: (Default) Times New Roman, Italic
Formatted: Font: (Default) Times New Roman, Italic
Formatted: Font: (Default) Times New Roman, Italic
Formatted: Font: (Default) Times New Roman, Italic
Formatted: Font: (Default) Times New Roman, Italic
Formatted: Font: (Default) Times New Roman, Italic
Formatted: Font: (Default) Times New Roman, Italic
Formatted: Font: (Default) Times New Roman, Italic
Formatted: Font: 12 pt
Formatted: Line spacing: 1.5 lines

our framework uses a special class of statistical distributions that can handle an excess degree of zeros. Recently, there has been discussion in the literature regarding the use of zero-inflated distributions for modelling single cell transcriptomic data. It is argued in Cao et al. [49] that zero-inflation is supressed in gene expression counts measured in terms of UMI counts, and in Svensson [50] that a NB distribution alone is sufficient to model the zero counts in droplet-based scRNA-sequencing data. However, this study [50] models the gene expression heterogeneity of negative control (e.g. ERCC spike-ins) droplet-based scRNA-seq data. As negative control data lack biological variation a NB distribution may sufficiently model such gene expression measurements and hence there remains motivation to model the excess zeros of at least a subset of the genes. -Although the focus of this study has been based on scRNA-sequencing data generated through droplet-based methods, we show that zero-inflation is required for a significant number of genes to ensure the best distribution fit depending on the nature of the experiment, because around 5% to 20% of the genes under different experimental conditions were detected to follow a zero-inflated distribution. Our study also validates the fact that zero-inflation may be interpreted from a biological perspective i.e. based on cell type membership, which is in line with the findings of Choi *et al*. [18] and Clivio *et al*. [33], and not completely a result that is just an artefact of technical variability.

When compared to other popular tools for identifying differential distributions like *scDD* [12], it is valuable to acknowledge that the *scShapes* approach has the flexibility to account or adjust for multiple confounders in the form of covariates in the GLM. Our approach does not require that prior normalization be done for nuisance factors, but rather enables model-based normalization using the GLM. Furthermore, our method also has the flexibility to perform multiple comparisons across biological conditions and is not restricted to only pair-wise comparisons. This feature provides the option to investigate both global (e.g. effects treatment

groups) and pairwise changes (e.g. young versus old), similar to an ANOVA test followed by a post-hoc pairwise comparison but for assessing differential distributions.

One of the tools that exists for identifying the most appropriate statistical model is *M3S* [51], which selects the best fit model from a pool of 11 models using a Kolmogorov-Smirnov test. The 11 distributions considered by *M3S* includes the four distributions included in our *scShapes* method and additional statistical distributions such as Gaussian mixture models. However, we believe that the use of Gaussian distributions to model count data is not advisable as we run the risk of losing information by fitting continuous distributions to count data. Additionally, the use of 11 different distributions makes it more challenging to biologically interpret the best fit distribution for each gene. Hence, for simplicity and efficiency, the four distributions Poisson, NB, ZIP and ZINB represent a sufficient selection for modelling scRNA-seq data.

scRATE is another recently published tool for modelling droplet-based scRNA-seq data that uses a mix of unimodal and zero-inflated distributions[18]. This method relies on Bayesian model selection to identify the best model for each gene, and is similar to our method as it also models gene expression distributions using Poisson, NB, ZIP and ZINB distributions using a GLM with an offset term to account for differences in sequencing depth. Importantly, where this method differs from our approach is in its handling of model selection, which is based on the expected log predictive density (ELPD) score. Also the main focus of this study was to identify the origins of zero-inflation rather than to detect differentially distributed genes. Since the key objective of our approach is to detect differential distributions for the purposes of understanding biological perturbations, we ensure that the best fit distribution is identified as appropriate to each gene from the pool of four distributions considered by first performing a Kolmogorov-Smirnov test to identify genes belonging to the family of ZINB distributions. Only the genes that pass through the KS test, would then be considered for the four distributions Poisson, NB, ZIP and ZINB to identify the distribution of best fit. Once model selection is done

using the BIC value, we also perform additional model adequacy tests such as testing for the presence of zero-inflation in the genes detected to be following a zero-inflated distribution to ensure that the model distribution is identified correctly and appropriately. The use of the KS test together with the model adequacy tests are crucial since our primary focus is identifying genes that switch distribution shape. Whilst *scRATE* also employs a GLM based modelling framework with Bayesian model selection, we developed our own modelling framework scShapes which utilizes maximum likelihood estimation (MLE) as Bayesian estimation is best suited for data with prior knowledge about the distribution it originated from. –It is also noteworthy that both the tools *M3S* and *scRATE* fail to first subset genes that belong to the family of statistical distributions under study, but rather assume that all the genes would follow one of the distributions from the pool of distributions considered. Our study, as well as others, present compelling evidence that this assumption is limited or incorrect, especially for scRNA-seq data.

Formatted: Font: Italic

Compared to standard methods of DE, which aim to detect a shift in the mean of gene expression data such as *edgeR*, *DESeq2* or *DEsingle* our method does not make the assumption that all the genes in the transcriptome follow a single distribution. While these methods perform well at detecting statistically significant differences in mean between conditions, they fail to detect subtle changes in gene expression levels that do not involve a change in mean. In addition, even though the negative binomial distribution is a flexible distribution that is capable of handling over-dispersion in the data, it lacks the flexibility to explicitly model both structural zeros and random zeros in the data. As shown by Choi *et al*. [18], the zeros we observe in scRNA-seq data are biological in nature and not purely produced by technical artefacts. However, a negative binomial distribution assumes that the zeros are a result of random sampling and does not allow for the recognition of true biological zeros in the data. This assumption that is made by the NB distribution goes against one of the core truths in biology

Formatted: Font: Italic

Formatted: Font: Italic

and one of the key strengths of single cell profiling techniques. Hence it is vital that we model gene expression distributions at the gene level to allow for both random zeros and structural zeros in the data to be identified.

Our framework *scShapes* is built on the assumption that, once all sources of variation other than true biological variation are accounted for, the change in distribution we see is due to the phenotypic changes under consideration in the experimental study. Furthermore, our framework does not aim to cluster cells into subtypes as genes are modelled and evaluated independently. We acknowledge that while this framework is computationally expensive and can be time consuming, especially when applied to datasets with large number of cells, it is possible that improvements through parallelization or more advanced usage of high performance computer clusters~~sing~~ could address this limitations. By down sampling from a larger PBMC dataset, we showcased that *scShapes* was able to correctly detect the distribution with an overall accuracy of around 65% or more regardless of the sampling scheme or cell type proportions, even with only around 15% (8000/53921 cells) of the total cells. This provides a promising opportunity to address the computational demands of *scShapes* in future.

Formatted: Font: Italic

Formatted: Font: Italic

Some of the genes and pathways discovered through our framework overlap with those identified through methods of transcriptomic data where standard assumptions have been made, indicating the framework's ability to identify known markers. Importantly this method is able to identify additional genes and pathways both at the tissue level and cell specific level, which may point to new knowledge such as in the example of aging. Most importantly the *scShapes* framework has the flexibility to adjust for any number of covariates (e.g. batch effects, cell-types etc.) and perform multiple comparisons across any set biological conditions of interest. Hence modelling a gene's expression distribution shape at the gene level, provides an opportunity to extract precise genes and pathways not necessarily put forth by traditional methods of DE.

Methods

Code Availability

We have implemented our model selection pipeline as an open-source R package, *scShapes*, available from Bioconductor for Linux, Windows and MacOS. The source code is available on github.com/Malindrie/scShapes under a GPL-3 license. Documentation on the functions and examples for *scShapes* are also available on our GitHub repository. Using *scShapes* requires an input of a matrix of UMI counts with genes as rows and cells as UMIs, along with a data frame of covariates to be included in the GLM and a numeric vector of library sizes. The library sizes are calculated as the total UMI counts per cell, which will be used as an offset in the GLM to account for differences in the sequencing depth. We recommend only a few genes be fitted on a local machine due to the computational demand. Hence, we have containerized the software using both docker and singularity to facilitate its use on high-performance computing clusters or with cloud computing. All analysis scripts used in the paper can be accessed at https://github.com/Malindrie/paper-scShapes.

Methodology

In order to ensure that only expressed genes are included in the analysis, we retained genes expressed (defined as having a read count > 0) in at least 10% of all cells under each treatment condition. We then restricted our attention to the subset of genes common between all three conditions for downstream analysis. The UMI counts for a given gene are modelled using generalized linear models (GLMs) under each condition separately. The sum of all counts in

each cell is used as a proxy to normalize the cells for sequencing depth differences. This cell attribute is used as an offset in a regression model along with any other covariates to account for known sources of cell-to-cell variability. The four models being compared are the Poisson (P), Negative Binomial (NB), Zero Inflated Poisson (ZIP) and Zero Inflated Negative Binomial (ZINB), which are special cases of one another, and all belong to the family of ZINB distributions. Hence, only the subset of genes belonging to the ZINB family of distributions is selected via a Kolmogorov–Smirnov (KS) goodness of fit test, with P-values computed through Monte Carlo simulation. For the significant genes from the KS test under each condition, the expression level of a given gene is then modelled using the error distributions Poisson, NB, ZIP and ZINB with a log link function.

Parameter Estimation

For model fitting and parameter estimation, the R package **pscl** [52] which allows for the fitting of zero-inflated models was used. The two unimodal distributions, Poisson and NB are implemented in R by the *glm* function [53] in the **stats** package and the *glm.nb* function [54] in the **MASS** package respectively. The package **pscl** provides an extension over the existing R packages for fitting GLM, where the regression coefficients are estimated by maximum likelihood estimation (MLE) using the iterative weighted least squares (IWLS) algorithm.

The parameter estimates are obtained by maximizing the likelihood or log likelihood of the parameters for the observed data. The log-likelihood for the observation y given the parameterθ, expressed as a function of the mean-value parameter, $\mu = E(Y)$ is given by;

$$l(\mu; y) = \log f(y; \theta)$$

where $f(y; \theta)$ is the density function of y. The log-likelihood based on a set of independent observations $y_1, \ldots, y_n$ is the sum of the individual contributions so that,

$$l(\mu; y) = \sum_i \log f_i(y_i; \theta_i)$$

where $\mu = \mu_1, \ldots, \mu_n$

Model Selection

A critical step in the model building process is the selection of the model that best fits the data among a set of competing models. Among numerous criteria available for model selection, we decided to select the best model based on the least Bayesian Information Criterion (BIC) value as the BIC generally introduces a higher penalty for models with a more complicated parametrization and BIC [55] values are calculated as follows;

$$\text{BIC} = -2 . \ln(\hat{L}) + k . \ln(n)$$

where k is the number of free parameters to be estimated, $\hat{L}$ is the maximum value of the likelihood of the model, and n is the number of observations.

However, most applications in the literature use different diagnostics in conjunction with information criterion to arrive at the model that best fits the data. Hence further model adequacy tests were done to validate the models selected based on the least BIC value. The deviance goodness of fit test assess the adequacy of the model by comparing the fitted model with the fitted saturated model. The deviance statistic D, also known as the likelihood ratio statistic is defined as;

$$D = 2[l_s(\hat{\psi}) - l(\hat{\beta})]$$

where $\hat{\psi}$ and $\hat{\beta}$ are the MLEs of the saturated and fitted model respectively. The deviance statistics is asymptotically chi-squared distributed with $K - p$ degrees of freedom, where K and p are the number of parameters in the saturated and fitted models respectively [56].

i.e.

$$D \sim \chi^2_{K-p}$$

However, the deviance goodness of fit test cannot be used to test the adequacy of the zero-inflated models as these models are not strictly nested i.e. the smaller model sits on the boundary of the parameter space of the larger model [57]. Conversely Molenberghs and Verbeke [17] showed that the resulting likelihood ratio (LR) test statistic of the form;

$$\varrho = -2\,[l(\hat{\beta}^{(r)}, \hat{\phi}^{(r)}, 0) - l((\hat{\beta}, \hat{\phi}, \widehat{\omega}))]$$

where ω is the zero inflation parameter and $l(\hat{\beta}^{(r)}, \hat{\phi}^{(r)}, 0)$ the LR of the model with all parameters of the model estimated with the restriction $\omega = 0$, follows an equal mixture of a chi-squared distribution with 0 degrees of freedom and a chi-squared distribution with 1 degree of freedom [57]. i.e.

$$\varrho \sim (0.5)\chi^2_0 + (0.5)\chi^2_1$$

Hence we test for the presence of zero-inflation in the zero-inflated models selected based on the least BIC value using the above test.

Identifying Differentially Distributed Genes

Each gene is modelled using a GLM where the read count y_{ij} for gene i in cell j is assumed to follow one of the following distributions, namely, Poisson, NB, ZIP or ZINB under each biological condition. After performing model adequacy tests detailed above, the distribution that best fits for each gene is identified. Once the statistical distribution for each gene is identified, comparisons in distribution shapes between biological conditions are carried out, to identify genes switching distribution shape between treatment conditions.

Test for zero-inflation

Formatted: Font: Bold

In order to speed up the modelling process of *scShapes*, we utilized an additional test for zero-inflation. First the GLM with Poisson error distribution is fitted for each gene (after filtering) and next it is tested whether this predicted model is under fitting the zeros compared to the observed zeros. For this purpose we utilized the *check_zeroinflation* function from the performance (version 0.9.0) R package. Briefly, this test compares the ratio of observed and predicted zeros. A ratio between 1 +/- tolerance (= 0.05) is considered as a well fit model. Hence only genes with a ratio above or below this threshold were subsequently fitted the zero-inflated distributions.

Formatted: Font: Italic

Formatted: Font: Italic

Datasets used

Metformin mouse dataset

The scRNA-seq metformin dataset was collected from mice on the two tissues, adipose and muscle. In the study design, three groups of mice, each group consisting of four male C57BL/6J mice, with the young group composed of 3-4 months old mice, old group composed of 18-19 months old mice and treated group composed of 18-19 months old mice treated with 1000ppm (0.1% w/w) metformin for 6 weeks [58]. Using single-cell RNA-Seq, the transcriptome of ~13000 cells from adipose stromal-vascular fraction and ~5000 cells from skeletal muscle were sequenced. The dataset was sequenced on 10X Chromium scRNA-seq platform. We used the pre-processed UMI counts, obtained using Cellranger version 1.3 (10X Genomics). Downstream analysis using Seurat version 3.0.0 [59] identified 12 known cell types in adipose and 13 known cell types in muscle.

Simulated T cells dataset

Human T-cells of >50,000 resting and activated cells have been isolated from mucosal and lymphoid sites of two adult deceased organ donors and blood from two healthy donors [45]. The scRNA-seq data were downloaded from the Gene Expression omnibus (GEO) under

accession number GSE126030 ~~[https://www.ncbi.nlm.nih.gov/geo/query/acc.cgi?acc=GSE126030]~~. Similar to the metformin dataset, we applied filters to retain only genes expressed in at least across 10% of the cells to ensure we only included genuinely expressed genes.

Peripheral blood mononuclear cells (PBMCs) from patients hospitalized for COVID-19

This dataset consists of scRNA-sequencing data of peripheral blood mononuclear cells (PBMCs) from seven patients hospitalized for COVID-19 (male patients) and six healthy controls (4 male and 2 female) [42]. The scRNA-seq data of PBMCs from the COVID-19 patients were downloaded from the Gene Expression omnibus (GEO) under accession number GSE150728~~We used the processed count matrices downloaded from the COVID-19 Cell Atlas (https://www.covid19cellatlas.org/#wilk20) along with the de-identified metadata and embedding~~. We used information on the 20 cell-types annotated along with the patient ID and gender as covariates in our modelling framework.

Peripheral blood mononuclear cells (PBMCs) from human volunteers

The scRNA-sequencing data of peripheral blood mononuclear cells (PBMCs) from eight volunteers enrolled in an HIV vaccine trial from the three time points at day 0, 3, and 7 following vaccination [47]. The data were downloaded from the Gene Expression omnibus (GEO) under accession number GSE164378. We used information on the 8 cell-types annotated along with the volunteer ID as covariates in our modelling framework.

Simulation study for testing the differential distribution pipeline.

To get a realistic set of model parameters, we first performed model classification using our pipeline on the 3k-cell PBMC dataset to learn parameters of the four distributions for each

gene. The 3k PBMCs were blood samples from one healthy donor generated using the v1. chemistry and preprocessed with CellRanger 1.1.0. [60] (https://support.10xgenomics.com/single-cell-gene-expression/datasets/1.1.0/pbmc3k). We then generated UMI counts using parameters estimated for each gene by sampling a total cell UMI count from the input data. Using the model classification and parameter estimation we simulated count data for three sample sizes, 2638, 3k and 5k cells. The scRNA-seq counts were generated using the R functions *rpois, rnegbin, rzipois, rzinegbin* available through the R packages **stats**, **MASS** and **VGAM** respectively. We then applied our framework on the simulated data and checked for summary statistics such as accuracy, sensitivity and specificity.

Accuracy of the simulation study reflects the ability of *scShapes* to differentiate between the four different distributions considered. This is calculated as:

$$\text{Accuracy} = \frac{\text{TP+TN}}{\text{TP+TN+FP+FN}}$$

Sensitivity of the simulation study reflects the ability of *scShapes* to determine the correct distribution or the true positives. This is calculated as:

$$\text{Sensitivity} = \frac{\text{TP}}{\text{TP+FN}}$$

Specificity of the simulation study reflects the ability of *scShapes* to determine the true negatives. This is calculated as:

$$\text{Specificity} = \frac{\text{TN}}{\text{TN+FP}}$$

Where for a given gene which is known to be following a Poisson distribution

True positive (TP) = the number of cases correctly identified as Poisson

False positive (FP) = the number of cases incorrectly identified as Poisson

True negative (TN) = the number of cases correctly identified as NB, ZIP, ZINB

False negative (FN) = the number of cases incorrectly identified as NB, ZIP, ZINB

Evaluating the significance of overlapping gene sets

To test the significance of overlapping sets of genes, when applying *scShapes* with and without accounting for known sources of biological variability, we used the R package *SuperExactTest* [61], both for calculating the significance of overlap between the gene sets and for visualizations.

Pathway over-representation analysis

Pathway over-representation analysis was done using the MSigDB – GSEA online tool by the Broad Institute (https://www.gsea-msigdb.org/gsea/msigdb). This tool calculates the overlap between user provided gene sets and that of the MSigDB collection of datasets chosen by the user. An estimate of the statistical significance of the overlapping gene set is provided based on the hypergeometric P-value which is corrected for multiple hypothesis testing using Benjamini and Hochberg method. All overlaps significant at *p-value* < 0.05 were selected with visualization of top significant pathways. Visualization of the significant pathways was done using the package *ggplot2* in R version 4.0.2.

Methods comparison between scShapes, edgeR and scDD

edgeR [37] uses a negative binomial generalized linear model for differential expression testing. The quasi-likelihood pipeline using the function *glmQLFTest* implemented in *edgeR* was used for identifying the differentially expressed genes in the pair-wise comparisons of interest, as this method allows for stricter error control than other pipelines of *edgeR*. Lowly

expressed genes were filtered (expressed in less than 10% of the cells) and the mouse ID and cellular detection rate (average number of genes expressed per cell) used as covariates to account for the differences in the mice and sequencing depth respectively. Genes were corrected using the Benjamini & Hochberg (BH) correction and only genes that had a corrected *P-value* of < 0.01 were considered to be significantly differentially expressed.

DESeq2 [38] facilitates the study of RNA-seq data where the estimates of dispersion and fold change are computed using shrinkage estimation. *DESeq2* also implements model-based normalization where it normalizes for the differences in sequencing depth between samples using the median-of-ratios method. Read counts are then modelled as following a NB distribution using GLM with a logarithmic link. In order to minimize the effect of noisy estimates on differential expression testing resulting from highly variable dispersion parameter estimates, information is shared across all the genes when estimating the dispersion parameter.

Formatted: Font: Italic

Formatted: Font: Italic

DEsingle [39] utilizes a zero inflated negative binomial (ZINB) model to estimate the proportion of real and dropout zeros. Parameter estimation of the ZINB model is done using constrained Maximum Likelihood Estimation (MLE) of the two ZINB populations. *DESingle* utilizes the likelihood ratio test for differential expression analysis and classifies the DEG into 3 types: 1 – DEs: different expression status, 2 – DEa: differential expression abundance, 3 – DEg: general differential expression. DEs refers to the genes showing a significant change in the percentage of zeros only between the two groups. DEa refers to the DEG between the groups, but do not have significant percentage of zeros between the two groups. Finally, DEg refers to the genes which are both differentially expressed (DE) between the groups and also have significant percentage of zeros between the groups.

Formatted: Font: Italic

Formatted: Font: Italic

scDD [12] utilizes a Bayesian modelling scheme to determine genes with differential distributions classifying them into one of four categories, differential unimodal (DU),

differential modality (DM), differential proportion (DP); DB (both DM and DU). Similarly lowly expressed genes were filtered and scran Normalization implemented using the *preprocess* function from *scDD*. Based on the output from the function *scDD* genes falling into one of the four categories were considered to be differentially distributed.

Assessing the prevalence of housekeeping genes

To overlap genes with housekeeping genes, we used a previously published list of ubiquitous genes from He *et al*. (2020) [19] downloaded from https://github.com/brianpenghe/Matlab-genomics/blob/master/He_2020_ENCODE3_RNA/GeneLists/Bulk%20Cluster%20Ubiquitous.txt.

Sampling cells in a density dependent manner

During the benchmarking of scShapes' computational needs, one sampling strategy we followed was to sample cells in a density dependent manner as previously followed by [48]. Briefly, 1000 cells from each cell type are subsampled such that the probability of selecting a cell c is $\frac{1}{d(\log_{10} N_c)}$, where d is the density estimate of $\log_{10}$(total cell UMIs) and N_c the total UMI counts in cell c.

Abbreviations

ANOVA: Analysis of variance

BH: Benjamini & Hochberg

BIC: Bayesian Information Criterion

BL: Blood

BM: Bone marrow

DB: Both differential modality and different component means

DC: Dendritic cells

DD: Differential distribution

DE: Differential expression

DM: Differential modality

DP: Differential proportion

DZ: Differential zeroes

ELPD: Expected log predictive density

GLM: Generalized linear model

KS: Kolmogorov-Smirnov

LG: Lungs

LN: Lymph node

LRT: Likelihood ratio test

NB: Negative binomial

NK: Natural killer

PBMC: Peripheral blood mononuclear cells

QLF: Quasi-likelihood approach

scRNA-seq: Single-cell RNA sequencing

ZINB: Zero inflated negative binomial

ZIP: Zero inflated Poisson

References

1. Buettner, F., et al., *Computational analysis of cell-to-cell heterogeneity in single-cell RNA-sequencing data reveals hidden subpopulations of cells.* Nature Biotechnology, 2015. **33**(2): p. 155-160.
2. Mar, J.C., *The rise of the distributions: why non-normality is important for understanding the transcriptome and beyond.* Biophys Rev, 2019. **11**(1): p. 89-94.
3. Nguyen, A., et al., *Single Cell RNA Sequencing of Rare Immune Cell Populations.* Frontiers in Immunology, 2018. **9**(1553).
4. Jackson, C.A., et al., *Gene regulatory network reconstruction using single-cell RNA sequencing of barcoded genotypes in diverse environments.* Elife, 2020. **9**.
5. Cuomo, A.S.E., et al., *Single-cell RNA-sequencing of differentiating iPS cells reveals dynamic genetic effects on gene expression.* Nature Communications, 2020. **11**(1): p. 810.
6. Kharchenko, P.V., L. Silberstein, and D.T. Scadden, *Bayesian approach to single-cell differential expression analysis.* Nature Methods, 2014. **11**(7): p. 740-742.
7. Shalek, A.K., et al., *Single-cell transcriptomics reveals bimodality in expression and splicing in immune cells.* Nature, 2013. **498**(7453): p. 236-240.
8. Torrenté, L.d., et al., *The shape of gene expression distributions matter: how incorporating distribution shape improves the interpretation of cancer transcriptomic data*. 2019, bioRxiv.
9. Finak, G., et al., *MAST: a flexible statistical framework for assessing transcriptional changes and characterizing heterogeneity in single-cell RNA sequencing data.* Genome Biology, 2015. **16**(1): p. 278.
10. Chen, Y., et al., *edgeR: differential expression analysis of digital gene expression data.* 2019.
11. Love, M.I., W. Huber, and S. Anders, *Moderated estimation of fold change and dispersion for RNA-seq data with DESeq2.* Genome Biology, 2014. **15**(12): p. 550.
12. Korthauer, K.D., et al., *A statistical approach for identifying differential distributions in single-cell RNA-seq experiments.* Genome Biology, 2016. **17**(1): p. 222.
13. L. Lun, A.T., K. Bach, and J.C. Marioni, *Pooling across cells to normalize single-cell RNA sequencing data with many zero counts.* Genome Biology, 2016. **17**(1): p. 75.
14. Wagner, A., A. Regev, and N. Yosef, *Revealing the vectors of cellular identity with single-cell genomics.* Nature Biotechnology, 2016. **34**(11): p. 1145-1160.
15. Tanay, A. and A. Regev, *Scaling single-cell genomics from phenomenology to mechanism.* Nature, 2017. **541**(7637): p. 331-338.
16. Larsson, A.J.M., et al., *Genomic encoding of transcriptional burst kinetics.* Nature, 2019. **565**(7738): p. 251-254.
17. Molenberghs, G. and G. Verbeke, *Likelihood Ratio, Score, and Wald Tests in a Constrained Parameter Space.* The American Statistician, 2007. **61**(1): p. 22-27.
18. Choi, K., et al., *Bayesian model selection reveals biological origins of zero inflation in single-cell transcriptomics.* Genome Biology, 2020. **21**(1): p. 183.
19. He, P., et al., *The changing mouse embryo transcriptome at whole tissue and single-cell resolution.* Nature, 2020. **583**(7818): p. 760-767.
20. Meng, G. and H. Mei, *Transcriptional Dysregulation Study Reveals a Core Network Involving the Progression of Alzheimer's Disease.* Frontiers in aging neuroscience, 2019. **11**: p. 101-101.
21. Gorgoulis, V., et al., *Cellular Senescence: Defining a Path Forward.* Cell, 2019. **179**(4): p. 813-827.
22. Acoba, M.G., et al., *The mitochondrial carrier SFXN1 is critical for complex III integrity and cellular metabolism.* Cell Reports, 2021. **34**(11): p. 108869.
23. López-Otín, C., et al., *The hallmarks of aging.* Cell, 2013. **153**(6): p. 1194-1217.
24. Kulkarni, A.S., S. Gubbi, and N. Barzilai, *Benefits of Metformin in Attenuating the Hallmarks of Aging.* Cell Metab, 2020. **32**(1): p. 15-30.
25. Lei, Y., et al., *Metformin targets multiple signaling pathways in cancer.* Chinese Journal of Cancer, 2017. **36**(1): p. 17.

26. Wu, N., et al., *Metformin induces apoptosis of lung cancer cells through activating JNK/p38 MAPK pathway and GADD153.* Neoplasma, 2011. **58**(6): p. 482-90.
27. Hartwig, J., et al., *Metformin Attenuates ROS via FOXO3 Activation in Immune Cells.* Frontiers in immunology, 2021. **12**: p. 581799-581799.
28. Martins, R., G.J. Lithgow, and W. Link, *Long live FOXO: unraveling the role of FOXO proteins in aging and longevity.* Aging Cell, 2016. **15**(2): p. 196-207.
29. Ma, X., et al., *The nuclear receptor RXRA controls cellular senescence by regulating calcium signaling.* Aging Cell, 2018. **17**(6): p. e12831.
30. Kulkarni, A.S., et al., *Metformin regulates metabolic and nonmetabolic pathways in skeletal muscle and subcutaneous adipose tissues of older adults.* Aging Cell, 2018. **17**(2): p. e12723.
31. Lahoute, C., et al., *Premature aging in skeletal muscle lacking serum response factor.* PLoS One, 2008. **3**(12): p. e3910.
32. Kim, T.K., et al., *Interferon regulatory factor 3 activates p53-dependent cell growth inhibition.* Cancer Lett, 2006. **242**(2): p. 215-21.
33. Clivio, O., et al., *Detecting Zero-Inflated Genes in Single-Cell Transcriptomics Data.* bioRxiv, 2019: p. 794875.
34. Nikopoulou, C., S. Parekh, and P. Tessarz, *Ageing and sources of transcriptional heterogeneity.* Biol Chem, 2019. **400**(7): p. 867-878.
35. Rezaei-Lotfi, S., N. Hunter, and R.M. Farahani, *β-Catenin: A Metazoan Filter for Biological Noise?* Front Genet, 2019. **10**: p. 1004.
36. Kumar, R.M. and J.J. Collins, *Making a noisy gene: HDACs turn up the static.* Mol Cell, 2012. **47**(2): p. 151-3.
37. Robinson, M.D., D.J. McCarthy, and G.K. Smyth, *edgeR: a Bioconductor package for differential expression analysis of digital gene expression data.* Bioinformatics, 2010. **26**(1): p. 139-40.
38. Love, M.I., W. Huber, and S. Anders, *Moderated estimation of fold change and dispersion for RNA-seq data with DESeq2.* Genome Biol, 2014. **15**(12): p. 550.
39. Miao, Z., et al., *DEsingle for detecting three types of differential expression in single-cell RNA-seq data.* Bioinformatics, 2018. **34**(18): p. 3223-3224.
40. Soneson, C. and M.D. Robinson, *Bias, robustness and scalability in single-cell differential expression analysis.* Nature Methods, 2018. **15**(4): p. 255-261.
41. Andrawus, M., et al., *The effects of environmental stressors on candidate aging associated genes.* Experimental Gerontology, 2020. **137**: p. 110952.
42. Wilk, A.J., et al., *A single-cell atlas of the peripheral immune response in patients with severe COVID-19.* Nature Medicine, 2020. **26**(7): p. 1070-1076.
43. Chernyak, B.V., et al., *COVID-19 and Oxidative Stress.* Biochemistry. Biokhimiia, 2020. **85**(12): p. 1543-1553.
44. da Silva, R.P., et al., *Circulating Type I Interferon Levels and COVID-19 Severity: A Systematic Review and Meta-Analysis.* Frontiers in Immunology, 2021. **12**(1717).
45. Szabo, P.A., et al., *Single-cell transcriptomics of human T cells reveals tissue and activation signatures in health and disease.* Nature Communications, 2019. **10**(1): p. 4706.
46. Safran, M., et al., *GeneCards Version 3: the human gene integrator.* Database (Oxford), 2010. **2010**: p. baq020.
47. Stuart, T., et al., *Comprehensive Integration of Single-Cell Data.* Cell, 2019. **177**(7): p. 1888-1902.e21.
48. Choudhary, S. and R. Satija, *Comparison and evaluation of statistical error models for scRNA-seq.* Genome Biology, 2022. **23**(1): p. 27.
49. Cao, Y., et al., *UMI or not UMI, that is the question for scRNA-seq zero-inflation.* Nature Biotechnology, 2021. **39**(2): p. 158-159.
50. Svensson, V., *Droplet scRNA-seq is not zero-inflated.* Nature Biotechnology, 2020. **38**(2): p. 147-150.

51. Zhang, Y., et al., *M3S: a comprehensive model selection for multi-modal single-cell RNA sequencing data.* BMC Bioinformatics, 2019. **20**(24): p. 672.
52. Zeileis, A., C. Kleiber, and S. Jackman, *Regression Models for Count Data in R.* 2008, 2008. **27**(8): p. 25.
53. Chambers, J., T. Hastie, and D. Pregibon. *Statistical Models in S*. 1990. Heidelberg: Physica-Verlag HD.
54. Venables, W.N. and B.D. Ripley, *Modern Applied Statistics with S*. 2010: Springer Publishing Company, Incorporated.
55. Schwarz, G., *Estimating the Dimension of a Model.* The Annals of Statistics, 1978. **6**(2): p. 461-464.
56. McCullagh, P. and J.A. Nelder, *Generalized Linear Models, Second Edition*. Chapman and Hall/CRC Monographs on Statistics and Applied Probability Series. 1989: Chapman and Hall.
57. Wilson, P. and J. Einbeck, *A new and intuitive test for zero modification.* Statistical Modelling, 2019. **19**(4): p. 341-361.
58. Kulkarni, A.S., *Metformin modulates aging in a cell-type-specific manner in mouse muscle and adipose.* Manuscript in preparation.
59. Butler, A., et al., *Integrating single-cell transcriptomic data across different conditions, technologies, and species.* Nature Biotechnology, 2018. **36**(5): p. 411-420.
60. Zheng, G.X.Y., et al., *Massively parallel digital transcriptional profiling of single cells.* Nature Communications, 2017. **8**(1): p. 14049.
61. Wang, M., Y. Zhao, and B. Zhang, *Efficient Test and Visualization of Multi-Set Intersections.* Scientific Reports, 2015. **5**(1): p. 16923.

Acknowledgements

The authors like to thank Dr Alan Huang at the School of Mathematics and Physics, The University of Queensland for the constructive feedback given on the *scShapes* framework.

Funding

This work is supported by an Australian Research Council Future Fellowship (FT170100047) and a Georgina Sweet Award to J.C.M; Australasian Genomic Technologies Association (AGTA) PhD Top-Up Scholarship to M.D.

Availability of data and materials

The metformin aging data has been deposited in GEO with accession number GSE194386.

Details on additional results are provided in supplement.

Authors' contributions

J.C.M., A.T.F. and M.D. formulated the problem. M.D. developed the *scShapes* method and software with input from J.C.M. and A.T.F. M.D designed and implemented the simulations and applied *scShapes* to the case studies on real scRNA-seq data. J.C.M., A.T.F. and M.D. interpreted the results with input from A.S.K. J.C.M., A.T.F. and M.D. wrote the manuscript with input from A.S.K. All authors read and approved the final version of the manuscript.

Competing interests

The authors declare that they have no competing interests.

Ethics approval and consent to participate

Not applicable.

Figures

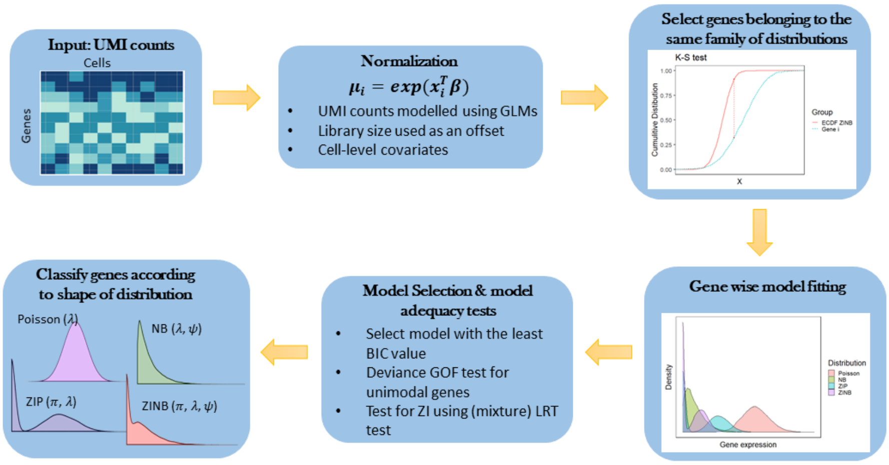

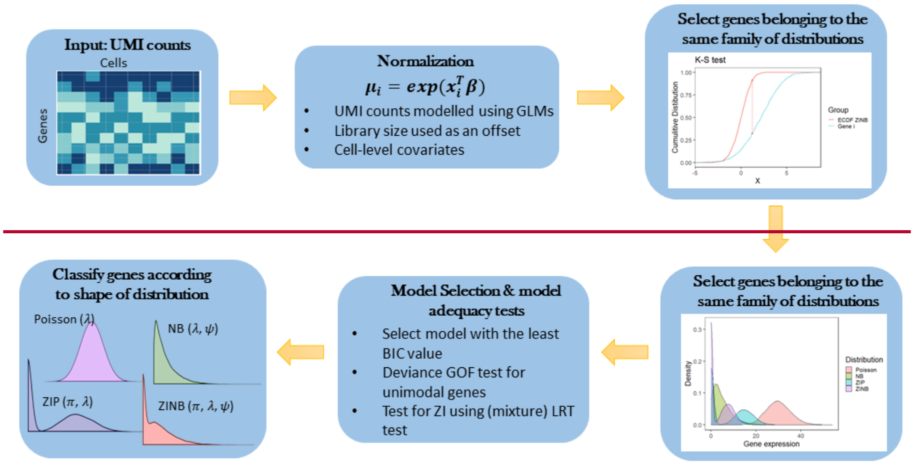

Figure 1: ScShapes pipeline for identifying gene expression according to distribution shape. The input to *scShapes* is a matrix of UMI counts with initial quality control performed to remove lowly-expressed genes. A Kolmogorov-Smirnov test is first performed to select genes belonging to the family of ZINB distributions. Genes that do not belong to this category are removed from further analysis. For genes that are assumed to follow a distribution from the ZINB family, the best distribution that explains most of the heterogeneity in its gene expression values is selected from Poisson, NB, ZIP, ZINB distributions based on two rounds of model adequacy testing.

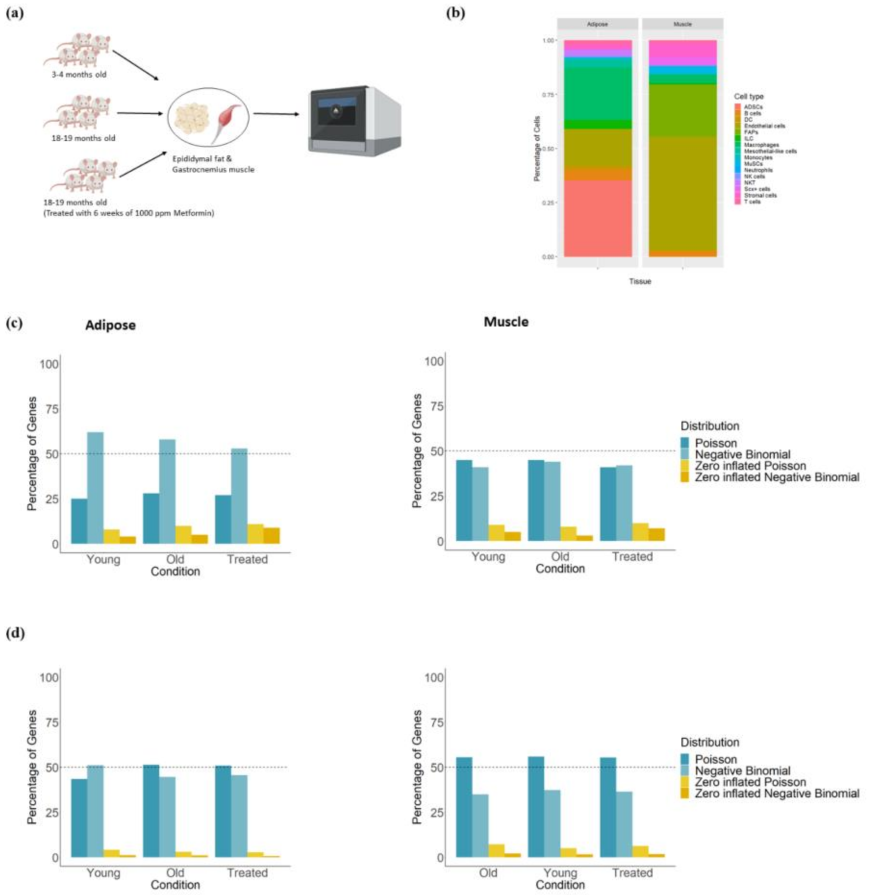

Figure 2: (a) Overview of the study design for the single-cell RNA-sequencing dataset using adipose and skeletal muscle in aging and metformin-treatment in mice. Single cells have been isolated from epididymal fat and gastrocnemius muscle of four male C57BL/6J mice in each of the three groups old (18-19 months), young (3-4 months) and metformin-treated for 6-weeks (18-19 months). Single cell library preparation was done using the 10X Chromium single cell 3' v2 kit, with quality control and gene expression quantification carried out in Cellranger. **(b)** Cell type abundances of the single-cell RNA-seq data in adipose and skeletal muscle. Seurat R package has been used for clustering and differential expression. Cells have been annotated manually by inspecting the gene expression profiles of marker genes identified using *FindAllMarkers* in Seurat for each cluster and validated using a self-built Garnett cell type

classifiers. The cell type membership was used as an explanatory covariate in the GLM framework of *scShapes*, to determine whether using prior biological knowledge would change the composition of genes following one of the four distributions. Bar plots for the percentage of genes following each distribution; (c) only accounting for technical variability, i.e. after adjusting for differences in sequencing depth and mouse ID; (d) accounting for both technical variability and known sources of biological variability, i.e. also adding cell type membership as a covariate in the GLM.

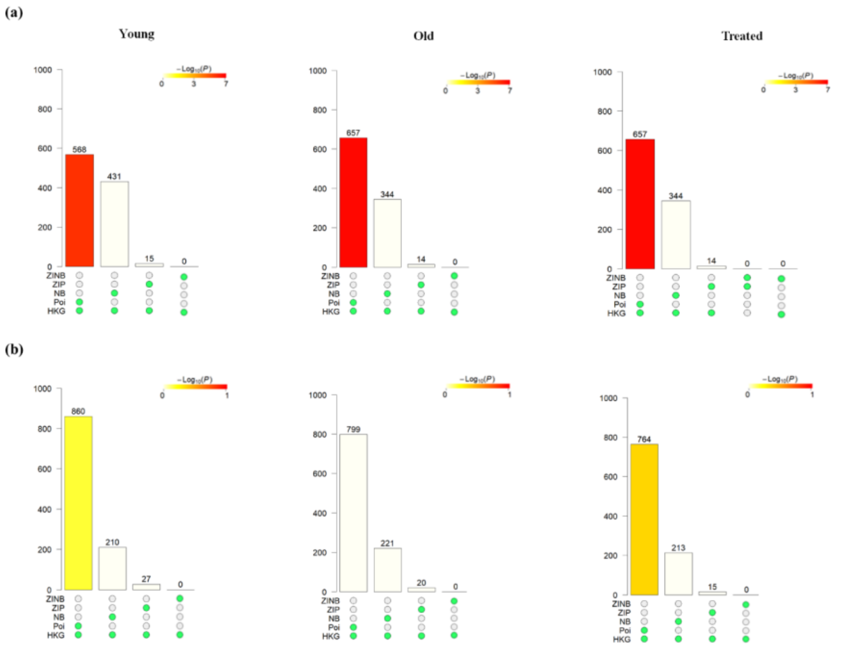

Figure 3: Investigating the prevalence of housekeeping genes (HKG) amongst genes that remain P, NB, ZIP, and ZINB both with models correcting for technical variability and when accounting for known biological variability in (a) adipose and (b) muscle. *scShapes* modelling framework is first applied to identify the distribution shape in old, young and metformin treated group only accounting for the technical variability in the GLM model (i.e. only including the offset term and mouse ID as a covariate in the GLM to account for differences in sequencing depth and biological replicates respectively). The same process is repeated again including information on the cell-types as a covariate in the GLM (in addition to the offset and mouse ID) to account for known sources of biological variability. We next checked for the overlap of genes that followed the same distribution in both instances above

and checked for the statistical significance of the overlaps using the R package *SuperExactTest*. The height of each bar corresponds to the number of overlapping genes and the colour corresponds to the $\log_{10}$(P-adjusted) value that assess the significance of the overlap.

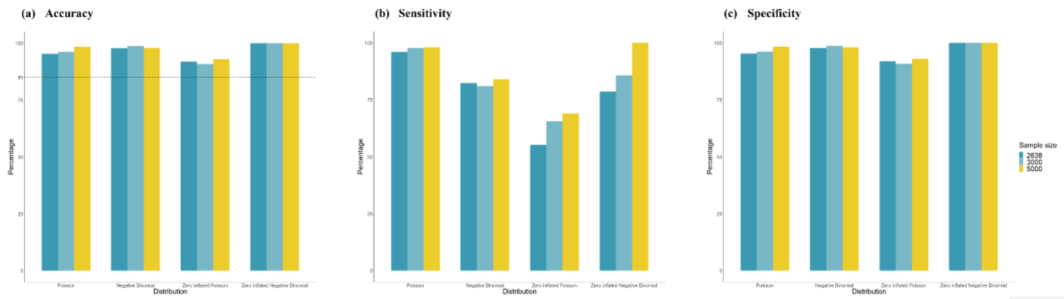

Figure 4: Summary statistics for the simulation study carried out to assess the performance of *scShapes* at identifying distributions of single cell gene expression data. Summary statistics (a) Accuracy, (b) Sensitivity and (c) Specificity; of the simulation study conducted to evaluate *scShapes*'s ability to classify genes into a P, NB, ZIP and ZINB distribution. Model parameters of the four distributions were estimated using the 3k PBMC dataset and were used for simulating gene expression values from P, NB, ZIP, ZINB distributions using three sample sizes *n* (i.e. number of single cells). Next, *scShapes* was run on the simulated gene expression counts to identify the distribution of the simulated data. Results of the simulation study was evaluated using the three summary statistics accuracy, sensitivity and specificity.

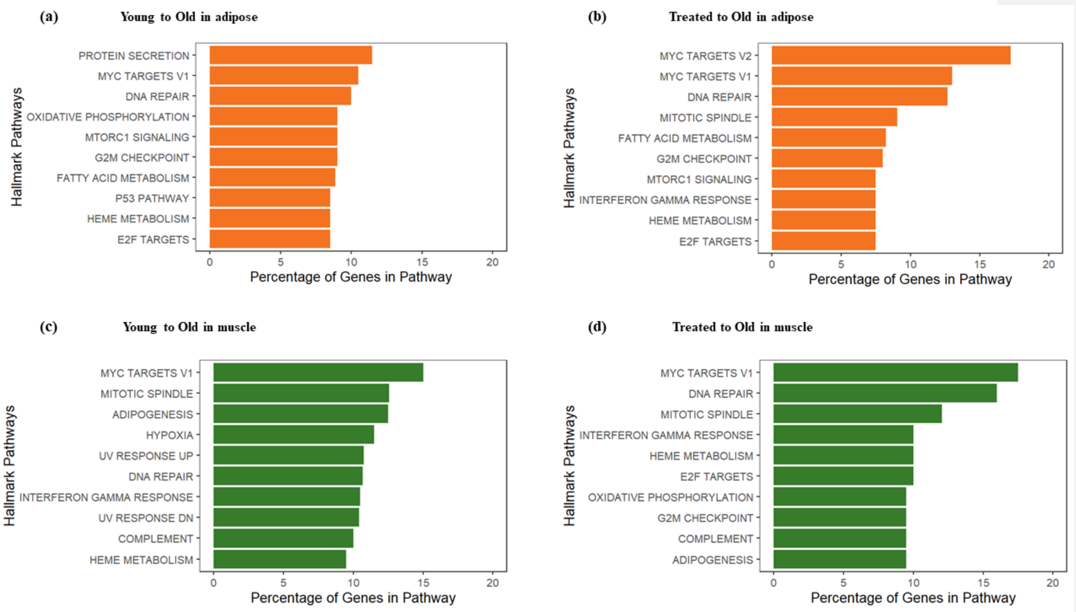

Figure 5: Pathway over-representation analysis of differentially-distributed genes detected by scShapes. The top 10 significant pathways (BH adjusted P-value $< 1 \times 10^{-4}$) in the pairwise comparisons (a) Young to Old in adipose (b) Treated to Old in adipose (c) Young to Old in muscle (d) Treated to Old in muscle using Hallmark pathways. The length of the bar corresponds to the percentage of differentially distributed genes that were represented in the Hallmark gene set.

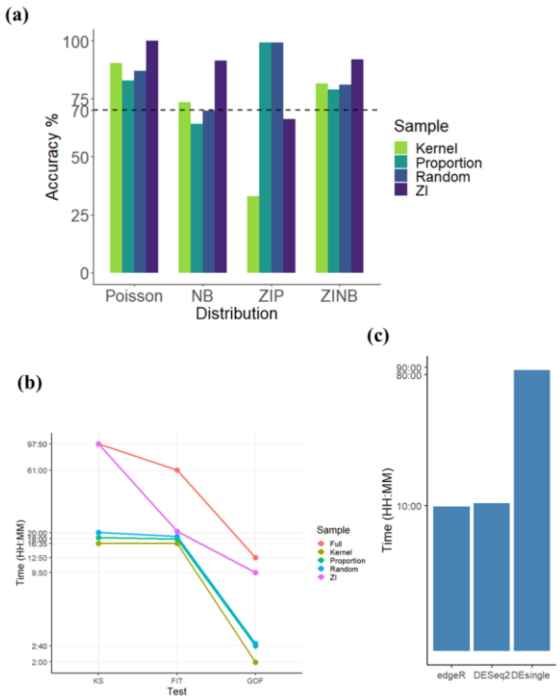

Figure 6: *scShapes* computational needs. The computational needs and accuracy of *scShapes* were benchmarked using PBMCs collected from eight volunteers at day 0, 2 and 7. The dataset comprised of ~162,000 cells. *scShapes* was run on each time point separately and the results of each time point were averaged together for the benchmarking purpose. This resulted in an average ~54,000 cells in the full dataset and ~8,000 cells in the down sampled dataset. The following four strategies were followed ; (1) randomly sample 1000 cells from each cell type (Random); (2) density based sampling of 1000 cells from each cell type (Kernel); (3) randomly sample cells totaling to 8000 cells whilst maintaining the original cell proportions (Proportion); (4) introducing an additional test for zero-inflation (ZI). *scShapes* was run each condition seperartely (dau 0, 2, 7) (a) Bar plots summarizing the average accuracy of the four methods in identifying the correct distribution. (b) Average Computational times required to run *scShapes* on the full dataset and by each of the four methods (Random, Kernel, Proportion and ZI). The modelling and testing framework of *scShapes* was summarized under three main steps; (1) KS test to identify the genes belonging to the family of ZINB distributions; (2) model fitting using the four distributions Poisson, NB, ZIP and ZINB (FIT); (c) goodness of fit tests to identify the best model for each gene (GOF). To benchmark the computational times of scShapes vs existing methods, we ran edgeR, DESeq2, DEsingle and scDD on the pairwise comparisons (on the full dataset), day 0 vs day 2; day 0 vs day 7 and computed the average time taken by each of the four methods. (c) Bar plot summarizing the average computational

Formatted: Font: Bold

Formatted: Font: Bold, Italic

Formatted: Font: Bold

Formatted: Font: Italic

Formatted: Font: Italic

Formatted: Font: Italic

Formatted: Font: Italic

times taken by exiting DE/DD methods (computation of scDD aborted due to limitations in memory).

Click here to access/download;Figure;Figure 1.png

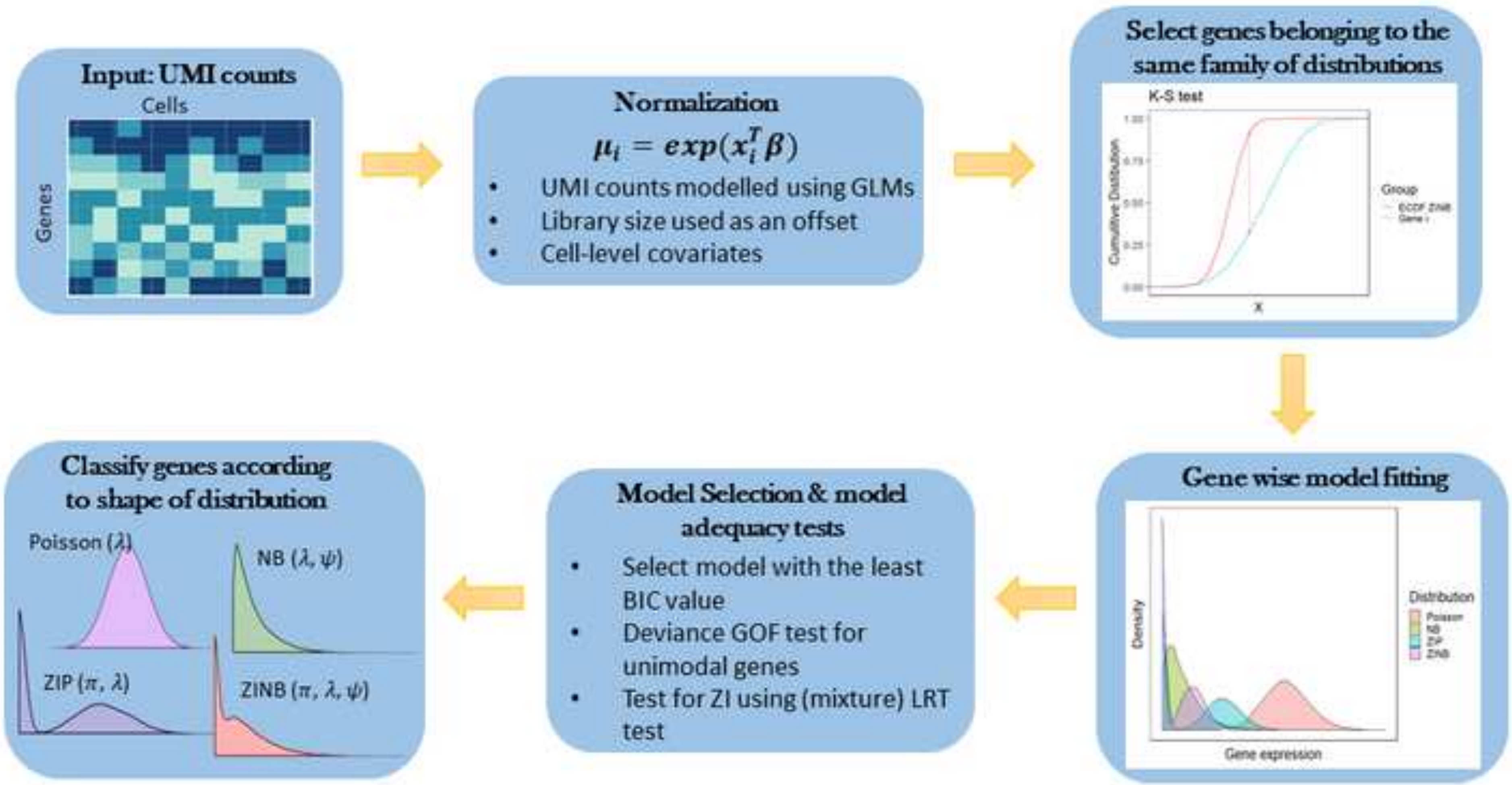

Click here to access/download;Figure;Figure 2.png

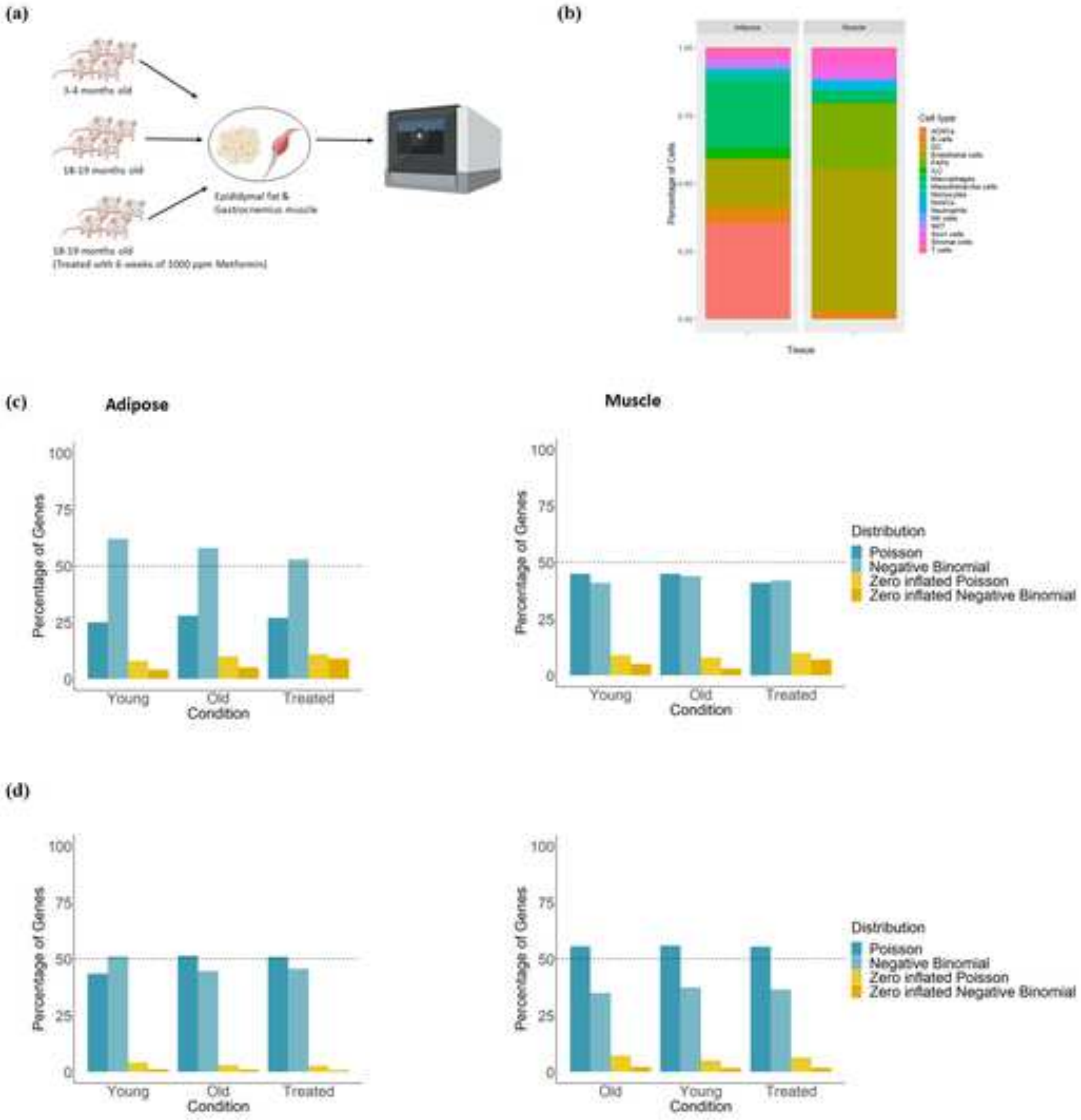

Click here to access/download;Figure;Figure 3.png

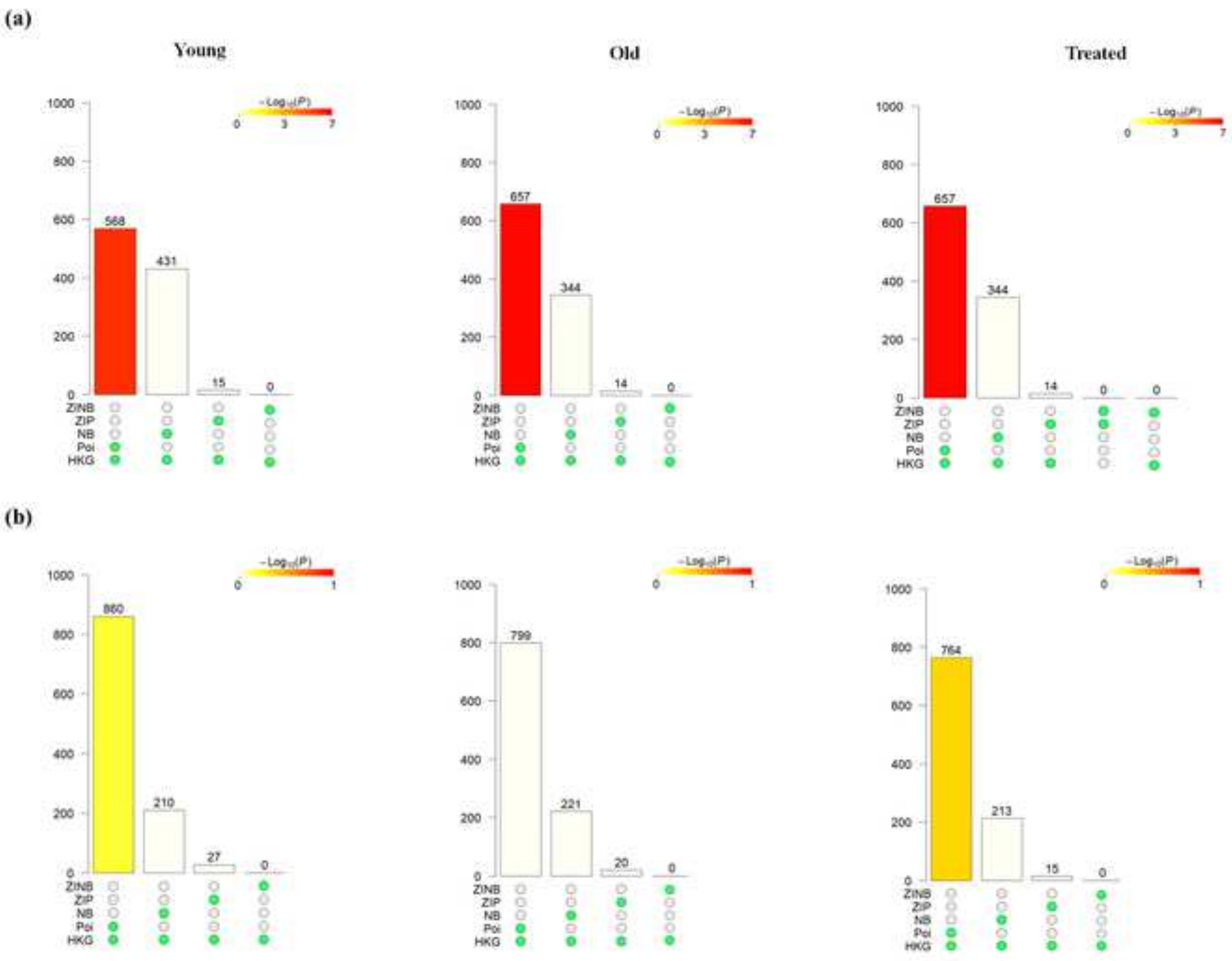

Click here to access/download;Figure;Figure 4.png

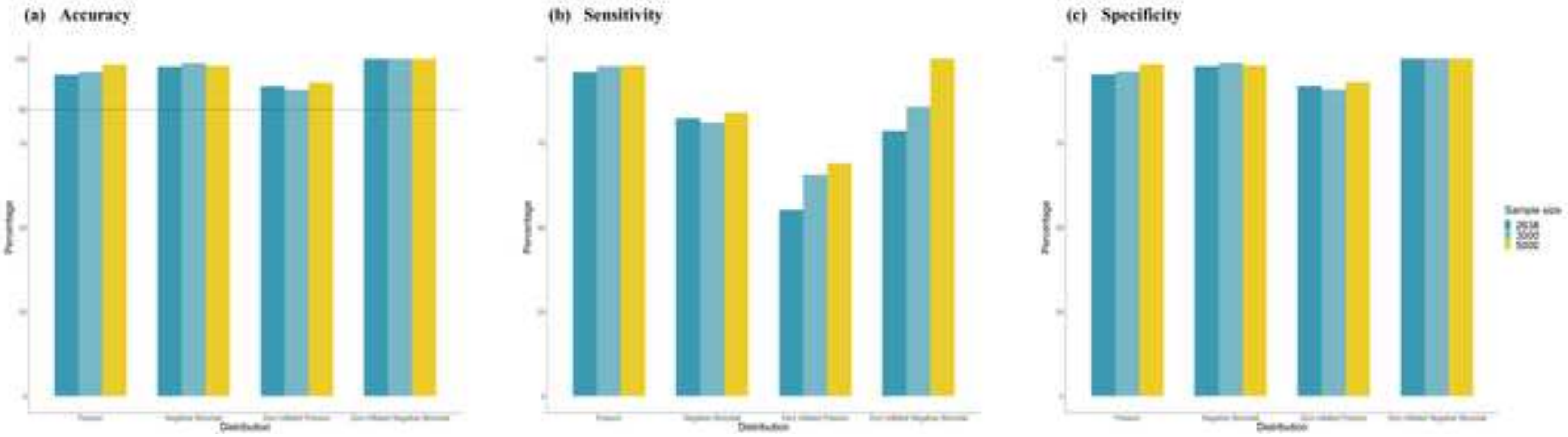

Click here to access/download;Figure;Figure 5.png

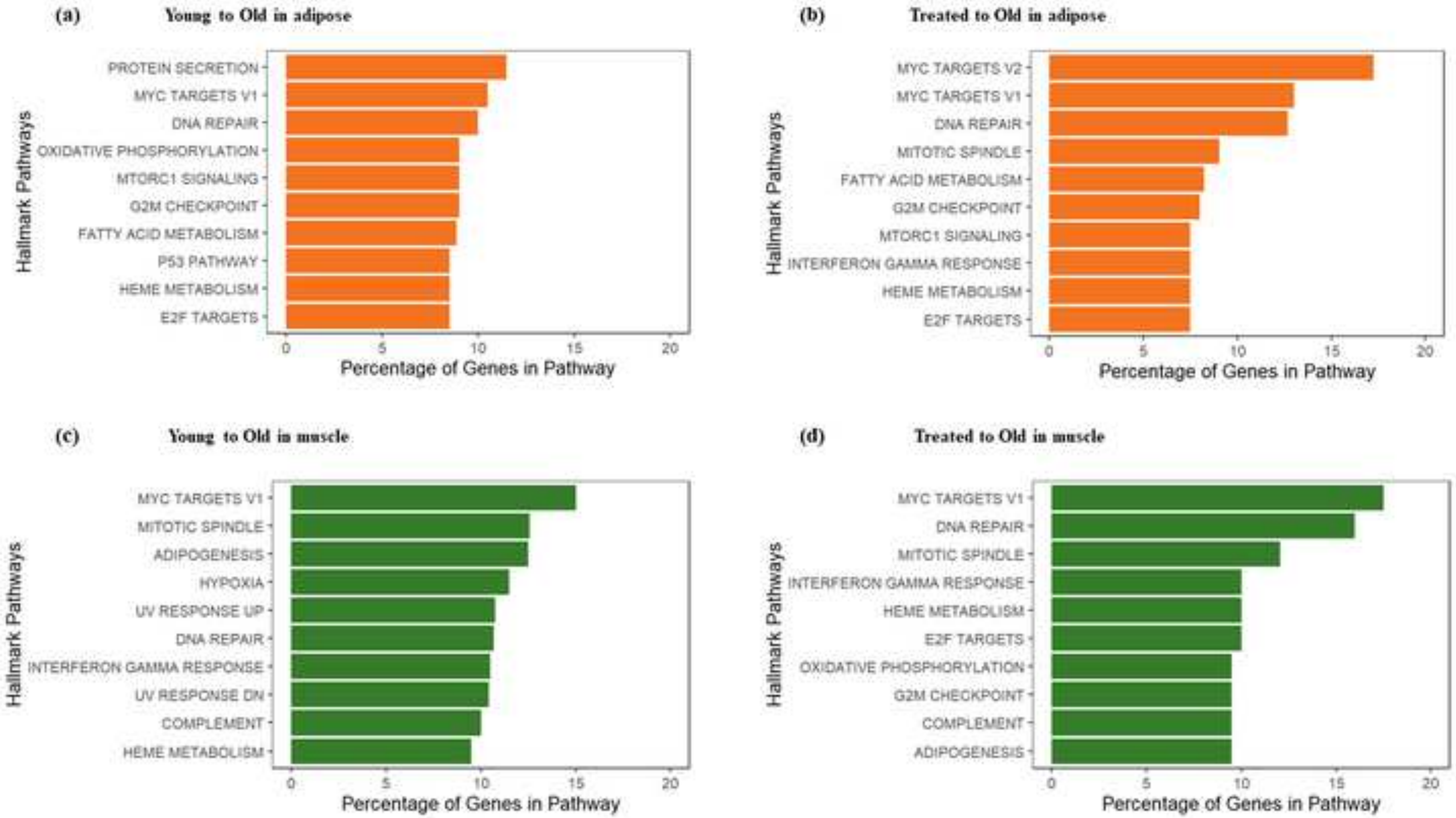

Click here to access/download;Figure;Figure 6.PNG

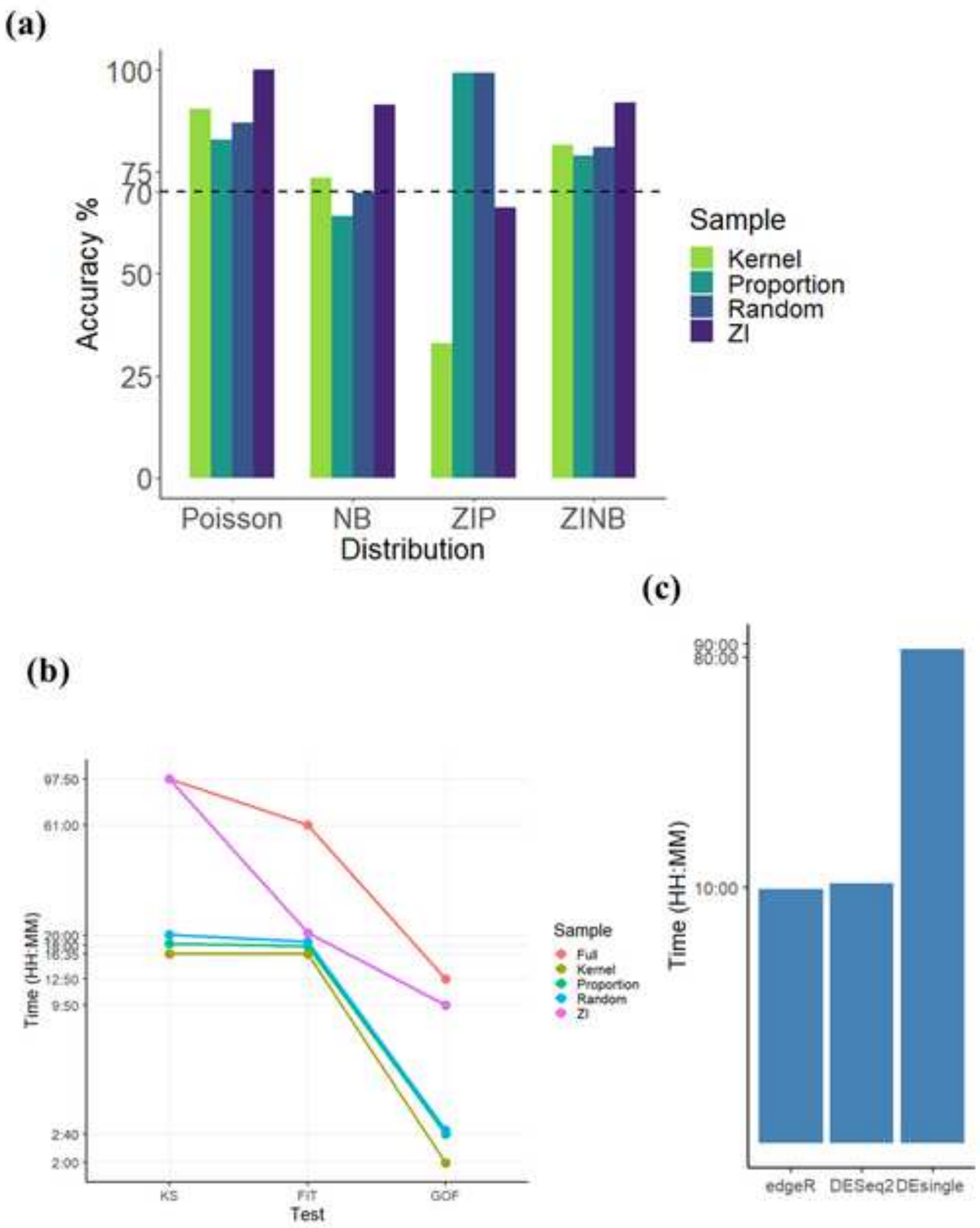

Click here to access/download
Supplementary Material
Supplementary Materials_GigaScience_120822.docx

Click here to access/download
Supplementary Material
Figure S1.png

Click here to access/download
Supplementary Material
Figure S2.png

Click here to access/download
Supplementary Material
Figure S3.png

Click here to access/download
Supplementary Material
Figure S4.png

Click here to access/download
Supplementary Material
Figure S5.png

Click here to access/download
Supplementary Material
Figure S6.png

Click here to access/download
Supplementary Material
Figure S7.png

Click here to access/download
Supplementary Material
Figure S8.png

Click here to access/download
Supplementary Material
Figure S9.png

Response to Reviewers Click here to access/download;Personal Cover;Reviewer reports_22Aug2022.docx

Response to Reviewers Comments

We thank all reviews for a set of highly constructive comments on our manuscript! Where possible, we have addressed the points raised by each reviewer and the manuscript has improved based on their feedback. For suggestions that necessitated changes that we felt were out of scope, we hope that the clarifications that have been provided are satisfactory and insightful. Line and Page numbers provided correspond to the document with tracked changes displayed.

Response to Reviewer 1

How to model the statistical distribution of the gene expression, is a basic question for the field of single cell sequencing data mining. Dharmaratne and colleagues looked details at the distribution of very gene. By using the generalized linear models (GLM), the authors present a new program scShapes, which matched a specific gene with a distribution from one of the four shapes, Poisson, Negative Binomial (NB), Zero-inflated Poisson (ZIP), and Zero-inflated Negative Binomial (ZINB). As the authors present in this manuscript, not all genes adapted to a single distribution, neither NB or Poisson, and some of the genes actually adapted to the zero-inflated models because of the property of high drop-out rate in the modern single cell sequencing, says 3' tag sequenced. It is has been popular to employ GLM in single cell data mining recently, but it also got both praise and blame. So it is a good forward step to model a specific model for an individual gene. But the bad side is the computing cost, especially for the number of cells been sequenced reach to millions in currently research, and it believed that the dataset will be reached even bigger in the future. So it make a great obstacle arise to the application of the method presented by the author here. How to speed up the calculation using the mixed model or scShapes?

We agree with this reviewer's assessment regarding the trade-off that exists between positive benefits from increased specificity by modelling individual genes versus the increase in computational cost associated with this. We have added a section to the manuscript (see *Benchmark of scShapes computational needs*) that investigates this topic more comprehensively. In response to this reviewer's question about how to speed up the calculations, we have explored two different ways of doing this, including down-sampling and pre-filtering for zero inflation, and characterized the extent of computational time saved and the consequences on accuracy that are incurred. Please also see our response to Point 4 (next page) for a more detailed discussion.

The authors also performed the scShapes on some datasets, including the metformin, human T cells, and PBMCs. They found some potential genes that changed the distribution shape, but didn't easy to be identified by other methods. It demonstrated that scShapes could identified the subtle change in gene expression.

Major points:

(1) We didn't see any details about the metformin dataset, the segueing depth and quality, number of genes/UMIs per cell, and so on. It makes hard to evaluate the quality and reliability of the results generated by scShapes. If this dataset is another manuscript could not possible to be presented at the same time, I suggest the author could perform on alternative dataset, as there are so many single cell datasets has been published could be used in this study.

We agree with the reviewer and have updated the manuscript to now include the quality metrics associated with the Metformin dataset (see Supplementary Material). We have included detailed descriptions and visualizations related to the quality control metrics of the Metformin dataset.

The following paragraph has been added:

Single cell RNA-sequencing of Metformin dataset

Single cells have been isolated from epididymal fat and gastrocnemius muscle of four male C57BL/6J mice in each of the three groups old (18-19 months), young (3-4 months) and metformin-treated for 6-weeks (18-19 months). Single cell library preparation was done using the 10X Chromium single cell 3' v2 kit, using ~3000 cells per lane. The libraries have been sequenced at a target depth of 50 million reads/sample on Illumina NovaSeq S4. Quality control on the raw sequence reads have been performed using OpenGene's fastp v0.19.4 preprocessor [10] followed by genome alignment to mm10 mouse reference genome using 10X cellranger 3.0.2. software. Gene expression have been quantified using unique molecular identifiers (UMIs)

using the default cellranger 3.0.2. pipeline. Barcodes with a total UMI count >500 have been classified as cells. Further QC, pre-processing, and analyses have been done using Seurat 3.1.1.

The QC metrics have been visually examined (Figure S8-S9) and cells passing the following QC thresholds proceeded for normalization and analyses: cells with <500 unique genes detected, cells with a total RNA count > 20000 (inferred as doublets) and > 10% mitochondrial genes within the whole transcriptome (inferred as cells undergoing apoptosis).

We agree with the reviewer that many datasets are available for scRNA-seq data analysis. However, not all datasets may be applicable or optimal for *scShapes* as the main intended application of *scShapes* is to investigate more subtle changes in phenotype, of which ageing is a great example. To take some of the emphasis and reliance away from the Metformin dataset, other datasets were also used, and we refer the reviewer back to these as a demonstration of *scShapes'* wider applicability.

(2) Even the authors taken the cell type account in the GLM, I wonder for a specific gene, whether the distribution shape will change in different cell type. If so, it will becoming more complex, that is need to model the distribution shape for individual gene for every cell type alone.

Yes, the reviewer's comment is correct. Some genes will change their distribution shape for different cell types. Although this comparison of looking at global versus cell type-specific changes was not investigated explicitly, we did investigate cell type-specific changes as detected by *scShapes* for the COVID-19 dataset (see line 425, p19). We used *scShapes* to identify genes that had changes in differential distribution between the infected and healthy control group in PBMCs. We also then investigated what changes could be detected at the cell type-specific level for seven main cell types.

The influence of cell type membership on differentially-distributed genes does represent an additional level of complexity to an analysis of scRNA-seq data. While this can be performed using *scShapes*, as demonstrated by our COVID-19 case study, it is also important to recognize that modelling distributions for at the level of a specific cell type invokes several statistical considerations, mainly around sample size. We may not have enough cells for a particular cell type to model a distribution adequately or the number of cells may also be quite variable from one cell type to another. There are ways that we could address these issues, e.g. using pseudo bulking. As such, investigating how to model the distribution shape of individual genes for every cell type distribution seems like a next level application of the *scShapes* framework.

(3) To identify the different gene expression in scShapes, the author didn't consider the influence of different cell number, or the proportion of cell number, in the different cell type. A possible way to evaluate or eliminate this bias is to down sampling from a big dataset, instead of just simulated total number 2k ~ 5k from the PBMC. To evaluate the influence both the total number cell and the proportion in cell type.

We thank the reviewer for this helpful suggestion and have now included such a comparison under the section "*Benchmark of scShapes computational needs*" (see line 459, p20). For this comparison, we used a PBMC dataset with 161,764 cells collected at three time points from eight volunteers. *scShapes* was applied on the three time points (~54,000 cells each) and compared to a series of down sampling runs from the total cells. For down sampling we followed three approaches; (1) randomly sampling 1000 cells from each cell type; (2) density-based sampling of 1000 cells from each cell type; (3) randomly sampling cells totalling to 8000 cells whilst maintaining the original cell proportions. We have then compared the results from the three sampling strategies in terms of their accuracy as well as computational times. We hope that this additional comparison helps address the reviewer's concern.

(4) The author should present the comparative results of the computational cost for different methods. Says the accuracy, time and memory consuming under different number of cells. I suggest the authors use much a larger dataset, because currently single cell research may include millions of cells, and the ability to process big data is very important to the application and becoming a widely used one.

We have now included this comparison under the same section as above "Benchmark of *scShapes* computational needs" (see line 459, p20). Here we benchmarked the computational time and accuracy of *scShapes* under different cell numbers, cell proportions as well as four other popular tools for identifying DE/DD genes, namely *edgeR*, *DESeq2*, *DEsingle* and *scDD*. Upon the advice of the reviewer, we have used a much larger dataset on PBMCs with 161,764 cells.

Minor points:

(1) No figure legends for Fig.2 c and d.

The figure legends have been corrected (see line 1038, page 44).

(2) It is unclear whether the total 30% genes undergo shape change, or just the proportion of the remaining after the pipeline. So please clarify the details.

We have revised the text to add more clarification to address this point (see line 236, page 10);

Nearly, 30% of the genes *(after filtering, i.e. of the genes with at least 10% expression across all cells within a treatment condition)* were differentially distributed in at least one of the comparisons (Old vs Young or Old vs Treated) in both tissues adipose and muscle.

Response to Reviewer 2

In this manuscript, authors presented a novel statistical framework scShapes using GLM approach for identifying differential distributions in genes across scRNA-seq data of different conditions. scShapes quantifies gene-specific cell-to-cell variability by testing for differences in the expression distribution. scShapes was shown to be able to identify biologically-relevant switch in gene distribution shapes between different conditions. However, there are still several concerns required to be addressed.

1. In this study, authors compared scShapes to scDD and edgeR. However, besides these two, there are many other methods for calling DEGs from scRNA-seq. Wang et al. (2019) systematically evaluated the performance of eight methods specifically designed for scRNA-seq data (SCDE, MAST, scDD, D3E, Monocle2, SINCERA, DEsingle, and SigEMD) and two methods for bulk RNA-seq (edgeR and DESeq2). Thus, it is also worthy to compare scShapes to other methods, such as SigEMD, DEsingle and DESeq2, which were supposed to perform better than scDD or edgeR.

We applied *edgeR's* quasi-likelihood approach (QLF) with the cellular detection rate as a covariate, since this method was shown to perform well for DE analysis of scRNAseq in Soneson et al. (2018). However, we do agree with the reviewer that in other reviews other tools like *DEsingle*, *DESeq2* have been shown to outperform *edgeR* or *scDD*. Hence, we have now included comparisons with both *DEsingle* and *DESeq2* (see line 299, p13).

2. When scShapes was compared to scDD, authors mainly focused on the distribution shifting. However, to users, it would be better to present a venn diagram showing the numbers of the genes detected by both scShapes and scDD, and the genes specifically identified by scShapes and scDD, respectively. In addition, authors showed the functional enrichment results for DEGs identified by scShapes. It is also worthy to perform enrichment analysis for the genes detected by both scShapes and scDD or specifically identified by scShapes or scDD.

This is a good suggestion and we have now included this comparison and the corresponding figures as suggested by the reviewer under **Supplementary Figure S4**. The following are discussed under the results section of the paper (see line 375, p16).

Nevertheless, we further investigated the genes identified by both scShapes and scDD to identify functional terms enriched in the overlapping gene sets. We observed that Hallmark pathways such as DNA repair and MTORC1 signalling were enriched in adipose between both pairwise comparisons. Similarly in muscle we observed that the Hallmark pathways such as adipogenesis and hypoxia are over-represented in genes identified to be differentially distributed by both scShapes and scDD between old and young (Figure S4).

3. Since scShapes detects differential gene distribution between different conditions, it would be better to show users how to interpret the significant results biologically. For example, authors mentioned that RXRA is differentially distributed between Old and Young and Old and Treated, so what does this results mean? Can this differential distribution be associated with differential expression?

Whilst there is overlap between differentially expressed genes and genes identified by *scShapes*, differential distribution cannot always be associated with differential expression. To showcase this, we discussed the example of Foxo3 which was uniquely identified to be differentially distributed by *scShapes*, but was not differentially expressed. Furthermore, *FOXO3* is unknown to be involved in regulating aging-associated stress response and is proposed to be mediated by metformin. Hence, we added the following text to help a

reader interpret these results (see line 259, p11). This is an example of how *scShapes* is capable of modelling and identifying subtle changes in phenotype, such as ageing.

We then further investigated some of the differentially distributed genes identified by scShapes. For example, we observed that the gene Foxo3, identified to be differentially distributed in both pairwise comparisons following a NB distribution and then switch distribution to a Poisson in both young/treated. This result indicates that during ageing we observe higher over-dispersion in the expression of Foxo3, than that could be modelled with a Poisson distribution. Hence it could be hypothesized that there are increased levels gene expression heterogeneity observed during aging compared to young phenotype, where treatment with metformin reverts the expression of Foxo3 with lesser over-dispersion, to levels comparable with the young phenotype. This observation could have been missed if we only focused on DE genes, as Foxo3 was not observed to be differentially expressed in either comparison.

4. In Discussion, authors mentioned that scRATE is another tool that can model droplet-based scRNA-seq data. It would be clearer to discuss that why authors develop their own algorithm rather than using scRATE to model the distribution.

We have now included the following text to address the reason as to why we implemented our own modelling approach (see line 571, p25).

Whilst scRATE also employs a GLM based modelling framework with Bayesian model selection, we developed our own modelling framework scShapes which utilizes maximum likelihood estimation (MLE) as Bayesian estimation is best suited for data with prior knowledge about the distribution it originated from.

5. In Introduction, authors talked about the zero counts in scRNA-seq data, and presented evidence in Results part. Since 2020, there are several publications also focusing on this issue, such as Svensson, 2020 and Cao 2021. These discussions should be included in this manuscript.

We have now included the following text under the discussion section of the paper to address these two papers that the reviewer has mentioned (see line 519, p23).

Recently, there has been discussion in the literature regarding the use of zero-inflated distributions for modelling single cell transcriptomic data. It is argued in Cao et al. [49] that zero-inflation is supressed in gene expression counts measured in terms of UMI counts, and in Svensson [50] that a NB distribution alone is sufficient to model the zero counts in droplet-based scRNA-sequencing data. However, this study [50] models the gene expression heterogeneity of negative control (e.g. ERCC spike-ins) droplet-based scRNA-seq data. As negative control data lack biological variation a NB distribution may sufficiently model such gene expression measurements and hence there remains motivation to model the excess zeros of at least a subset of the genes.
