# Supplementary figures and images for: scShapes: a statistical framework for identifying distribution shapes in single-cell RNA-sequencing data
Source: Gigascience. 2023 Jan 24;12:giac126. doi: 10.1093/gigascience/giac126 (PMC9871437; doi:10.1093/gigascience/giac126)

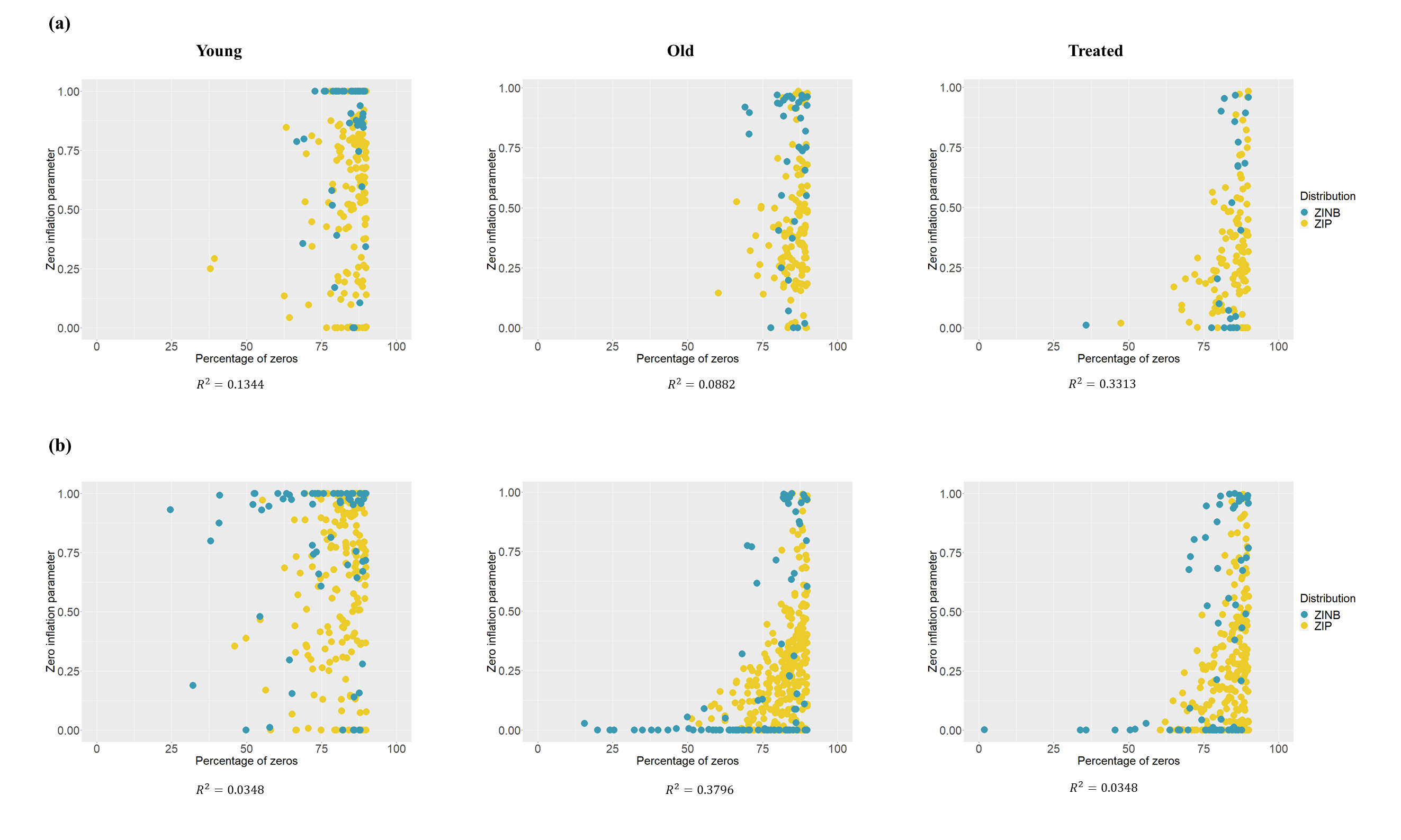

Supplement: giac126_Supplemental_Files [file giac126_supplemental_files.zip › Figure S1.png]

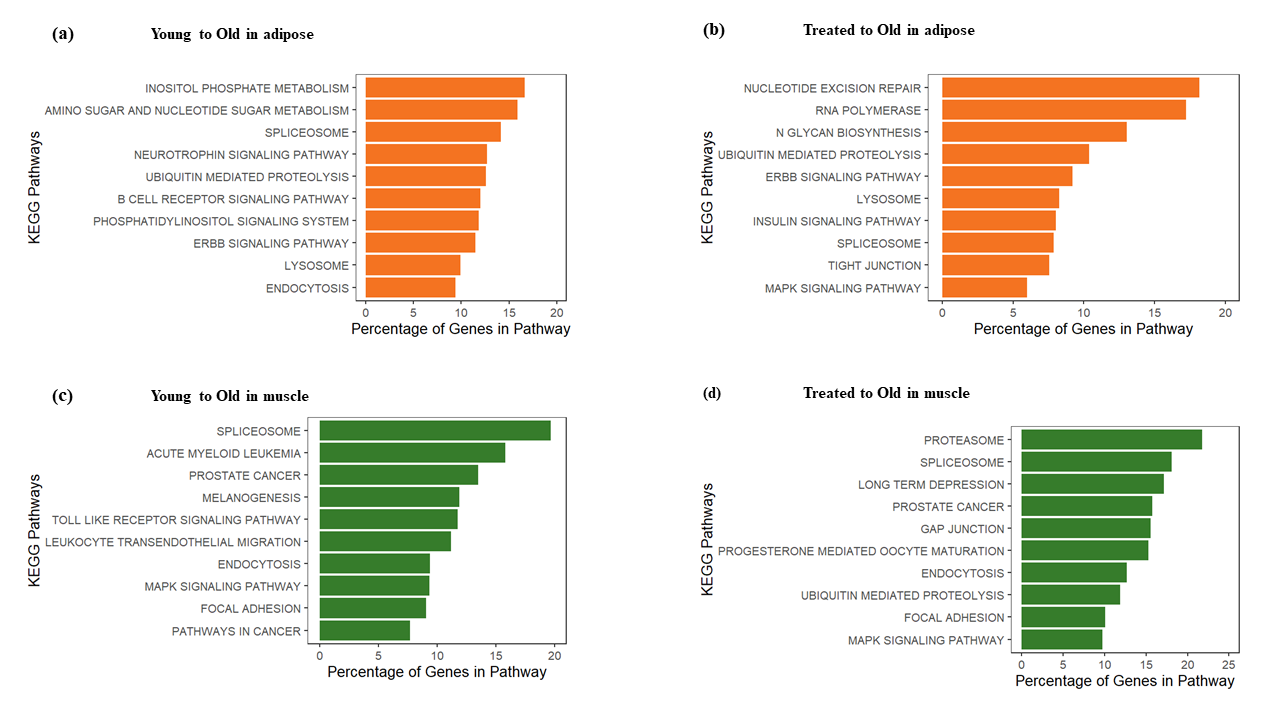

Supplement: giac126_Supplemental_Files [file giac126_supplemental_files.zip › Figure S2.png]

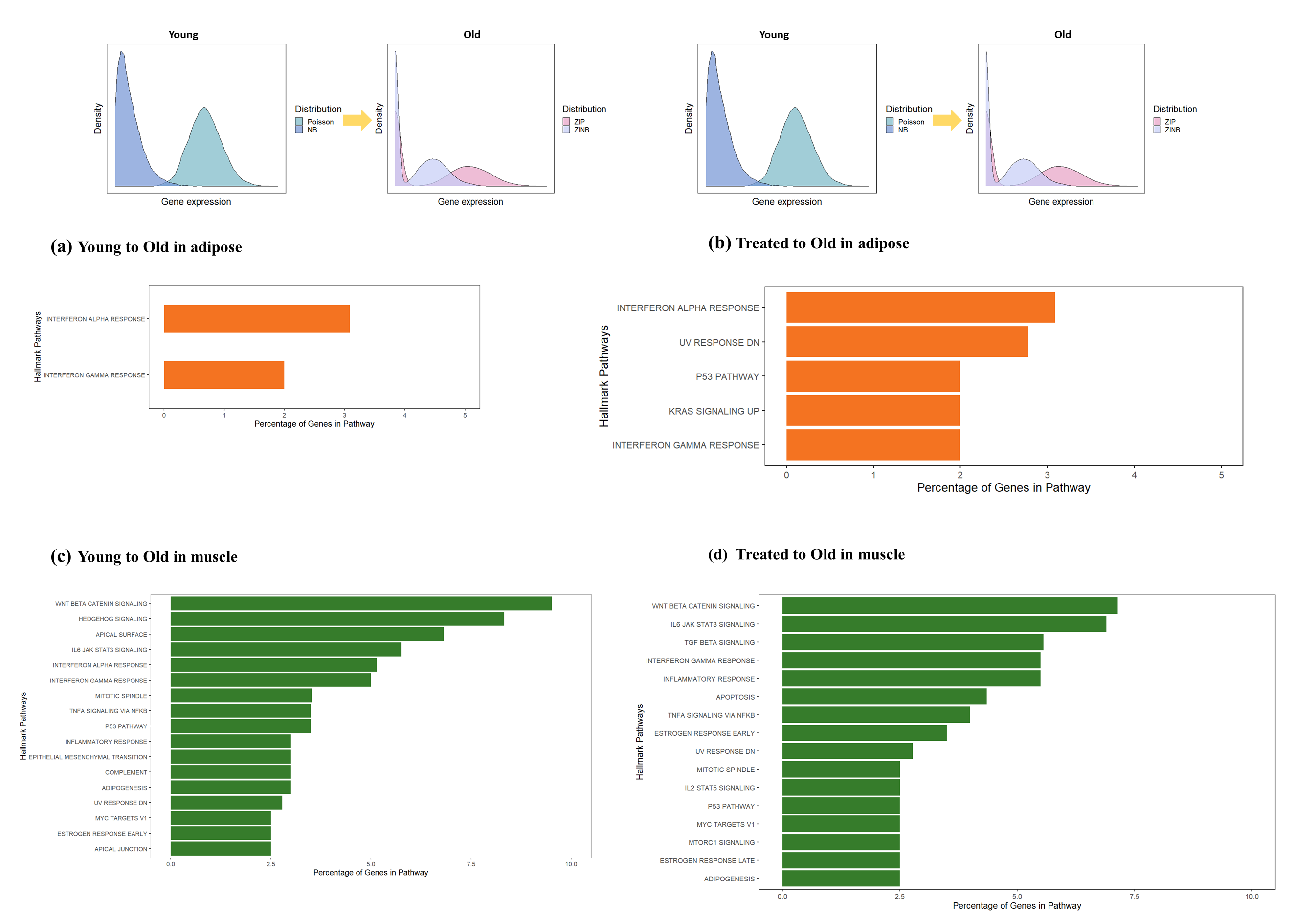

Supplement: giac126_Supplemental_Files [file giac126_supplemental_files.zip › Figure S3.png]

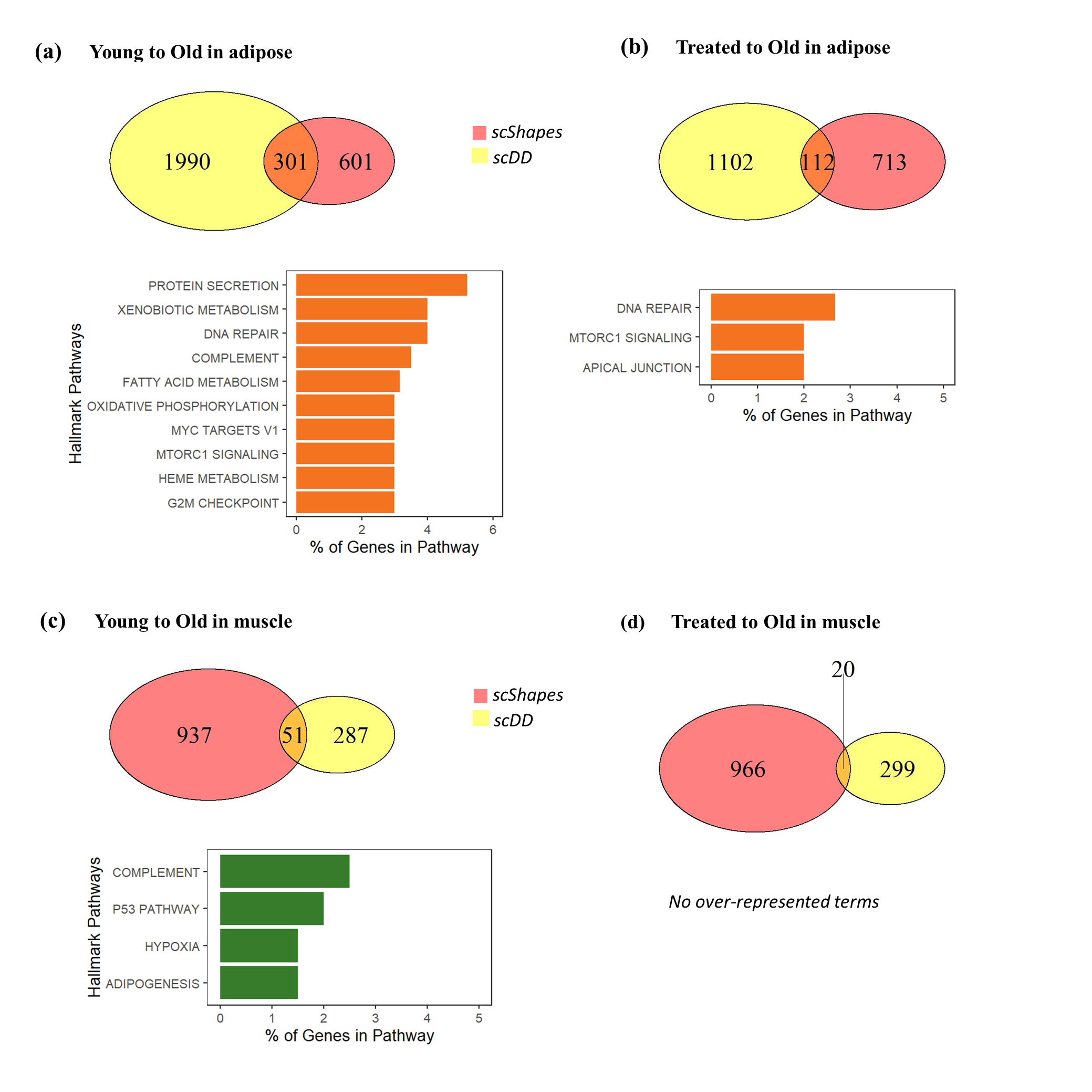

Supplement: giac126_Supplemental_Files [file giac126_supplemental_files.zip › Figure S4.png]

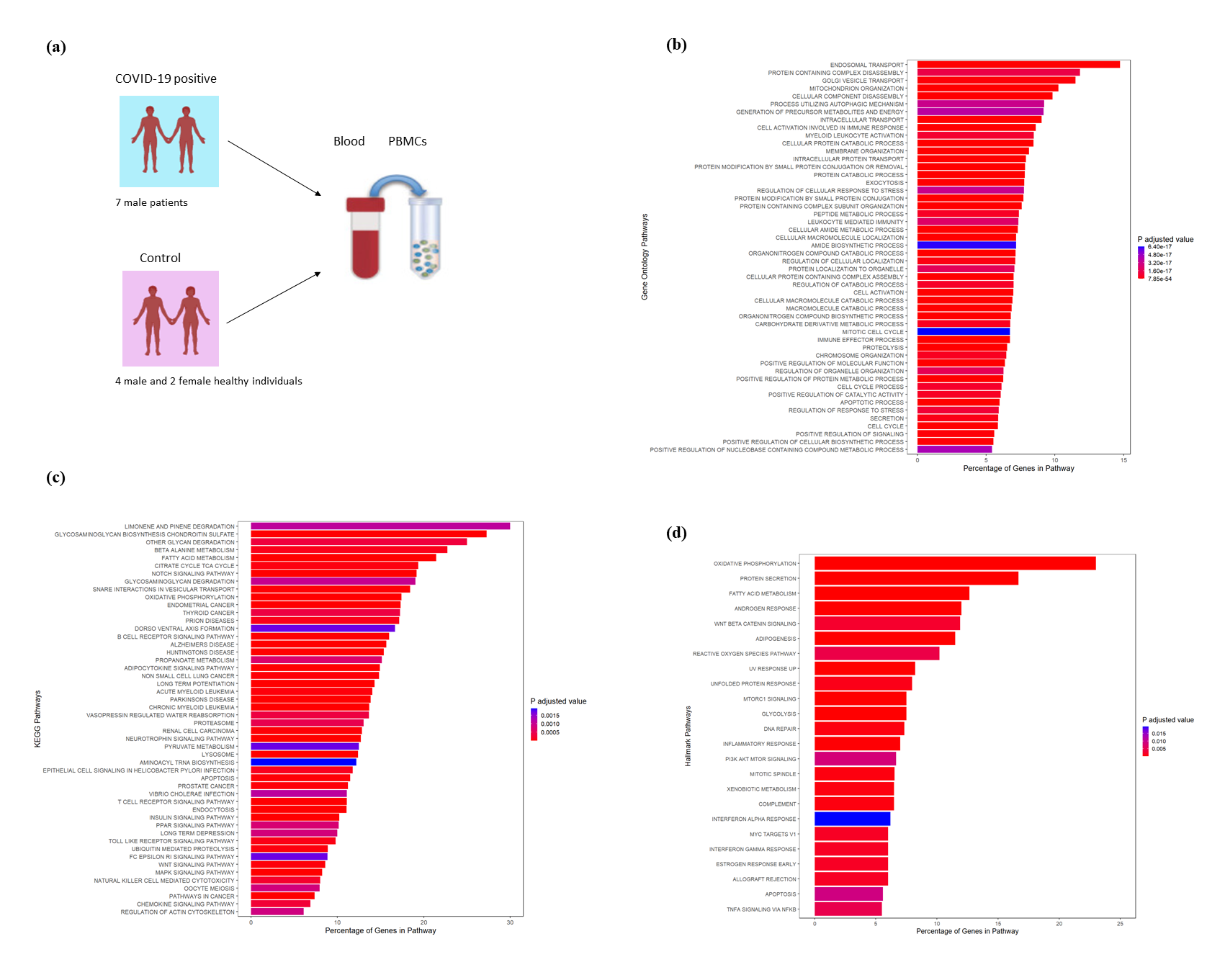

Supplement: giac126_Supplemental_Files [file giac126_supplemental_files.zip › Figure S5.png]

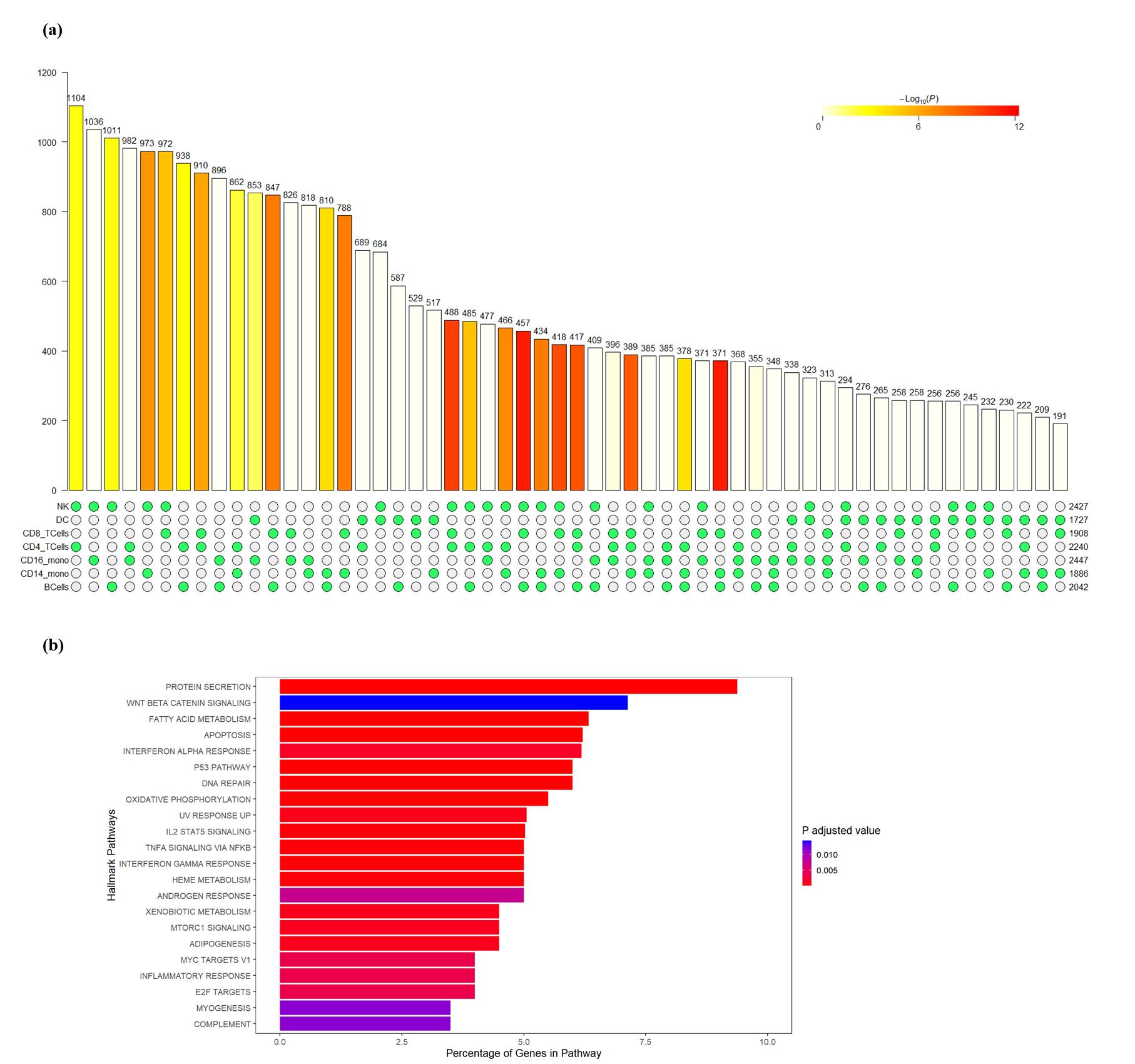

Supplement: giac126_Supplemental_Files [file giac126_supplemental_files.zip › Figure S6.png]

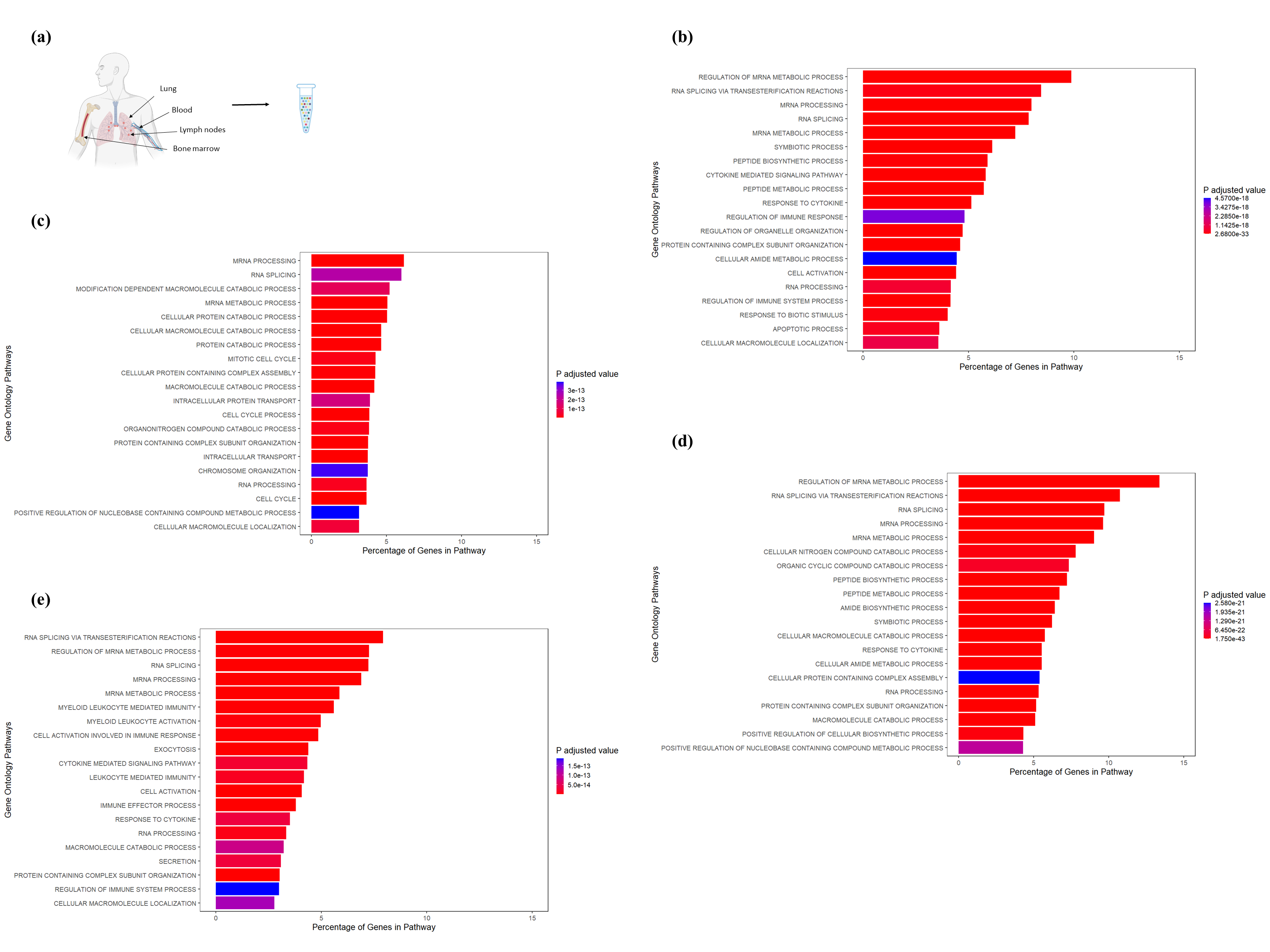

Supplement: giac126_Supplemental_Files [file giac126_supplemental_files.zip › Figure S7.png]

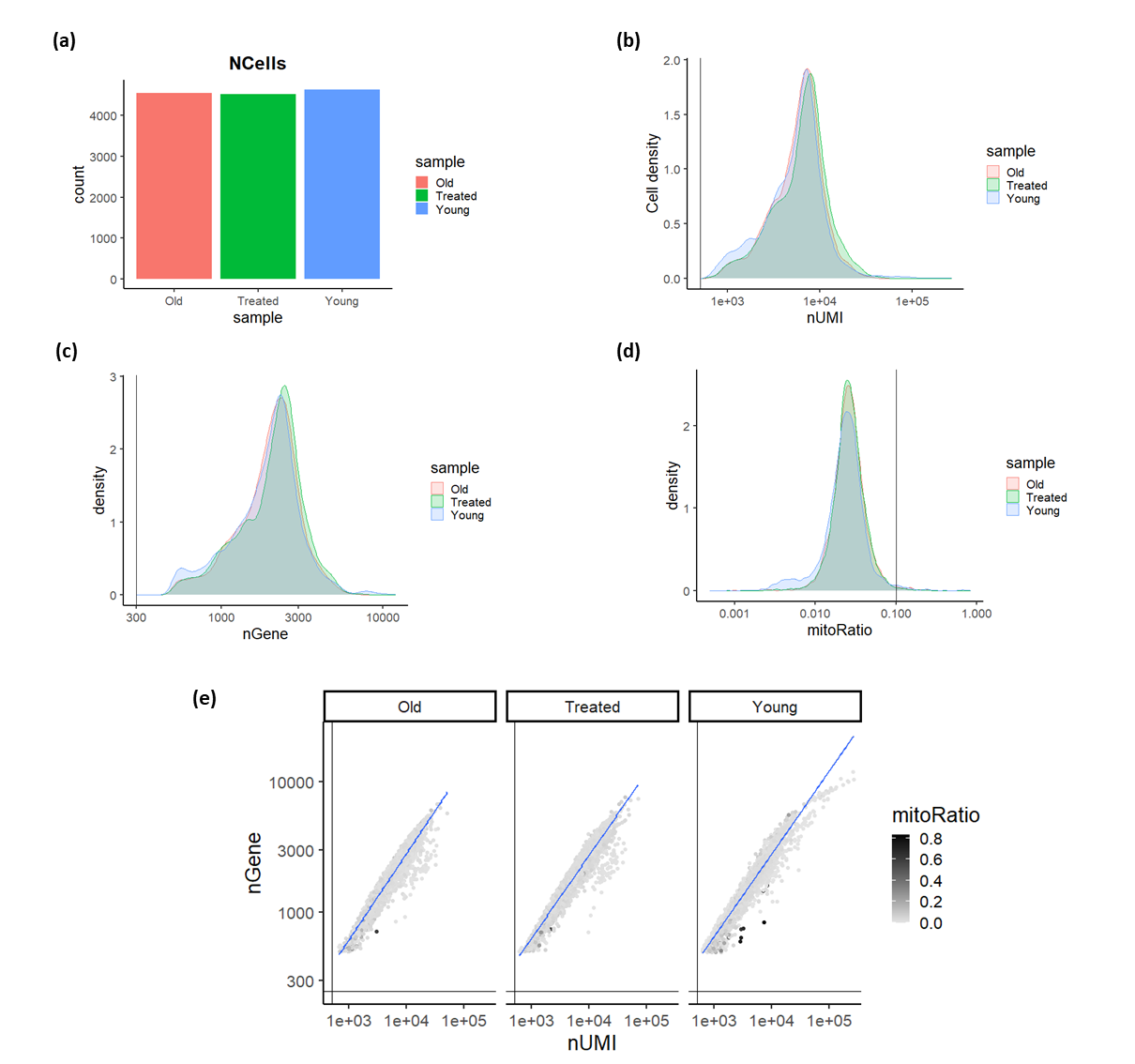

Supplement: giac126_Supplemental_Files [file giac126_supplemental_files.zip › Figure S8.png]

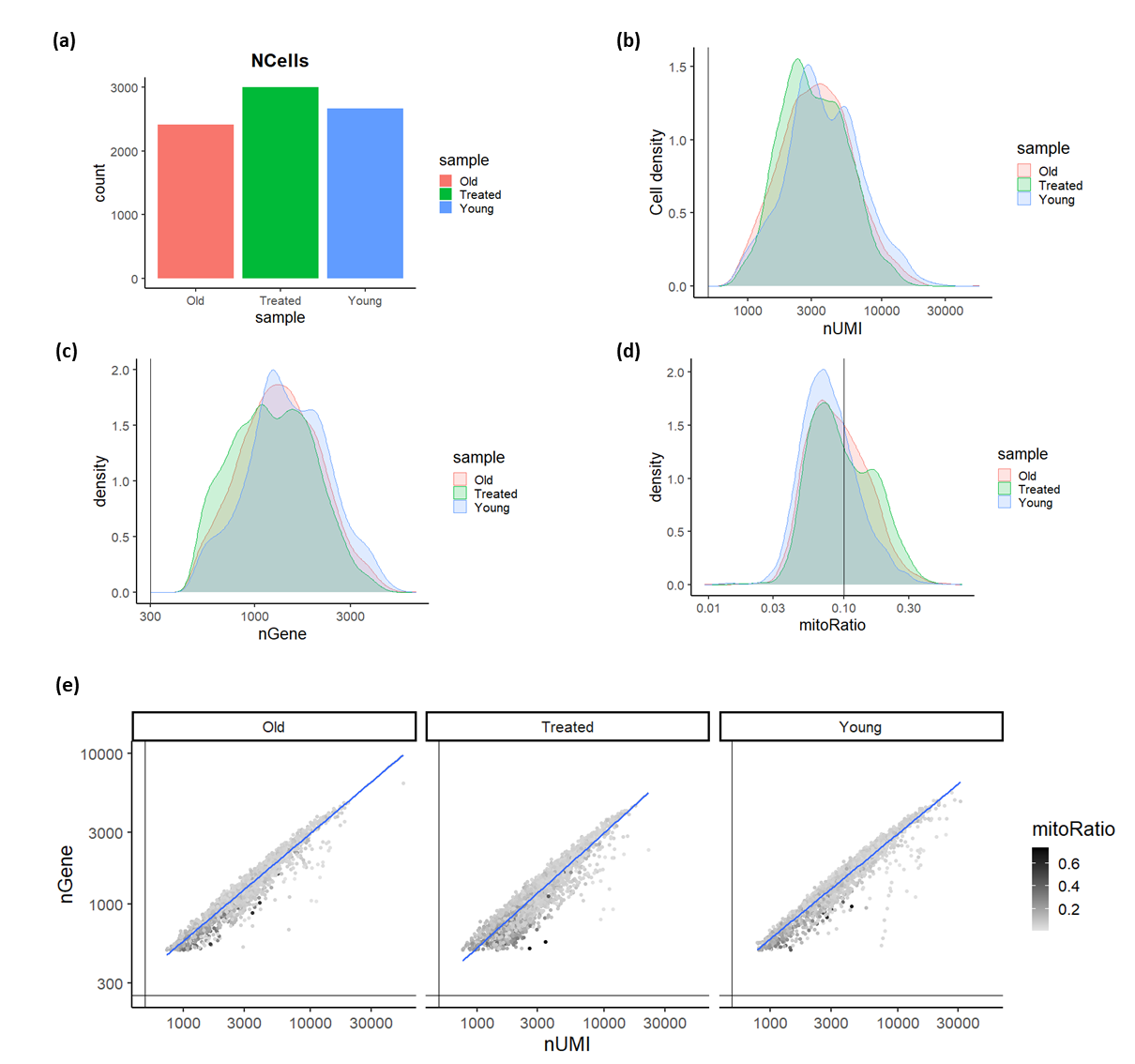

Supplement: giac126_Supplemental_Files [file giac126_supplemental_files.zip › Figure S9.png]
