# Supplementary material for: scShapes: a statistical framework for identifying distribution shapes in single-cell RNA-sequencing data
Source: Gigascience. 2023 Jan 24;12:giac126. doi: 10.1093/gigascience/giac126 (PMC9871437; doi:10.1093/gigascience/giac126)
Supplement: giac126_Response_to_Reviewer_Comments_Original_Submission [file giac126_response_to_reviewer_comments_original_submission.pdf]

Author's Response To Reviewer Comments

Close

Please see uploaded file of Responses to Reviewers Comments

Close
