# Supplementary material for: scShapes: a statistical framework for identifying distribution shapes in single-cell RNA-sequencing data
Source: Gigascience. 2023 Jan 24;12:giac126. doi: 10.1093/gigascience/giac126 (PMC9871437; doi:10.1093/gigascience/giac126)
Supplement: giac126_Response_to_Reviewer_Comments_Revision_1 [file giac126_response_to_reviewer_comments_revision_1.pdf]

Author's Response To Reviewer Comments

Close

We thank the reviewer for suggesting that scShapes be adapted to the GPU version and agree with the reviewer that this will help speed up the computational time. However, we believe that this is better suited as a future development of scShapes. Currently scShapes supports CPU acceleration via BiocParallel which can be configured for different parallel evaluation environments. The adaption of scShapes on to a GPU version will require changing the original R implementation of GLM to that, which supports GPU enabled functions such as gputools. Furthermore, certain implementations of GPU computing can be specific to one brand (e.g. NVIDIA) which may be a limitation for the end user. Therefore, we believe this would be a great future direction for scShapes and hence we have decided to add the following sentence to the Discussion of the manuscript;

"Additionally, graphical processing unit (GPU) acceleration of the GLM algorithms provides a promising opportunity to parallelise the multiple manipulations of large single-cell data matrices and speed up calculations. Hence, this is a potential future extension for scShapes to improve the computational time by implementing GPU computing and infer differential distributions of thousands of genes simultaneously."

In short, it is a great suggestion but challenging to implement for the reasons highlighted above.

Close
