# Supplementary material for: scShapes: a statistical framework for identifying distribution shapes in single-cell RNA-sequencing data
Source: Gigascience. 2023 Jan 24;12:giac126. doi: 10.1093/gigascience/giac126 (PMC9871437; doi:10.1093/gigascience/giac126)
Supplement: giac126_Reviewer_1_Report_Original_Submission — Shiping Liu, Ph.D -- 4/10/2022 Reviewed [file giac126_reviewer_1_report_original_submission.pdf]

Reviewer Report

Title: scShapes: A statistical framework for identifying distribution shapes in single-cell RNA-sequencing data.

Version: Original Submission **Date:** 4/10/2022

Reviewer name: Shiping Liu, Ph.D

Reviewer Comments to Author:

How to model the statistical distribution of the gene expression, is a basic question for the field of single cell sequencing data mining. Dharmaratne and colleagues looked details at the distribution of very gene. By using the generalized linear models (GLM), the authors present a new program scShapes, which matched a specific gene with a distribution from one of the four shapes, Poisson, Negative Binomial (NB), Zero-inflated Poisson (ZIP), and Zero-inflated Negative Binomial (ZINB). As the authors present in this manuscript, not all genes adapted to a single distribution, neither NB or Poisson, and some of the genes actually adapted to the zero-inflated models because of the property of high drop-out rate in the modern single cell sequencing, says 3' tag sequenced. It is has been popular to employ GLM in single cell data mining recently, but it also got both praise and blame. So it is a good forward step to model a specific model for an individual gene. But the bad side is the computing cost, especially for the number of cells been sequenced reach to millions in currently research, and it believed that the dataset will be reached even bigger in the future. So it make a great obstacle arise to the application of the method presented by the author here. How to speed up the calculation using the mixed model or scShapes?
The authors also performed the scShapes on some datasets, including the metformin, human T cells, and PBMCs. They found some potential genes that changed the distribution shape, but didn't easy to be identified by other methods. It demonstrated that scShapes could identified the subtle change in gene expression.

Major points:

(1) We didn't see any details about the metformin dataset, the segueing depth and quality, number of genes/UMIs per cell, and so on. It makes hard to evaluate the quality and reliability of the results generated by scShapes. If this dataset is another manuscript could not possible to be presented at the same time, I suggest the author could perform on alternative dataset, as there are so many single cell datasets has been published could be used in this study.

(2) Even the authors taken the cell type account in the GLM, I wonder for a specific gene, whether the distribution shape will change in different cell type. If so, it will becoming more complex, that is need to model the distribution shape for individual gene for every cell type alone.

(3) To identify the different gene expression in scShapes, the author didn't consider the influence of different cell number, or the proportion of cell number, in the different cell type. A possible way to evaluate or eliminate this bias is to down sampling from a big dataset, instead of just simulated total number 2k ~ 5k from the PBMC. To evaluate the influence both the total number cell and the proportion in cell type.

(4) The author should present the comparative results of the computational cost for different methods.

Says the accuracy, time and memory consuming under different number of cells. I suggest the authors use much a larger dataset, because currently single cell research may include millions of cells, and the ability to process big data is very important to the application and becoming a widely used one.
Minor points:
(1) No figure legends for Fig.2 c and d.
(2) It is unclear whether the total 30% genes undergo shape change, or just the proportion of the remaining after the pipeline. So please clarify the details.

Methods

Are the methods appropriate to the aims of the study, are they well described, and are necessary controls included? Choose an item.

Conclusions

Are the conclusions adequately supported by the data shown? Choose an item.

Reporting Standards

Does the manuscript adhere to the journal's guidelines on minimum standards of reporting? Choose an item.

Choose an item.

Statistics

Are you able to assess all statistics in the manuscript, including the appropriateness of statistical tests used? Choose an item.

Quality of Written English

Please indicate the quality of language in the manuscript: Choose an item.

Declaration of Competing Interests

Please complete a declaration of competing interests, considering the following questions:

- Have you in the past five years received reimbursements, fees, funding, or salary from an organisation that may in any way gain or lose financially from the publication of this manuscript, either now or in the future?
- Do you hold any stocks or shares in an organisation that may in any way gain or lose financially from the publication of this manuscript, either now or in the future?
- Do you hold or are you currently applying for any patents relating to the content of the manuscript?

- Have you received reimbursements, fees, funding, or salary from an organization that holds or has applied for patents relating to the content of the manuscript?
- Do you have any other financial competing interests?
- Do you have any non-financial competing interests in relation to this paper?

If you can answer no to all of the above, write 'I declare that I have no competing interests' below. If your reply is yes to any, please give details below.

I declare that I have no competing interests.

I agree to the open peer review policy of the journal. I understand that my name will be included on my report to the authors and, if the manuscript is accepted for publication, my named report including any attachments I upload will be posted on the website along with the authors' responses. I agree for my report to be made available under an Open Access Creative Commons CC-BY license (http://creativecommons.org/licenses/by/4.0/). I understand that any comments which I do not wish to be included in my named report can be included as confidential comments to the editors, which will not be published.

Choose an item.

To further support our reviewers, we have joined with Publons, where you can gain additional credit to further highlight your hard work (see: https://publons.com/journal/530/gigascience). On publication of this paper, your review will be automatically added to Publons, you can then choose whether or not to claim your Publons credit. I understand this statement.

Yes Choose an item.
