# Supplementary material for: scShapes: a statistical framework for identifying distribution shapes in single-cell RNA-sequencing data
Source: Gigascience. 2023 Jan 24;12:giac126. doi: 10.1093/gigascience/giac126 (PMC9871437; doi:10.1093/gigascience/giac126)
Supplement: giac126_Reviewer_2_Report_Original_Submission — Yuchen Yang -- 5/2/2022 Reviewed [file giac126_reviewer_2_report_original_submission.pdf]

Reviewer Report

Title: scShapes: A statistical framework for identifying distribution shapes in single-cell RNA-sequencing data.

Version: Original Submission **Date:** 5/2/2022

Reviewer name: Yuchen Yang

Reviewer Comments to Author:

In this manuscript, authors presented a novel statistical framework scShapes using GLM approach for identifying differential distributions in genes across scRNA-seq data of different conditions. scShapes quantifies gene-specific cell-to-cell variability by testing for differences in the expression distribution. scShapes was shown to be able to identify biologically-relevant switch in gene distribution shapes between different conditions. However, there are still several concerns required to be addressed.

1. In this study, authors compared scShapes to scDD and edgeR. However, besides these two, there are many other methods for calling DEGs from scRNA-seq. Wang et al. (2019) systematically evaluated the performance of eight methods specifically designed for scRNA-seq data (SCDE, MAST, scDD, D3E, Monocle2, SINCERA, DEsingle, and SigEMD) and two methods for bulk RNA-seq (edgeR and DESeq2). Thus, it is also worthy to compare scShapes to other methods, such as SigEMD, DEsingle and DESeq2, which were supposed to perform better than scDD or edgeR.
2. When scShapes was compared to scDD, authors mainly focused on the distribution shifting. However, to users, it would be better to present a venn diagram showing the numbers of the genes detected by both scShapes and scDD, and the genes specifically identified by scShapes and scDD, respectively. In addition, authors showed the functional enrichment results for DEGs identified by scShapes. It is also worthy to perform enrichment analysis for the genes detected by both scShapes and scDD or specifically identified by scShapes or scDD.
3. Since scShapes detects differential gene distribution between different conditions, it would be better to show users how to interpret the significant results biologically. For example, authors mentioned that RXRA is differentially distributed between Old and Young and Old and Treated, so what does this results mean? Can this differential distribution be associated with differential expression?
4. In Discussion, authors mentioned that scRATE is another tool that can model droplet-based scRNA-seq data. It would be clearer to discuss that why authors develop their own algorithm rather than using scRATE to model the distribution.
5. In Introduction, authors talked about the zero counts in scRNA-seq data, and presented evidence in Results part. Since 2020, there are several publications also focusing on this issue, such as Svensson, 2020 and Cao 2021. These discussions should be included in this manuscript.

Methods

Are the methods appropriate to the aims of the study, are they well described, and are necessary controls included? Choose an item.

Conclusions

Are the conclusions adequately supported by the data shown? Choose an item.

Reporting Standards

Does the manuscript adhere to the journal's guidelines on minimum standards of reporting? Choose an item.

Choose an item.

Statistics

Are you able to assess all statistics in the manuscript, including the appropriateness of statistical tests used? Choose an item.

Quality of Written English

Please indicate the quality of language in the manuscript: Choose an item.

Declaration of Competing Interests

Please complete a declaration of competing interests, considering the following questions:

- Have you in the past five years received reimbursements, fees, funding, or salary from an organisation that may in any way gain or lose financially from the publication of this manuscript, either now or in the future?
- Do you hold any stocks or shares in an organisation that may in any way gain or lose financially from the publication of this manuscript, either now or in the future?
- Do you hold or are you currently applying for any patents relating to the content of the manuscript?
- Have you received reimbursements, fees, funding, or salary from an organization that holds or has applied for patents relating to the content of the manuscript?
- Do you have any other financial competing interests?
- Do you have any non-financial competing interests in relation to this paper?

If you can answer no to all of the above, write 'I declare that I have no competing interests' below. If your reply is yes to any, please give details below.

I declare that I have no competing interests

I agree to the open peer review policy of the journal. I understand that my name will be included on my report to the authors and, if the manuscript is accepted for publication, my named report including any attachments I upload will be posted on the website along with the authors' responses. I agree for my

report to be made available under an Open Access Creative Commons CC-BY license (http://creativecommons.org/licenses/by/4.0/). I understand that any comments which I do not wish to be included in my named report can be included as confidential comments to the editors, which will not be published.

Choose an item.

To further support our reviewers, we have joined with Publons, where you can gain additional credit to further highlight your hard work (see: https://publons.com/journal/530/gigascience). On publication of this paper, your review will be automatically added to Publons, you can then choose whether or not to claim your Publons credit. I understand this statement.

Yes Choose an item.
